# BCL-XL regulates the timing of mitotic apoptosis independently of BCL2 and MCL1 compensation

**DOI:** 10.1038/s41419-023-06404-9

**Published:** 2024-01-03

**Authors:** Chun Yin Yu, Tsz Kwan Yeung, Wai Kuen Fu, Randy Y. C. Poon

**Affiliations:** 1 Division of Life Science, The Hong Kong University of Science and Technology, Clear Water Bay, Hong Kong; 2 State Key Laboratory of Molecular Neuroscience, The Hong Kong University of Science and Technology, Clear Water Bay, Hong Kong

**Keywords:** Mitosis, Apoptosis

## Abstract

Mitotic catastrophe induced by prolonged mitotic arrest is a major anticancer strategy. Although antiapoptotic BCL2-like proteins, including BCL-XL, are known to regulate apoptosis during mitotic arrest, adaptive changes in their expression can complicate loss-of-function studies. Our studies revealed compensatory alterations in the expression of BCL2 and MCL1 when BCL-XL is either downregulated or overexpressed. To circumvent their reciprocal regulation, we utilized a degron-mediated system to acutely silence BCL-XL just before mitosis. Our results show that in epithelial cell lines including HeLa and RPE1, BCL-XL and BCL2 acted collaboratively to suppress apoptosis during both unperturbed cell cycle and mitotic arrest. By tagging BCL-XL and BCL2 with a common epitope, we estimated that BCL-XL was less abundant than BCL2 in the cell. Nonetheless, BCL-XL played a more prominent antiapoptotic function than BCL2 during interphase and mitotic arrest. Loss of BCL-XL led to mitotic cell death primarily through a BAX-dependent process. Furthermore, silencing of BCL-XL led to the stabilization of MCL1, which played a significant role in buffering apoptosis during mitotic arrest. Nevertheless, even in a MCL1-deficient background, depletion of BCL-XL accelerated mitotic apoptosis. These findings underscore the pivotal involvement of BCL-XL in controlling timely apoptosis during mitotic arrest, despite adaptive changes in the expression of other BCL2-like proteins.

## Introduction

Anti-microtubule drugs are cornerstones in conventional cancer therapies. These diverse classes of drugs share a common characteristic in their ability to induce protracted mitotic arrest [1]. The consequent mitotic catastrophe, cell death that occurs during mitotic arrest or following aberrant mitotic exit, is critical for the effectiveness of anti-microtubule drugs [2].

During unperturbed cell cycle, mitotic entry is driven by cyclin-dependent kinase 1 (CDK1) and its activating subunit, cyclin B1 [3]. At the end of mitosis, cyclin B1 is destroyed by the ubiquitin ligase anaphase-promoting complex/cyclosome (APC/C) loaded with the targeting subunit CDC20 [4]. Activation of APC/C^CDC20^ is tightly regulated to ensure proper chromosome segregation. This process is initiated only when all the chromosomes have achieved accurate bipolar attachment to the spindles. Unattached kinetochores or the absence of tension between the paired kinetochores activates a spindle-assembly checkpoint (SAC), which inhibits APC/C^CDC20^ through the conversion of MAD2 from an open conformation (O-MAD2) to a closed conformation (C-MAD2). This helps to maintain an active cyclin B1–CDK1 environment during mitosis [5]. Once all the kinetochores are properly attached, new C-MAD2 is no longer generated from the kinetochores. The existing C-MAD2 is converted back to O-MAD2 by a process involving p31^comet^ and TRIP13, which release APC/C^CDC20^ from inhibition by the SAC and allows the cell to exit mitosis [6].

Agents that disrupt microtubule dynamics, such as taxanes and vinca alkaloid, can lead to protracted activation of the SAC and mitotic arrest [1]. However, the outcome after prolonged mitotic arrest can vary greatly between individual cells from the same cell line [7]. Cells can exit mitosis precociously without proper chromosome segregation and cytokinesis in a process termed mitotic slippage. The central event of mitotic slippage appears to be triggered by a gradual degradation of cyclin B1 [8], correlating with a weakening of the SAC over the course of mitotic arrest [9]. Alternatively, apoptosis can be induced during the mitotic arrest, possibly due to the progressive accumulation of apoptotic activators and/or the loss of apoptotic inhibitors [10]. The competition between the rate of mitotic slippage and the accumulation of death signals determines the fate of a cell during prolonged mitotic arrest [7].

The mechanisms underlying the apoptotic signals during mitosis remain poorly understood. However, it is known that several antiapoptotic BCL2-like proteins undergo post-translational modifications and/or changes in abundance during mitosis, potentially playing a role in this process. For example, phosphorylation of BCL2 by cyclin B1–CDK1 [11–14] alters the flexible loop in BCL2, enhancing its binding to BAK and BIM and increasing its antiapoptotic activities during mitotic arrest [15]. Phosphorylation also increases the interaction of BCL2 with the peptidyl prolyl isomerase PIN1 [16, 17]. Nonetheless, the timing of apoptosis during mitotic arrest does not strongly correlate with phosphorylation of BCL2 [13]. Similarly, BCL-XL is phosphorylated by cyclin B1–CDK1, leading to the inactivation of its antiapoptotic activity [18–20].

The functions of BCL2 and BCL-XL in mitotic cell death have been investigated in many studies, with conflicting results. For example, silencing of BCL-XL with RNAi [20–23] or small chemical inhibitors [22–24] has been shown to enhance mitotic cell death. However, mitotic cell death has been reported to be promoted [15, 21], unaffected [20, 22], or blocked [25] with BCL2 RNAi or inhibitors.

The challenge in studying the contribution of antiapoptotic proteins during the cell cycle is that apoptosis or cell cycle delay can occur in a subset of cells before the cell cycle period to be studied. For instance, reduction of mitotic apoptosis after BCL2 downregulation with siRNA could be due to deferred entry into mitosis [25]. Another understudied area is the potential long-term compensation or adaptation from other BCL2 family members when one member is depleted. Other potential issues surrounding loss-of-function studies include incomplete and/or slow kinetics of inhibition and lack of specificity. To overcome these limitations, we demonstrated the relative role of BCL-XL, BCL2, and MCL1 in the timing of mitotic apoptosis using an acute depletion system that combined transcriptional and degron control. These studies revealed a major contribution of BCL-XL in mitotic apoptosis independent on the compensation by BCL2 and MCL1.

## Results

### Reciprocal regulation between BCL2 and BCL-XL

**Fig. 1:**
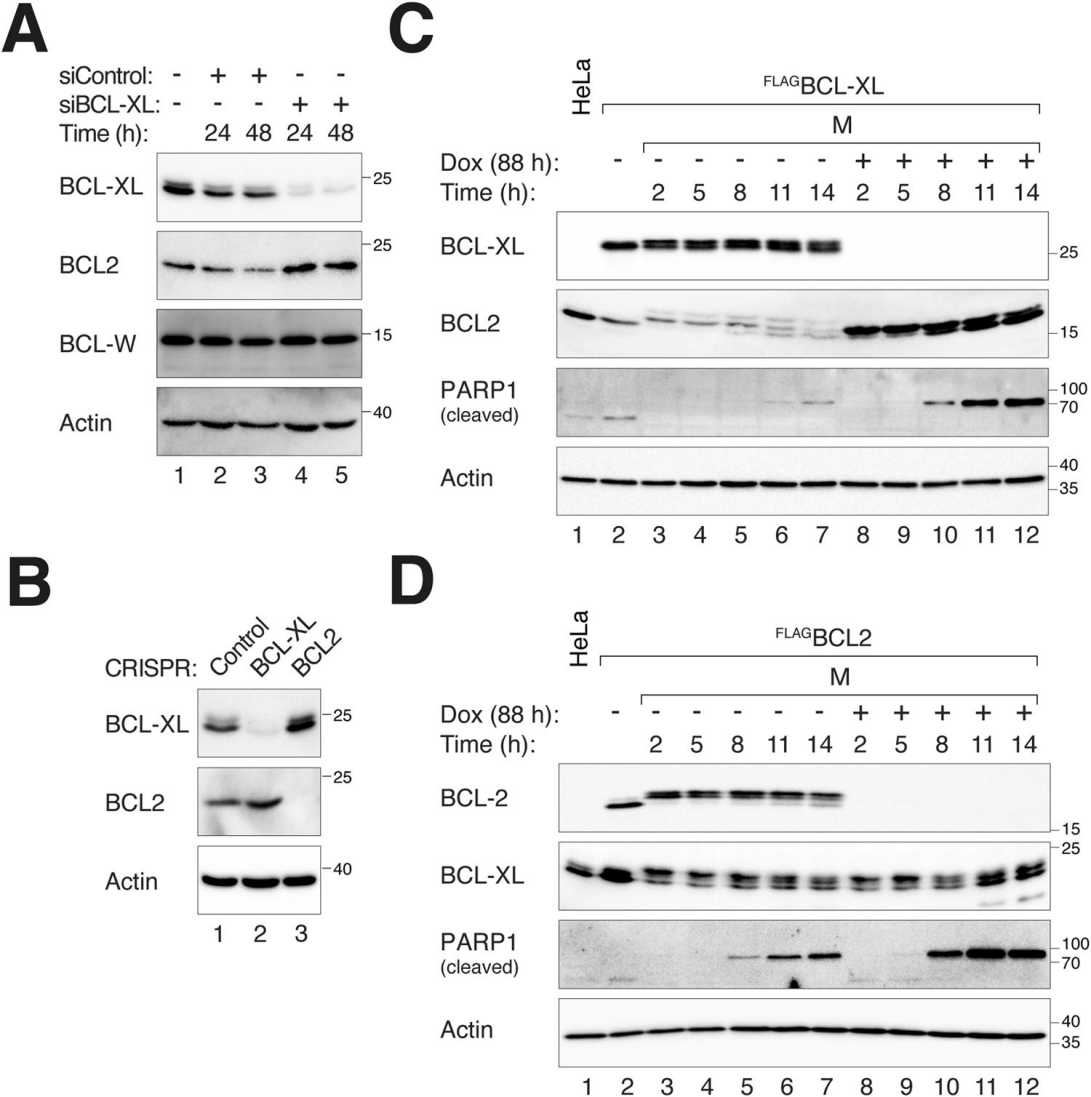
Reciprocal upregulation of BCL2 and BCL-XL after knockdown. **A** Increased expression of BCL2 following downregulation of BCL-XL. HeLa cells were transfected with control (siControl) or siRNA targeting BCL-XL (siBCL-XL) and harvested at the indicated time points. Lysates were prepared and analyzed with immunoblotting. **B** Downregulation of BCL-XL or BCL2 with CRISPR-Cas9 results in an increase in BCL2 and BCL-XL, respectively. Cells were transfected with control plasmids or plasmids expressing CRISPR-Cas9 targeting BCL-XL or BCL2. A plasmid expressing a blasticidin-resistant gene was co-transfected. Transfected cells were enriched with blasticidin selection for 36 h. After 24 h of recovery in normal medium, the cells were harvested for immunoblotting analysis. **C** Long-term overexpression of BCL2 reduces BCL-XL expression. Cells expressing ^FLAG^BCL-XL were left untreated or treated with Dox for 72 h to turn off ^FLAG^BCL-XL. The cells were then synchronized in G_2_ using RO3306 (16 h) before being released in the presence of NOC. Mitotic cells (M) were isolated by shake-off at t = 2 h and further incubated in NOC-containing medium. Lysates were prepared from cells harvested at different time points for immunoblotting analysis. Note that both BCL-XL and BCL2 were phosphorylated during mitotic arrest. Lysates from asynchronously growing parental HeLa cells were loaded for comparison. **D** Overexpression of BCL2 does not reduce BCL-XL expression. Cells overexpressing ^FLAG^BCL2 were generated, synchronized, and treated as described in **C**.

Using data retrieved from DepMap (depmap.org) on more than 1400 cell lines from different tissues of origin, we found that there is a general inverse relationship between the expression of BCL2 and BCL-XL in human cell lines (Fig. S1A). This was corroborated by immunoblotting analysis of the protein expression of BCL2 of BCL-XL in a selection of cell lines (Fig. S1B). Furthermore, the expression of BCL2 was elevated after BCL-XL was depleted with either siRNA (Fig. 1A) or CRISPR-Cas9 (Fig. 1B) in HeLa cells. Similarly, downregulation of BCL2 using CRISPR-Cas9 resulted in an increase in BCL-XL expression (Fig. 1B).

To further investigate the relationship between BCL2 and BCL-XL, we examined the effects of overexpression of these proteins. We found that cells stably expressing ^FLAG^BCL-XL contained lower levels of endogenous BCL2 than control cells during both interphase and mitotic block (Fig. 1C). As the ^FLAG^BCL-XL was under the control of an inducible Tet-Off promoter, it could be repressed by growing the cells in the presence of doxycycline (Dox). Turning off ^FLAG^BCL-XL (for 88 h) reversed BCL2 expression to background levels, indicating the reversibility of the BCL2 adaptive responses. PARP1 cleavage analysis confirmed that overexpression of BCL-XL reduced apoptosis in mitotic-arrested cells. However, the converse overexpression of BCL2 did not result in a reduction in BCL-XL (Fig. 1D), indicating the inverse relationship between BCL-XL and BCL2 occurred under specific conditions.

Taken together, these results suggest the presence of potential compensatory changes in BCL2 when the expression BCL-XL is either reduced or overexpressed.

### Acute silencing of BCL2 and BCL-XL circumvents their reciprocal regulation

**Fig. 2:**
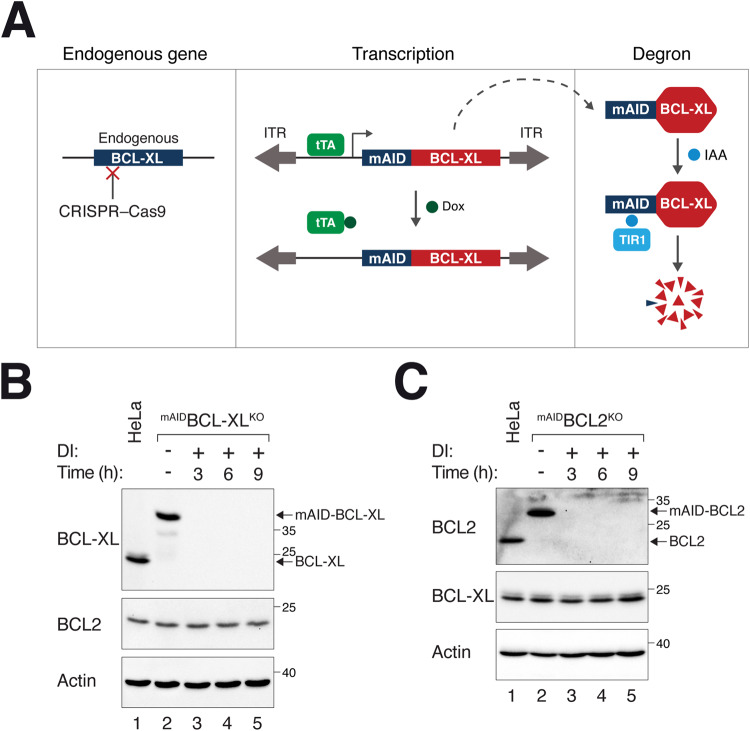
Rapid and tight conditional silencing of BCL2 and BCL-XL. **A** Overview of conditional gene silencing of BCL2 and BCL-XL. The endogenous locus of BCL2 or BCL-XL (only BCL-XL is shown in the Figure) was disrupted using CRISPR-Cas9. The BCL-XL cDNA was tagged with mAID and put inside a Sleeping Beauty transposon cassette for delivery to the genome to rescue the KO effects (ITR: inverted terminal repeat). Silent mutations were introduced into the cDNA to confer resistance to the CRISPR-Cas9. The tetracycline-controlled transcriptional activator (tTA) binds to the TRE in the promoter and activates the transcription of ^mAID^BCL-XL in the absence of Dox. Addition of Dox turns off the transcription. In response to IAA, residual ^mAID^BCL-XL was targeted to degradation in cells expressing TIR1. **B** Generation of ^mAID^BCL-XL^KO^ cells. HeLa cells stably expressing ^mAID^BCL-XL, tTA, and TIR1 were established. The endogenous BCL-XL was concurrently disrupted using CRISPR-Cas9. A single-colony-derived clone with similar expression of ^mAID^BCL-XL to endogenous BCL-XL was isolated. The cells were cultured in the presence or absence of Dox and IAA (DI) and harvested at the indicated time points. Lysates were prepared and analyzed with immunoblotting. Lysates from HeLa were also loaded to indicate the expression of endogenous BCL-XL. Equal loading of lysates was confirmed by immunoblotting for actin. **C** Generation of ^mAID^BCL2^KO^ cells. Cells were generated as described in panel B, except that BCL2 was targeted instead of BCL-XL.

The above results highlighted potential limitations of studying BCL2 family using loss-of-function approaches including RNAi. Additional shortcomings of tactics involving RNAi include incomplete knockdown, lack of specificity, and relatively slow responses. To achieve more acute and tight silencing of BCL2 and BCL-XL, we developed cell lines based on a conditional dual transcription–degron system [26, 27] (Fig. 2A). Concurrent with the disruption of BCL2/BCL-XL with CRISPR-Cas9, a mini auxin-induced degron (mAID)-tagged BCL2/BCL-XL under the control of a Tet-Off promoter was delivered to the genome using Sleeping Beauty transposase. Silencing of mAID-tagged BCL2/BCL-XL could be achieved by turning off the promoter using Dox and targeting existing proteins to proteolysis using indole-3-acetic acid (IAA).

Our initial trials revealed that the expression levels of ^mAID^BCL-XL and ^mAID^BCL2 were substantially higher than the endogenous proteins in HeLa cells. To mitigate this issue, we appended different number of AU-rich elements [28] at the 3’-UTR to destabilize the mRNA and generated single-cell derived clones that expressed mAID-tagged proteins at levels comparable to those of the endogenous proteins (Fig. S2A, B). Figure 2B shows that in cells lacking endogenous BCL-XL and expressing ^mAID^BCL-XL (designated as ^mAID^BCL-XL^KO^ herein) were able to degrade ^mAID^BCL-XL in response to Dox and IAA treatment (DI), in effect producing a BCL-XL-deficient environment. Notably, the silencing was rapid, with ^mAID^BCL-XL becoming undetectable within 3 h after addition of DI. It is noteworthy that the destruction of ^mAID^BCL-XL did not alter the abundance of BCL2 during this relatively short time window. Similarly, ^mAID^BCL2 in ^mAID^BCL2^KO^ cells could be degraded rapidly without affecting the abundance of BCL-XL (Fig. 2C).

These data demonstrate that systems for acute depletion of BCL-XL and BCL2 can serve as effective tools for studying apoptosis during mitosis, circumventing the potential complications associated with long-term gene compensation.

### Both BCL2 and BCL-XL are involved in suppressing apoptosis during the unperturbed cell cycle

**Fig. 3:**
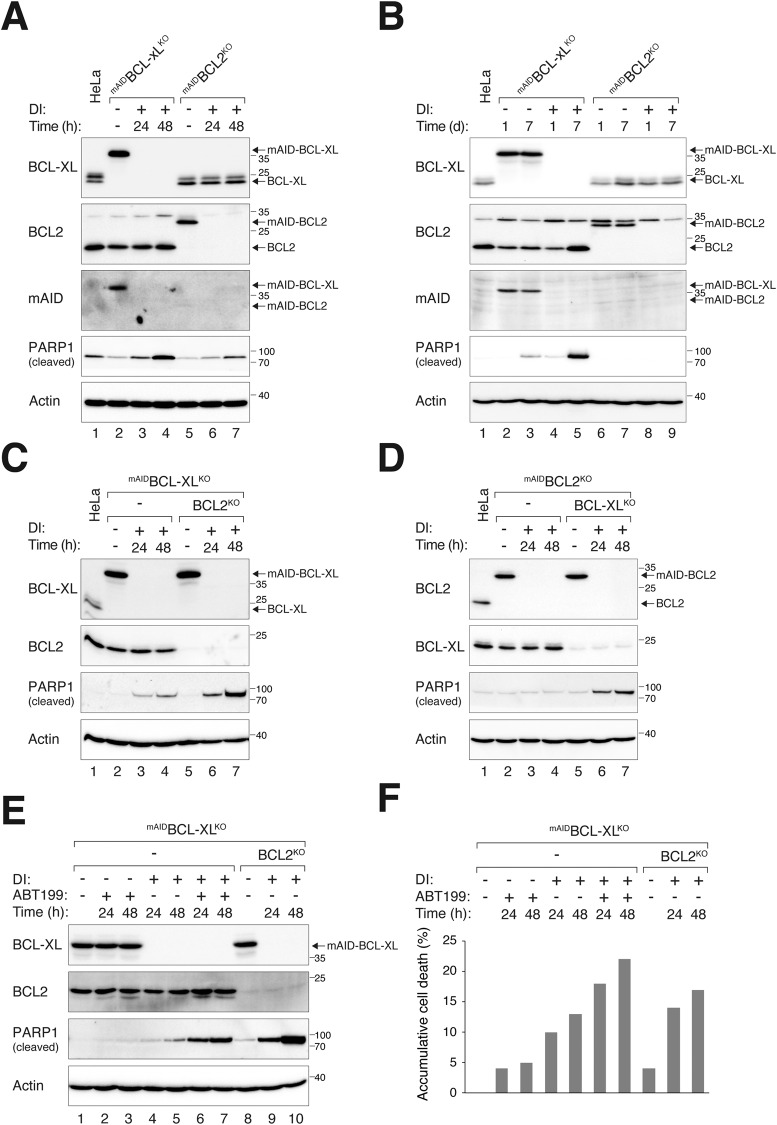
Both BCL2 and BCL-XL are involved in buffering apoptosis during unperturbed cell cycle. **A** BCL-XL plays a more prominent role in restraining apoptosis than BCL2 during interphase. ^mAID^BCL-XL^KO^ and ^mAID^BCL2^KO^ cells were either left untreated or treated with DI to turn off the mAID-tagged proteins. After 24 h and 48 h, the cells were harvested and analyzed with immunoblotting. **B** Induction of apoptosis after destruction of BCL-XL. ^mAID^BCL-XL^KO^ and ^mAID^BCL2^KO^ cells were left untreated or treated with DI for up to 7 days. The cells were harvested on the indicated days and analyzed with immunoblotting. **C** Increased apoptosis in cells lacking both BCL-XL and BCL2 compared to cells lacking only BCL-XL. ^mAID^BCL-XL^KO^ cells were transfected with CRISPR-Cas9 targeting BCL2. The double-KO and original ^mAID^BCL-XL^KO^ cell lines were treated with DI and harvested at the indicated time points. Lysates were prepared and analyzed with immunoblotting. **D** Cells lacking both BCL-XL and BCL2 undergo more apoptosis than cells lacking only BCL2. ^mAID^BCL2^KO^ cells were transfected with CRISPR-Cas9 targeting BCL-XL and a mixed population was established. The double-KO and original ^mAID^BCL2^KO^ cell lines were treated with DI and harvested at the indicated time points. Lysates were prepared and analyzed with immunoblotting. **E** BCL-XL deficiency enhances sensitivity to the BCL2 inhibitor ABT199. ^mAID^BCL2^KO^ cells were treated with DI and/or ABT199 and harvested at the indicated time points. ^mAID^BCL-XL^KO^ cells without BCL2 (see **C**) were treated with DI. Lysates were prepared and analyzed with immunoblotting. **F** Quantification of cell death with live-cell imaging in cells subjected to the treatments described in **E** (*n* = 50).

Using the above system, we next examined the relative contribution of BCL2 and BCL-XL in buffering apoptosis during the unperturbed cell cycle. The depletion of either protein led to an increase in apoptosis, as indicated by the accumulation of cleaved PARP1 (Fig. 3A). Notably, the absence of BCL-XL resulted in more PARP1 cleavage compared to the absence of BCL2, suggesting that BCL-XL plays a more critical antiapoptotic function than BCL2 in unperturbed HeLa cells. Cleaved PARP1 accumulated in a time-dependent manner after turning off ^mAID^BCL-XL (Fig. 3B). Endogenous BCL2 also accumulated at 7 days after the destruction of ^mAID^BCL-XL, further supporting the possibility of a compensatory mechanism involving long-term BCL2 upregulation following BCL-XL depletion.

We next established BCL2 and BCL-XL double-knockout (KO) cells by disrupting BCL2 with CRISPR-Cas9 in ^mAID^BCL-XL^KO^ (Fig. 3C), as well as disrupting BCL-XL in ^mAID^BCL2^KO^ (Fig. 3D). Upon turning off ^mAID^BCL-XL, the double-KO cells showed higher levels of cleaved PARP1 compared to cells lacking only BCL-XL (Fig. 3C). Likewise, more cleaved PARP1 was detected in double-KO cells than in cells lacking only BCL2 (Fig. 3D). Consistent with these results, treatment of ^mAID^BCL-XL^KO^ cells with the specific BCL2 inhibitor ABT-199 (Venetoclax) induced apoptosis only after ^mAID^BCL-XL was degraded. Side-by-side analysis revealed that the levels of cleaved PARP1 (Fig. 3E) and accumulative cell death (analyzed with live-cell imaging; Fig. 3F) induced by ABT-199 were comparable to cells lacking both BCL-XL and BCL2.

One plausible explanation of the dominant role of BCL-XL over BCL2 is that HeLa cells may contain more BCL-XL than BCL2. Using an antibody specific for the mAID tag, we compared side-by-side the expression of mAID-tagged proteins in ^mAID^BCL-XL^KO^ and ^mAID^BCL2^KO^ cells. The expression of ^mAID^BCL-XL and ^mAID^BCL2 was then compared to endogenous BCL-XL and BCL2 in HeLa cells using BCL-XL- and BCL2-specific antibodies, respectively (Fig. S3A). Surprisingly, these analyses revealed that endogenous BCL-XL was less abundant than BCL2 (although ^mAID^BCL-XL was expressed at a higher level than ^mAID^BCL2 (Fig. 3A, B), ^mAID^BCL-XL was expressed at a level higher than endogenous BCL-XL). Using a bacterially expressed recombinant mAID (mAID-H6) as a standard (Fig. S3B), we estimated that BCL-XL and BCL2 were present in HeLa lysates at concentrations of 0.03 ng/µg and 0.08 ng/µg, respectively (Fig. S3C). These results suggest that the more dominant antiapoptotic role of BCL-XL compared to BCL2 cannot be simply due to its higher abundance.

Taken together, these data indicate that BCL-XL and BCL2 act collaboratively to suppress apoptosis, with BCL-XL playing a more prominent antiapoptotic function than BCL2 during the unperturbed cell cycle.

### BCL-XL is critical for mitotic apoptosis regardless of the compensation by BCL2

**Fig. 4:**
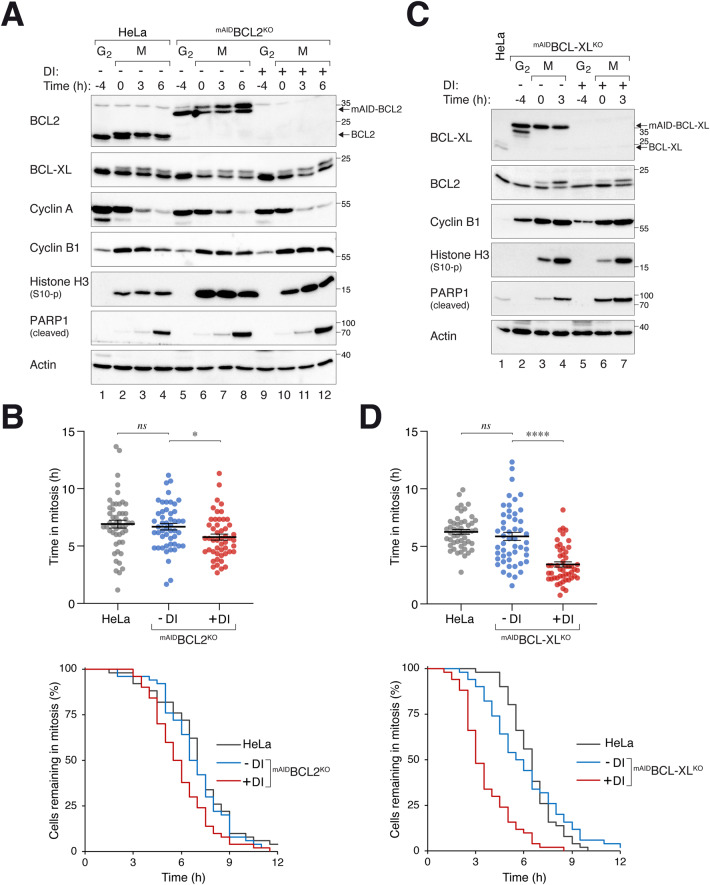
BCL-XL is a critical regulator of mitotic apoptosis. **A** Comparable PARP1 cleavage during mitotic arrest in the presence or absence of BCL2. Parental HeLa and ^mAID^BCL2^KO^ cells were synchronized using a double thymidine procedure. G_2_ samples were harvested at 8 h after release from the second thymidine block (indicated as t = -4 h). At the time of the second thymidine release, the cells were either left untreated or treated with DI and trapped in mitosis using NOC. Mitotic cells (M) were isolated by shake-off (t = 0) and further incubated in NOC-containing medium. The cells were then harvested at different time points and analyzed with immunoblotting. **B** Marginal acceleration of mitotic apoptosis in BCL2-deficient cells. HeLa and ^mAID^BCL2^KO^ cells were synchronized and arrested in mitosis as in panel A. Individual cells were tracked using live-cell imaging. Dot plots show the elapsed time between mitotic entry and apoptosis (*n* = 50; mean-/+SEM). The duration of mitotic arrest is plotted using Kaplan-Meier estimator. Mann-Whitney test: *p < 0.05; ns *p* > 0.05. Raw data for individual cells are shown in Fig. S4A. **C** Increased PARP1 cleavage during mitotic arrest in BCL-XL-deficient cells. ^mAID^BCL-XL^KO^ cells were synchronized and arrested in mitosis as described in panel A. Protein expression was analyzed with immunoblotting. **D** Acceleration of mitotic apoptosis in BCL-XL-deficient cells. HeLa and ^mAID^BCL-XL^KO^ cells were synchronized and arrested in mitosis as described in panel A. The cells were left untreated or treated with DI at the time of second thymidine release. Individual cells were tracked using live-cell imaging. Dot plots show the elapsed time between mitotic entry and apoptosis (*n* = 50; mean-/+SEM). The duration of mitotic arrest is plotted using Kaplan-Meier estimator. Mann-Whitney test: ****p < 0.0001; ns *p* > 0.05. Raw data for individual cells are shown in Fig. S4B.

Given that depletion of BCL-XL or BCL2 resulted in a subset of cells undergoing apoptosis during the unperturbed cell cycle, mitotic cell death was studied by first synchronizing cells in G_2_ (with a double thymidine or RO3306 procedure) before exposing the cells to the microtubule-disrupting agent nocodazole (NOC). Mitotic cells were then isolated and incubated further in NOC-containing medium. Mitotic arrest was confirmed by the destruction of cyclin A and the accumulation of cyclin B1 and histone H3^Ser10^ phosphorylation (Fig. 4A). Gel mobility shifts of BCL2 and BCL-XL further confirmed the mitotic arrest.

In ^mAID^BCL2^KO^ cells, DI was added during the release from the second thymidine block to turn off ^mAID^BCL2 (Fig. 4A). Similar level of PARP1 cleavage was induced in the presence or absence of ^mAID^BCL2, indicating that BCL2 was not critical for regulating mitotic apoptosis. In agreement with this, single-cell analysis using live-cell imaging showed only a modest acceleration of mitotic apoptosis (Fig. 4B). Moreover, BCL-XL remained relatively constant during the mitotic arrest in the presence or absence of BCL2, excluding the possibility of a compensatory accumulation of BCL-XL (Fig. 4A).

By contrast, silencing of BCL-XL in ^mAID^BCL-XL^KO^ cells enhanced apoptosis during mitotic arrest, as indicated by PARP1 cleavage analysis (Fig. 4C) and live-cell imaging (Fig. 4D). We also generated ^mAID^BCL-XL^KO^ cells using the normal TERT-immortalized RPE1 (Fig. S5A) and found that a similar acceleration of mitotic apoptosis in the absence of BCL-XL (Fig. S5B, C), indicating the importance of BCL-XL in repressing mitotic apoptosis is not restricted to HeLa cells.

**Fig. 5:**
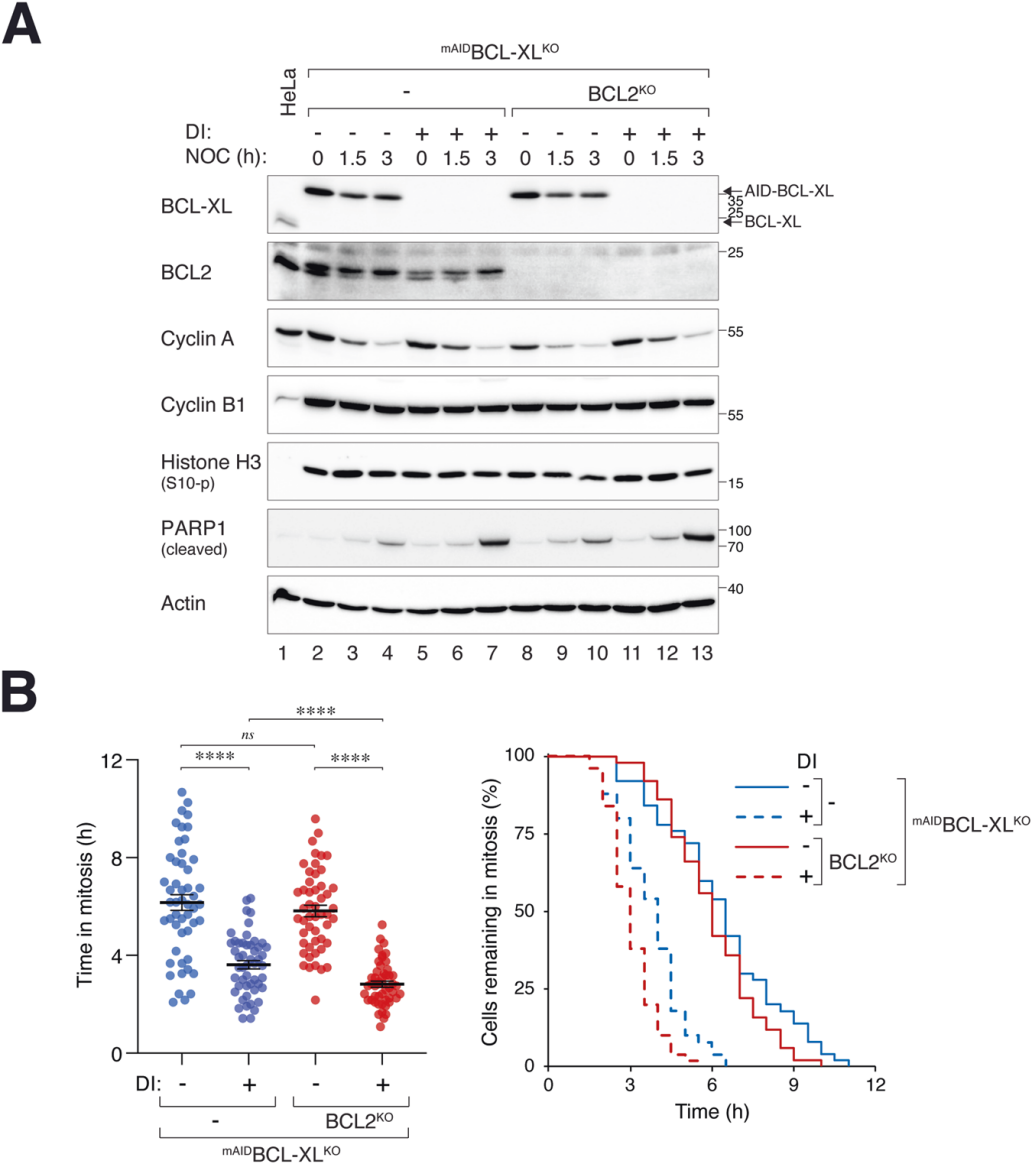
BCL2 acts in concert with BCL-XL to regulate mitotic cell death. **A** Increased mitotic apoptosis in cells lacking both BCL-XL and BCL2. ^mAID^BCL-XL^KO^ and BCL2-deficient ^mAID^BCL-XL^KO^ cells were synchronized and arrested in mitosis as described in Fig. 4A. The cells were left untreated or treated with DI at the time of second thymidine release. Lysates were prepared and analyzed with immunoblotting. **B** Additive effects of BCL2 and BCL-XL on the timing of mitotic apoptosis. ^mAID^BCL-XL^KO^ and BCL2-deficient ^mAID^BCL-XL^KO^ cells were synchronized and arrested in mitosis as described in Fig. 4A. The cells were left untreated or treated with DI at the time of second thymidine release. Individual cells were tracked using live-cell imaging for 24 h, starting at 2 h after thymidine release (*n* = 50). Dot plots show the elapsed time between mitotic entry and apoptosis (mean±SEM). The duration of mitotic arrest is plotted using Kaplan-Meier estimator. Mann-Whitney test: ****p < 0.0001. Raw data for individual cells are shown in Fig. S6.

Finally, KO of BCL2 in a BCL-XL-deficient background only marginally increased PARP1 cleavage (Fig. 5A) and accelerated cell death (by ~1 h; Fig. 5B) during mitotic arrest. These data further support the relatively minor contribution of BCL2 relative to BCL-XL on the timing mitotic cell death.

Collectively, these results indicate that BCL-XL plays a critical role in buffering apoptosis during mitotic arrest, while BCL2 exerts a relatively minor effect on the timing of this process.

### Loss of BCL-XL promotes mitotic apoptosis mainly through BAX

**Fig. 6:**
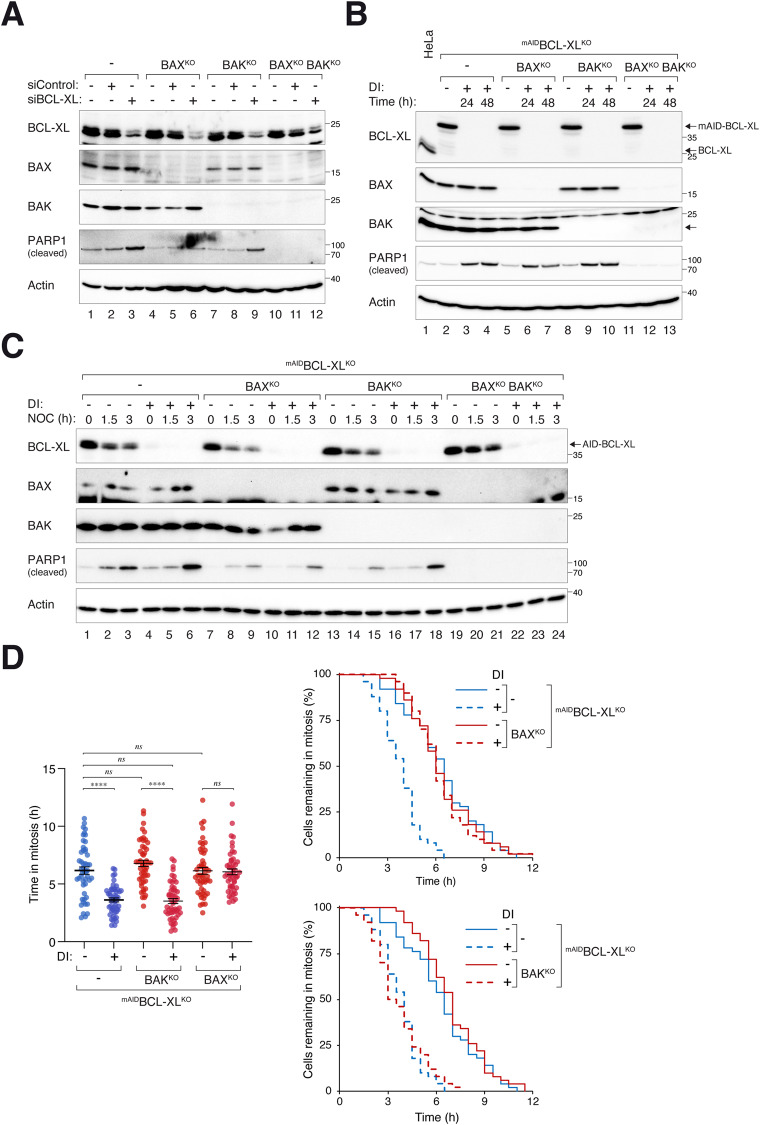
Loss of BCL-XL induces BAX-dependent apoptosis during mitotic arrest. **A** BAK and BAX are required for apoptosis induced by BCL-XL knockdown. HeLa cells were transfected with CRISPR-Cas9 plasmids targeting BAK and/or BAX. After selection, the mixed population of cells was transfected with control or BCL-XL siRNA and harvested after 24 h. Lysates were prepared and analyzed with immunoblotting. **B** Dependence of apoptosis on BAK and BAX in BCL-XL-deficient cells. BAK- and/or BAX-deficient cells were established in ^mAID^BCL-XL^KO^ cells. The cell lines were treated with DI to turn off BCL-XL and harvested at the indicated time points for immunoblotting analysis. **C** BAX is critical for mitotic apoptosis triggered by BCL-XL silencing. Parental ^mAID^BCL-XL^KO^ and ^mAID^BCL-XL^KO^ without BAX and/or BAK were synchronized and arrested in mitosis as described in Fig. 4A. The cells were left untreated or treated with DI at the time of second thymidine release to inhibit BCL-XL. Protein expression was analyzed with immunoblotting. **D** Parental ^mAID^BCL-XL^KO^ and ^mAID^BCL-XL^KO^ without BAX and/or BAK were synchronized and arrested in mitosis as before. The cells were left untreated or treated with DI at the time of second thymidine release. Individual cells were tracked using live-cell imaging. Dot plots show the elapsed time between mitotic entry and apoptosis (*n* = 50; mean±SEM). The duration of mitotic arrest is plotted using Kaplan-Meier estimator. Mann-Whitney test: ****p < 0.0001; ns: *p* > 0.05. Raw data for individual cells are shown in Fig S6.

To determine which pro-apoptotic BCL2-like proteins are involved in mitotic cell death opposing BCL-XL, we next deleted BAK and/or BAX using CRISPR-Cas9. Apoptosis associated with BCL-XL siRNA was reduced after KO of BAK or BAX, and abolished when both BAK and BAX were disrupted together (Fig. 6A). Consistent with these findings, BCL-XL-deficiency-mediated apoptosis in ^mAID^BCL-XL^KO^ cells was abolished after deleting both BAK and BAX (Fig. 6B). Both approaches also indicated that BAX has a marginally stronger pro-apoptotic effect than BAK during interphase.

When BCL-XL-deficient cells were synchronized in mitosis, KO of BAX exerted a stronger effect on preventing mitotic apoptosis than KO of BAK (Fig. 6C). Mitotic apoptosis was further inhibited when both BAK and BAX were disrupted together. The predominantly BAX-dependent mitotic apoptosis in BCL-XL-deficient cells was also apparent when cell fates were analyzed using live-cell imaging (Fig. 6D). These data indicate that mitotic apoptosis associated with loss of BCL-XL is primarily due to a BAX-dependent process.

### BCL-XL is critical for mitotic apoptosis regardless of the compensation by MCL1

Unlike other members of the BCL2 family, MCL1 (Myeloid cell leukemia-1) is unstable during mitotic arrest [29]. MCL1 is destroyed during mitotic arrest in a proteasome-dependent manner [10, 30]. As MCL1 was present at a higher level than BCL-XL (Fig. S3C), the progressive destruction of MCL1 can potentially shift the balance of apoptotic signals during mitotic arrest to favor apoptosis.

**Fig. 7:**
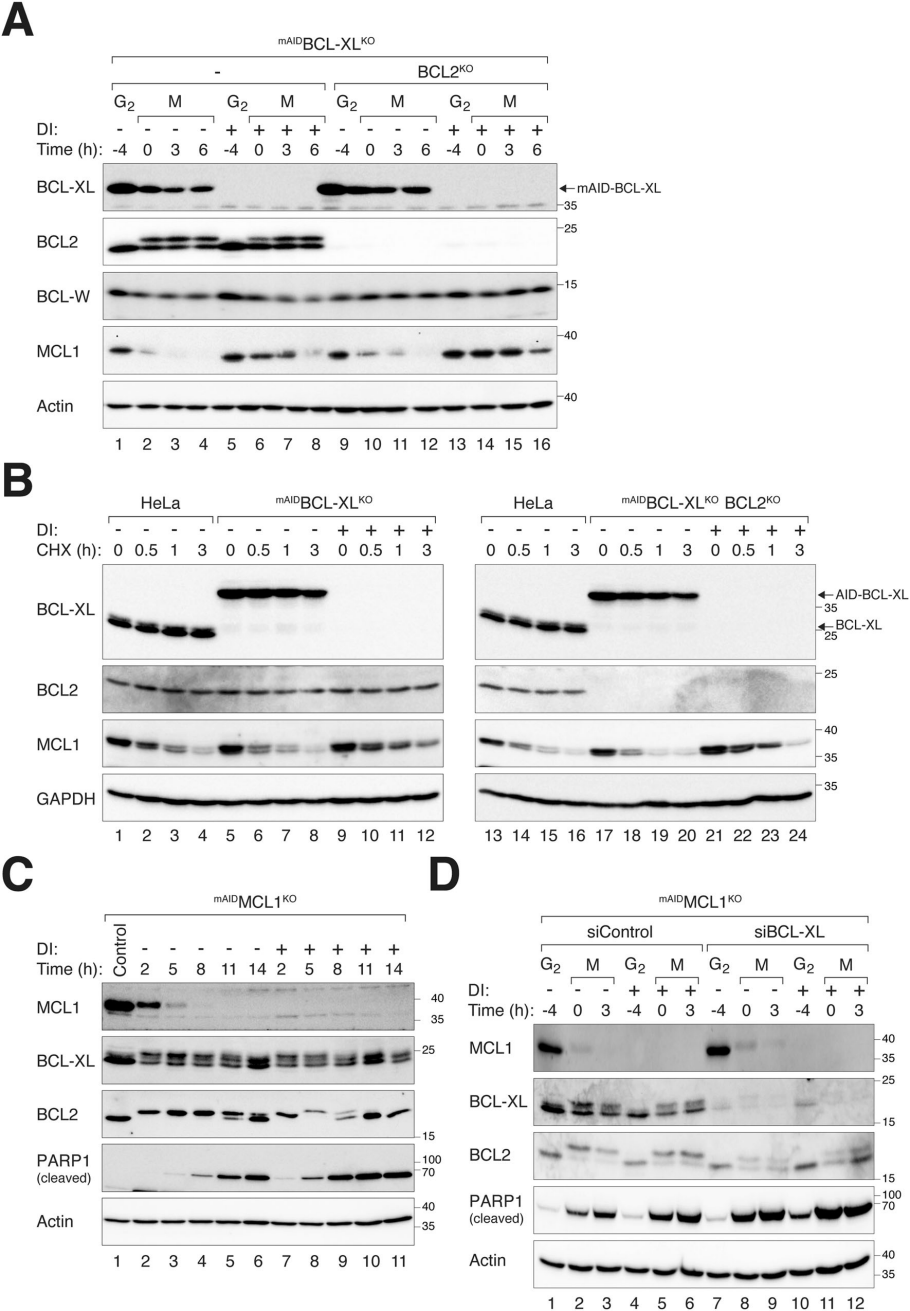
BCL-XL is critical for mitotic apoptosis independent of compensation by MCL1. **A** Stabilization of MCL1 in the absence of BCL-XL. ^mAID^BCL-XL^KO^ and BCL2-deficient ^mAID^BCL-XL^KO^ cells were synchronized in G_2_ and mitotic arrest as described in Fig. 4A. The cells were left untreated or treated with DI at the time of second thymidine release. Lysates were prepared and analyzed with immunoblotting. **B** Enhanced stability of MCL1 during mitosis in the absence of BCL-XL. Parental HeLa, ^mAID^BCL2^KO^, or BCL2-deficient ^mAID^BCL-XL^KO^ cells were left untreated or treated with DI for 24 h. Translation was inhibited using CHX, and the cells were harvested at different time points. Protein expression was assessed with immunoblotting. Equal loading of lysates was confirmed by immunoblotting for GAPDH. **C** MCL1 delays apoptosis during mitotic arrest. ^mAID^MCL1^KO^ cells were synchronized at G_2_ by incubation with RO3306 for 16 h. The cells were left untreated or treated with DI at the same time to turn off MCL1. The cells were then released into mitosis in the presence of NOC. The cells were harvested at the indicated time points and analyzed with immunoblotting. **D** BCL-XL is crucial for timely mitotic apoptosis even in the absence of MCL1. ^mAID^MCL1^KO^ cells were transfected with control or siRNA against BCL-XL, before synchronized in G_2_ and mitotic arrest. The cells were left untreated or treated with DI at the time of second thymidine release to turn off MCL1. Lysates were prepared and analyzed with immunoblotting.

Interesting, we found that the loss of BCL-XL increased the stability of MCL1 during mitotic arrest. More MCL1 was detected during mitotic arrest after ^mAID^BCL-XL was turned off in ^mAID^BCL-XL^KO^ cells (Fig. 7A). A similar stabilization of MCL1 was also observed in ^mAID^BCL-XL^KO^ cells lacking BCL2 (Fig. 7A). By contrast, the expression of BCL-W was unaffected by BCL-XL depletion (Figs. 1A and 7A). Using cycloheximide (CHX) to inhibit protein translation, we found that the stability of MCL1 was increased in cells after BCL-XL was turned off (Fig. 7B).

To demonstrate that MCL1 can play a role in mitotic apoptosis, we analyzed mitotic cell death in a ^mAID^MCL1^KO^ cell line established previously (similar to the other cell lines used in this study, ^mAID^MCL1^KO^ cells lacked endogenous MCL1 and expressed ^mAID^MCL1 under the control of an inducible promoter and the mAID degron). Figure 7C shows that apoptosis was accelerated during mitotic arrest after MCL1 was silenced. These data are consistent with the idea that although MCL1 is degraded later during mitotic arrest, variation in its stability is sufficient to affect the rate of apoptosis during mitotic arrest. Finally, we depleted BCL-XL in ^mAID^MCL1^KO^ cells and found that silencing of BCL-XL further promoted apoptosis in MCL1-depleted cells during mitotic arrest (Fig. 7D). Conversely, turning off ^mAID^MCL1 in the absence of BCL-XL also promoted apoptosis. These results indicate that both BCL-XL and MCL1 independently contribute to the buffering of apoptosis during mitotic arrest.

## Discussion

Conceptually, the presence of long-term gene adaptation can make interpretations of studies of gene functions by knockout or knockdown approaches challenging. In the context of apoptosis associated with mitotic arrest, the timing of apoptosis is likely to be controlled by a complex interplay between pro- and antiapoptotic factors. This complexity, together with adaptation mechanisms, has the potential to shift the outcome of mitotic arrest from apoptosis to mitotic slippage [2].

The importance of antiapoptotic BCL2 family in controlling the timing of apoptosis during mitotic arrest has been well-documented in numerous studies. In the HeLa cell model used in this study, we confirmed that silencing of BCL-XL (Fig. 4C, D), BCL2 (Fig. 4A, B), and MCL1 (Fig. 7C) could accelerate mitotic apoptosis. These results indicate the contributions of these proteins to antiapoptotic activities during mitotic arrest. Notably, our results indicate that at least in epithelial cells, BCL-XL plays the most critical role in mitotic apoptosis, followed by MCL1 and then BCL2. Using the Kaplan-Meier plots and measuring the time required for half of the cells to die during mitotic arrest, we found that turning off ^mAID^BCL-XL reduced this time by ~45% (Fig. 4D). By contrast, turning off ^mAID^MCL1 [10] and ^mAID^BCL2 (Fig. 4B) shortened the time by ~30% and ~15%, respectively.

Several lines of evidence support the presence of adaptation mechanisms from BCL2 when the expression BCL-XL is altered. Firstly, BCL2 expression was increased after transfection of siRNA targeting BCL-XL (Fig. 1A). A similar increase of BCL2 was observed when BCL-XL was disrupted using CRISPR-Cas9 (Fig. 1B). Secondly, overexpression of BCL-XL resulted in a reduction of BCL2 (Fig. 1C). Finally, the endogenous BCL2 accumulated at 7 days after the destruction of ^mAID^BCL-XL (Fig. 3B). As a comparison, BCL-W expression was unchanged upon depletion of BCL-XL (Figs. 1A and 7A).

An interesting question is whether upregulated BCL2 could influence apoptosis in epithelial cells. HeLa and RPE1 contain relatively high expression of BCL2 among the epithelial cell lines we examined (Fig. S1B). Moreover, we estimated that BCL2 is more abundant than BCL-XL in HeLa cells (Fig. S3). It should be noted that the method for estimating the relative abundance of BCL-XL and BCL2 relies on the assumption that fusion of BCL2 or BCL-XL does not affect the recognition of the mAID tag by the antibodies used in immunoblots. In this study, we found that destruction of ^mAID^BCL2 alone only marginally increased apoptosis during interphase (Fig. 3A, D). By contrast, PARP1 cleavage was readily detectable after ^mAID^BCL-XL destruction both after 24 h and up to 7 days (Fig. 3A, B). However, more apoptosis was induced after ^mAID^BCL-XL destruction in a BCL2^KO^ background (Fig. 3C), indicating that BCL2 plays a role in apoptosis in the absence of BCL-XL during the unperturbed cell cycle. As discussed above, silencing of ^mAID^BCL2 shortened the 50% death point during mitotic block by ~15% (Fig. 4B). Similarly, knockout of BCL2 also accelerated mitotic apoptosis to a similar extent in BCL-XL-deficient cells (Fig. 5B).

Similar to BCL2, MCL1 was stabilized in the absence of BCL-XL (Fig. 7A). MCL1 stabilization occurred more rapidly compared to BCL2, as an increase in MCL1 could be detected shortly after degradation of ^mAID^BCL-XL in ^mAID^BCL-XL^KO^ cells (Fig. 7A, B). By contrast, BCL2 stabilization was only observed several days after BCL-XL silencing (Fig. 3B). This difference may be attributed to the ubiquitylation and rapid turnover of MCL1 mediated by multiple ubiquitin ligases [31]. Despite its degradation during prolonged mitotic arrest, MCL1 significantly influences the timing of mitotic apoptosis (Fig. 7C). Furthermore, MCL1 is likely to be more abundant than BCL-XL during interphase (Fig. S3), suggesting that the increase in MCL1 may compensate for some of BCL-XL’s antiapoptotic functions during mitotic arrest. Although ectopic expression of MCL1 can delay apoptosis during mitotic arrest [21], it remains to be determined whether the more modest increase of MCL1 seen after BCL-XL depletion can affect mitotic apoptosis. However, it is clear that BCL-XL plays a pivotal role in controlling apoptosis even in a MCL1-deficient background (Fig. 7D).

The combination of antimitotic drugs with BH3-mimetics has been shown to significantly improve responses in various solid tumors, including breast, gastric, non-small cell lung, and ovarian cancer [32]. Notably, a systematic analysis of the BCL-XL/BCL2/BCL-W inhibitor Navitoclax’s ability to enhance the efficacy of clinically relevant agents across a spectrum of solid tumors revealed the strongest synergy with antimitotic drugs [33]. An implication of the present study is that although inhibition of BCL2-like proteins can sensitize cancer cells to antimitotic drugs, prolonged inhibition may result in adaptative changes and potentially reduce the effectiveness of these drugs.

## Methods

### Plasmids

CRISPR-Cas9 plasmids were generated by annealing the indicated pairs of oligonucleotides followed by ligation into BbsI-cut pX330 (a gift from Feng Zhang; obtained from Addgene, Cambridge, MA, USA; Addgene#42230): BCL2 (5′‑CACCGTGGGAAGGATGGCGCACGCT‑3′ and 5′‑AAACAGCGTGCGCCA TCCTTCCCAC‑3′); BCL-XL (5′‑CACCGAGTTTGAACTGCGGTACCGG‑3′ and 5′‑AAACCCGGTACCGCAGTTCAAACTC‑3′); BAX (5′‑CACCGCTGCAGGATGATTGCCGCCG‑3′ and 5′‑AAACCGGCGGCAATCATCCTGCAGC‑3′), and BAK (5′‑CACCGACGGCAGCTCGCCATCATCG‑3′ and 5′‑AAACCGATGATGGCGAGCTGCCGTC‑3′).

AU-rich elements were added to pUHD-SB-mAID/Hyg vector [27] by ligating the annealed product of the oligonucleotides (5′‑AATTCGAGGATCCATTTATTTATTTATTTATTTA‑3′ and 5′‑GATCTAAATAAATAAATAAATAAATGGATCCTCG‑3′) into EcoRI- and BamHI-cut pUHD-SB-mAID/Hyg to generate pUHD-SB-mAID-AU/Hyg. The same procedures were repeated to obtain additional repeats (up to four) of the AU-rich elements.

BCL2 in pCMV-SPORT6 was obtained from Geneservice (Cambridge, UK; IMAGE clone 4511027). The BCL2 cDNA was amplified with PCR (primers 5′‑AACCATGGCGCACGCTGGGAGAA‑3′ and 5′‑TGGAATTCTCACTTGTGGCCCAGATA‑3′); the PCR product was then cut with NcoI and EcoRI and ligated into pUHD-SB-mAID-AU/Hyg or pUHD-SB-mAID-AU/Hyg vectors with different AU repeats to generate mAID-BCL2 in pUHD-SB-mAID/Hyg or mAID-BCL2 in pUHD-SB-mAID-AU/Hyg.

BCL-XL in pSFFV-neo was a gift from Stanley Korsmeyer; obtained from Addgene; #8749. The BCL-XL cDNA was amplified with PCR (primers 5′‑TTGAATTCATGTCTCAGAGCAA‑3′ and 5′‑GTGAATTCTCATTTCCGACTGA‑3′); the PCR product was then cut with EcoRI and ligated into pUHD-P3 [34] to obtain FLAG-BCL-XL in pUHD-P3. To generate a CRISPR-resistant BCL-XL, the primers 5′‑GAACTGCGCTATCGACGAGCATTC‑3′ and 5′‑GAATGCTCGTCGATAGCGCAGTTC‑3′ were used to introduce silent mutations by double PCR using FLAG-BCL-XL in pUHD-P3 as a template. In the first PCR, BCL-XL was amplified with primers (5′‑TTGAATTCATGTCTCAGAGCAA‑3′; and 5′‑GAATGCTCGTCGATAGCGCAGTTC‑3′); (5′‑GAACTGCGCTATCGACGAGCATTC‑3′ and 5′‑GTGAATTCTCATTTCCGACTGA‑3′). These PCR products were then amplified using the flanking primers (first and last primers) in the second PCR; the PCR products were cut with EcoRI and ligated into EcoRI-cut pUHD-SB-mAID/Hyg with different AU repeats or pUHD-SB-mAID/Bsd to generate mAID-BCL-XL in pUHD-SB-mAID-AU/Hyg or mAID-BCL-XL in pUHD-SB-mAID/Bsd, respectively. mAID-BCL-W in pUHD-SB-mAID/Hyg was generated by ligating NcoI-EcoRI fragment from FLAG-BCL-W in pUHD-P3T [21] into NcoI-EcoRI-cut pUHD-SB-mAID/Hyg [27].

mAID-H6 in pET21d was generated by ligating the NcoI-XhoI-cut PCR product (template: pUHD-SB-mAID/Hyg [27]; primers: 5′-TTTAAGAAGGAGATATACCATGGCCGGAGCA-3′ and 5′-GGCACTCGAGCTTATACATCCT-3′) into NcoI-XhoI cut pET21d (Novagen, Madison, WI, USA).

### Expression of mAID-H6 in bacteria

mAID-H6 in pET21d was transformed into BL21(DE3). The cells were grown at 37 °C until OD_600_ reached 0.4–0.8. The synthesis of mAID-H6 was induced with 1 mM of IPTG. After 3 h at 37 °C, 700 µl of cells were collected by centrifugation and washed three times with PBS (1 ml each). The cells were resuspended in 500 µl of PBS and sonicated, before 500 µl of 6× sample buffer was added. The samples were boiled for 5 min before subjected to SDS-PAGE.

### RNA interference

Stealth siRNA targeting BCL-XL (GCCUUCCAAGGAUGGGUUUTT) and control siRNA were manufactured by RiboBio (Guangzhou, China). Cells were transfected with siRNAs (10 nM) using Lipofectamine RNAiMAX (Thermo Fisher Scientific, Waltham, MA, USA) according to the manufacturer’s instructions.

### Cell lines

HeLa (cervical carcinoma) used in this study was a clone expressing the tTA tetracycline transactivator [35]. KO cell lines were established by transfecting cells with specific CRISPR-Cas9 plasmids and a plasmid expressing blasticidin-resistant gene (a gift from Tim Hunt, Cancer Research UK). Transfected cells were enriched by culturing cells with blasticidin-containing medium for 36 h before seeded onto 10-cm plates (for isolation of mixed population).

The BCL-XL^KO^ expressing ^mAID^BCL-XL were generated by co-transfecting HeLa cells with BCL-XL CRISPR-Cas9 in pX330, mAID-BCL-XL in pUHD-SB-mAID-AU/Hyg (with different numbers of AU), pSBbi-TIR/Pur [27], and Sleeping Beauty transposase pCMV(CAT)T7-SB100 (a gift from Zsuzsanna Izsvak; Addgene, #34879). Transfected cells were enriched by culturing in hygromycin- and puromycin-containing medium for 14 days. Single cell-derived colonies were obtained by limiting dilution in 96-well plates. KO cell lines of BCL2, BAK, or BAX in ^mAID^BCL-XL^KO^ cells were established as described above. The mixed-population of ^mAID^BCL-XL^KO^ BAX^KO^ was then transfected with BAK CRISPR-Cas9 in pX330 to generate BAX and BAK double-KO in ^mAID^BCL-XL^KO^ cells. ^mAID^BCL2^KO^ cells were generated similarly as ^mAID^BCL-XL^KO^ except that CRISPR-Cas9 and mAID-tagged construct (mAID-BCL2 in pUHD-SB-mAID-AU/Hyg) specific for BCL2 were used. ^mAID^BCL2^KO^ cells lacking BCL-XL were generated by transfection of BCL-XL CRISPR-Cas9 in pX330, as described above. ^mAID^MCL1^KO^ cells were established as previously described [10]. HeLa cells expressing FLAG-tagged BCL2 or BCL-XL were generated as previously described [21].

RPE1 (hTERT-immortalized retinal pigment epithelial) cells were obtained from American Type Culture Collection (Manassas, VA, USA). The BCL-XL^KO^ expressing ^mAID^BCL-XL were generated by co-transfecting BCL-XL CRISPR-Cas9 in pX330, mAID-BCL-XL in pUHD-SB-mAID/Bsd, pSBbi-TIR1-tTA/Neo [27], and Sleeping Beauty transposase pCMV(CAT)T7-SB100 (a gift from Zsuzsanna Izsvak; Addgene, #34879). Transfected cells were enriched by culturing in blasticidin- and G418-containing medium for 14 days. Single cell-derived colonies were isolated by limiting dilution in 96-well plates.

### Cell culture and synchronization

HeLa cells were propagated in Dulbecco’s modified Eagle’s medium (DMEM) supplemented with 10% (v/v) calf serum and 50 U/ml of penicillin streptomycin (Thermo Fisher Scientific). RPE1 cells were propagated in DMEM-F12 medium supplemented with 10% (v/v) fetal bovine serum, 50 U/ml of penicillin-streptomycin, and 10 mg/ml of hygromycin B. Cells were cultured in humidified incubators at 37 °C with 5% CO_2_.

Unless stated otherwise, cells were treated with the following reagents at the indicated final concentrations: ABT-199 (MedChemExpress, NJ, USA; 10 μM), blasticidin (Thermo Fisher Scientific; 3.75 µg/ml for transient selection; 2.5 µg/ml for stable selection; 7.5 µg/ml for RPE1 cells), cycloheximide (CHX) (Sigma-Aldrich, St. Louis, MO, USA; 10 μg/ml), doxycycline (Dox) (Sigma-Aldrich; 2 µg/ml), G418 (Santa Cruz Biotechnology, Santa Cruz, CA, USA; 0.75 mg/ml), hygromycin B (Thermo Fisher Scientific; 0.25 mg/ml), indole-3-acetic acid (IAA) (Sigma-Aldrich; 50 µg/ml), nocodazole (NOC) (Sigma-Aldrich; 100 ng/ml), puromycin (Sigma-Aldrich; 0.75 µg/ml for transient selection; 0.3 µg/ml for stable selection), RO3306 (Santa Cruz Biotechnology; 10 μM), and thymidine (Santa Cruz Biotechnology; 2 mM). HeLa cells were transfected with plasmids using a calcium phosphate precipitation method [36]. RPE1 cells were transfected using Lipofectamine 3000 (Thermo Fisher Scientific) according to the manufacturer’s instruction. Unless stated otherwise, transfected cells were grown for 24 h before harvested for further analysis.

Synchronization using double thymidine and NOC shake-off were as previously described [10]. Briefly, the cells were washed with PBS at 8 h after release from the second thymidine block. A portion of attached cells were harvested as G_2_ samples while the remaining attached cells were incubated with NOC for an additional 4 h. Mitotic cells were isolated with mechanical shake-off and were either harvested immediately or allowed to incubate in the NOC-containing medium for different time points. Both attached and floating cells were harvested for preparation of cell-free extracts [37]. For siRNA experiments, transfection of siRNA was performed 6 h before the start of synchronization.

### Live-cell imaging

Cells were seeded onto 24-well cell culture plates and placed into an automated microscopy system equipped with temperature, humidity, and CO_2_ control chamber (Zeiss Celldiscoverer 7, Oberkochen, Germany). Images were captured every 5 min for up to 24 h. Data acquisition was carried out using Zeiss ZEN 2.3 (blue edition), and subsequent analysis was performed using ImageJ (National Institutes of Health, Bethesda, MD, USA). Apoptosis was determined based on morphological changes. After mitosis, one of the daughter cells was randomly selected and continued to be tracked. To analyze the duration of mitosis, we assumed that the data followed a normal distribution within a specific treatment group and exhibited a comparable variance across different groups.

### Antibodies and immunological methods

The following antibodies were obtained from the indicated sources: beta-actin (A5316; Sigma-Aldrich), BAK (sc-517390; Santa Cruz Biotechnology), BAX (sc-493; Santa Cruz Biotechnology), BCL2 (15071; Cell Signaling Technology, Beverly, MA, USA), BCL-W (2724 S; Cell Signaling Technology), BCL-XL (A5091; Bimake, Houston, TX, USA), cyclin A2 (AT10; a gift from Tim Hunt, Cancer Research UK), cyclin B1 (sc-245; Santa Cruz Biotechnology), phosphorylated histone H3^Ser10^ (sc-8656R; Santa Cruz Biotechnology), GAPDH (sc-32233; Santa Cruz Biotechnology; a gift from XiaoQi Wang, the University of Hong Kong), MCL1 (sc-819; Santa Cruz Biotechnology; 94296 S; Cell Signaling Technology), mini-AID (M214-3; MBL International, Woburn, MA, USA), and cleaved PARP1 (552597; BD Biosciences, Franklin Lakes, NJ, USA). The positions of molecular size standards (in kDa) are indicated in the Figures. Quantification of signals on immunoblotting was conducted with Image Lab software (version 5.2.1 build 11, Bio-Rad Laboratories, Hercules, CA, USA). Uncropped immunoblot images are shown in Supplemental Materials.

### Supplementary information

Uncropped Western blots

Supplemental Figures

## Data Availability

All primary data are available upon request.
