# Supplementary material for: BCL-XL regulates the timing of mitotic apoptosis independently of BCL2 and MCL1 compensation
Source: Cell Death Dis. 2024 Jan 3;15(1):2. doi: 10.1038/s41419-023-06404-9 (PMC10764939; doi:10.1038/s41419-023-06404-9)
Supplement: Supplementary file 1 — Uncropped Western blots [file 41419_2023_6404_MOESM1_ESM.pdf]

Supplemental materials: uncropped Western blots

Boxes indicate the lanes and bands used in the Figures and Supplemental Figures.

Fig 1A

BCL-XL

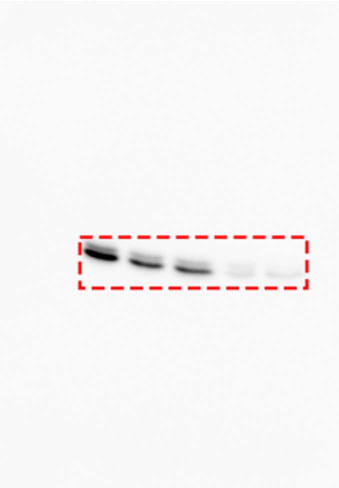

BCL2

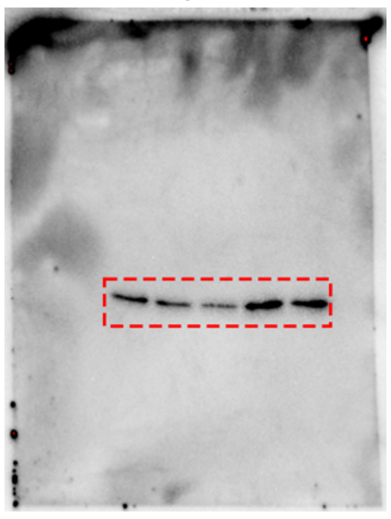

BCL-W

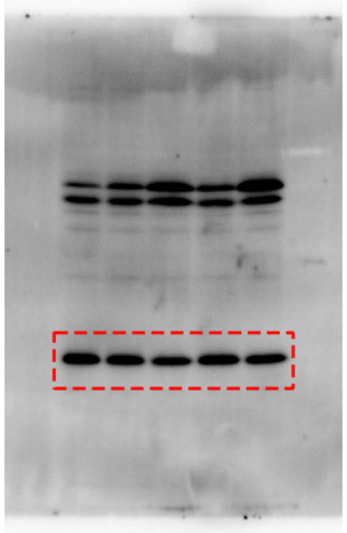

Actin

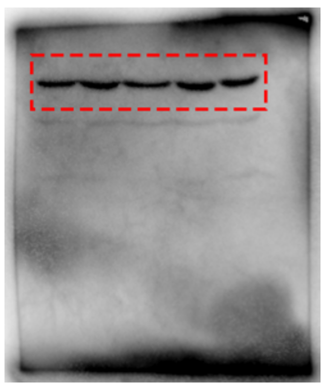

Fig 1B

BCL-XL

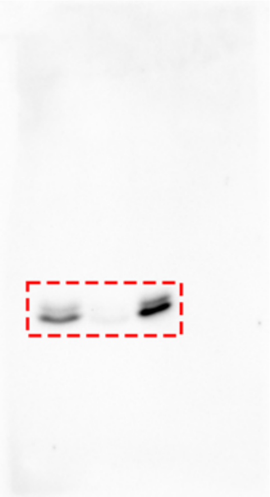

BCL2

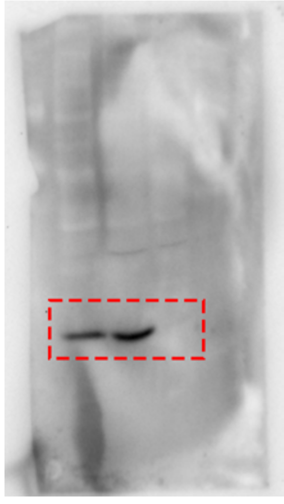

Actin

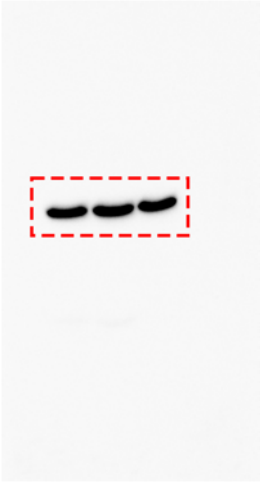

Fig 1C

BCL-XL

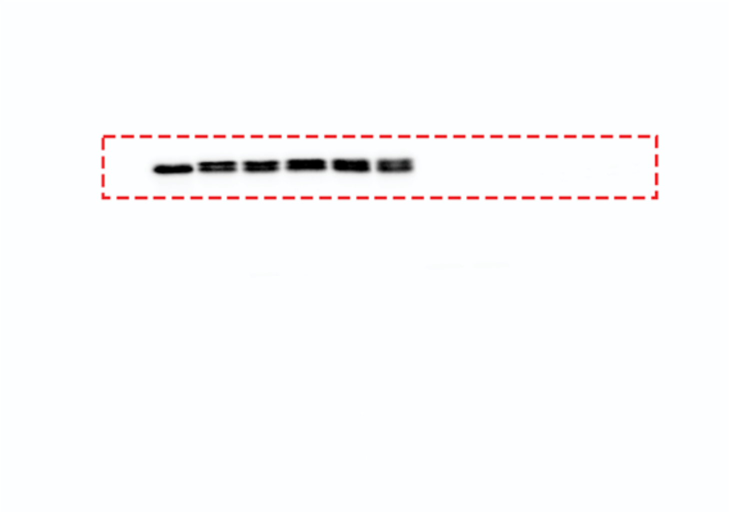

BCL2

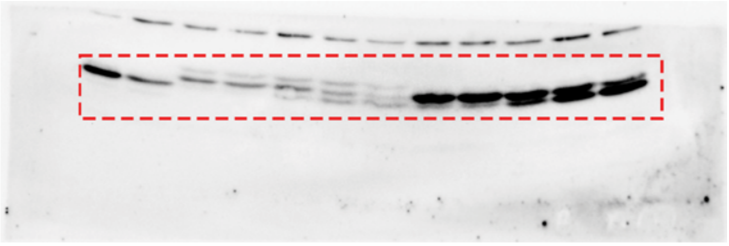

PARP-1
(Cleaved)

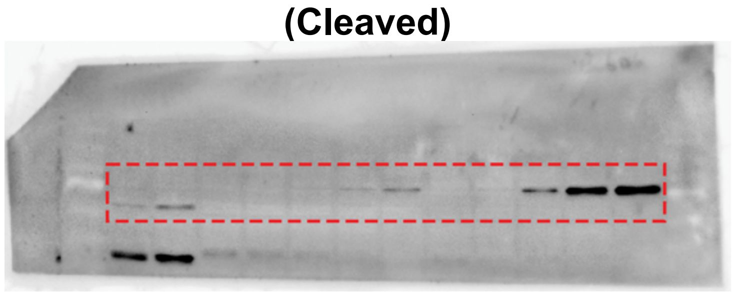

Actin

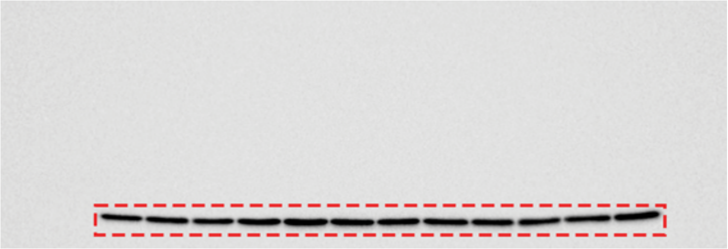

Fig 1D

BCL-XL

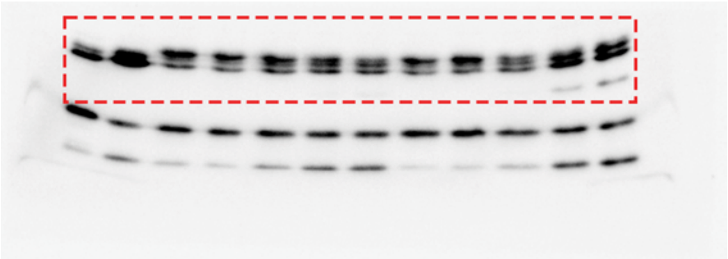

BCL2

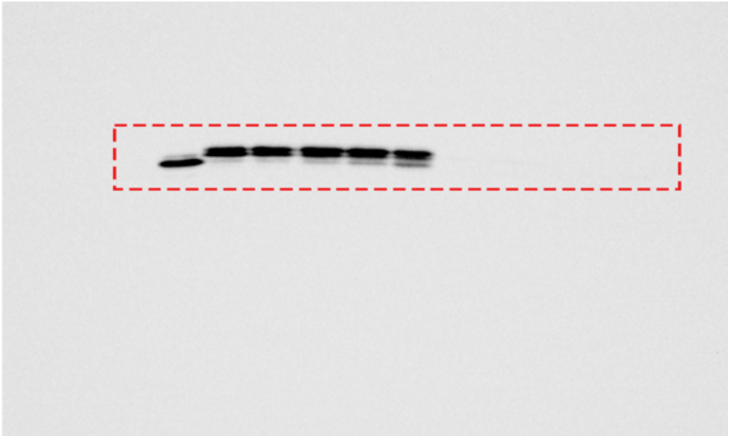

PARP-1
(Cleaved)

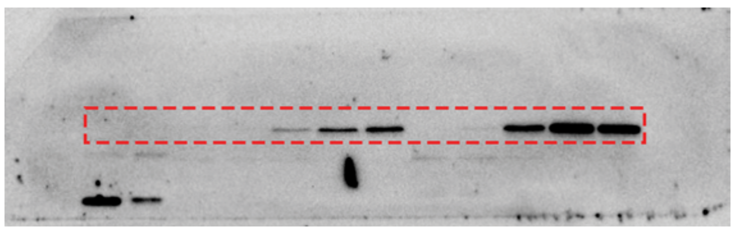

Actin

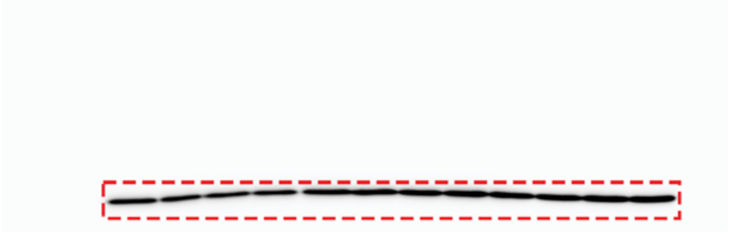

Original Western blots of Figure 1

Fig 2B

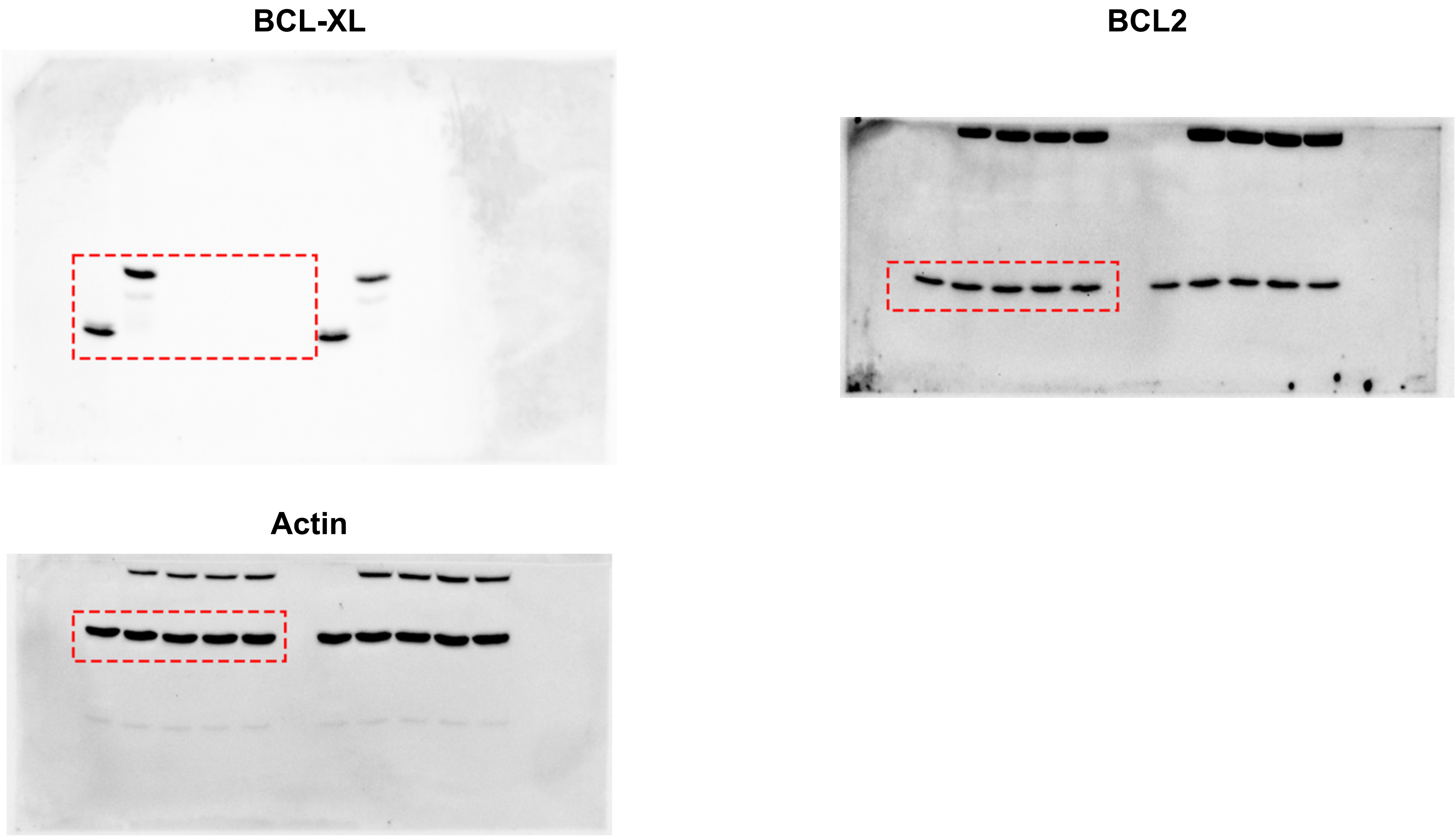

Fig 2C

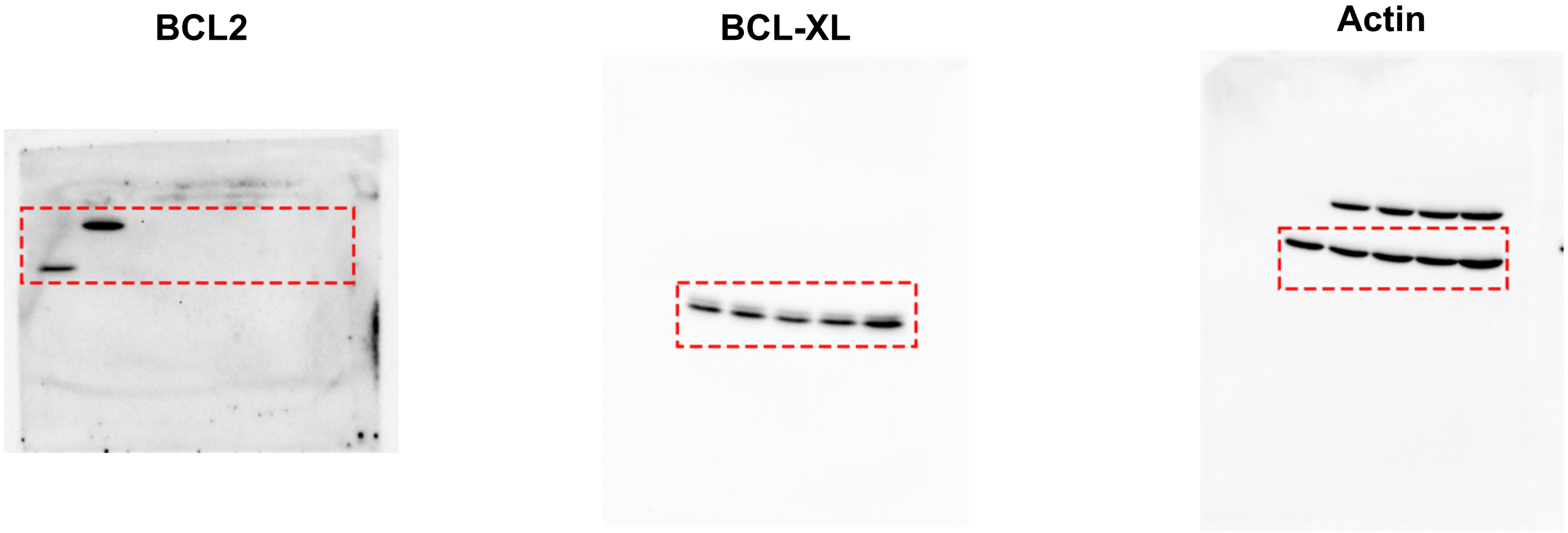

Original Western blots of Figure 2

Fig 3A

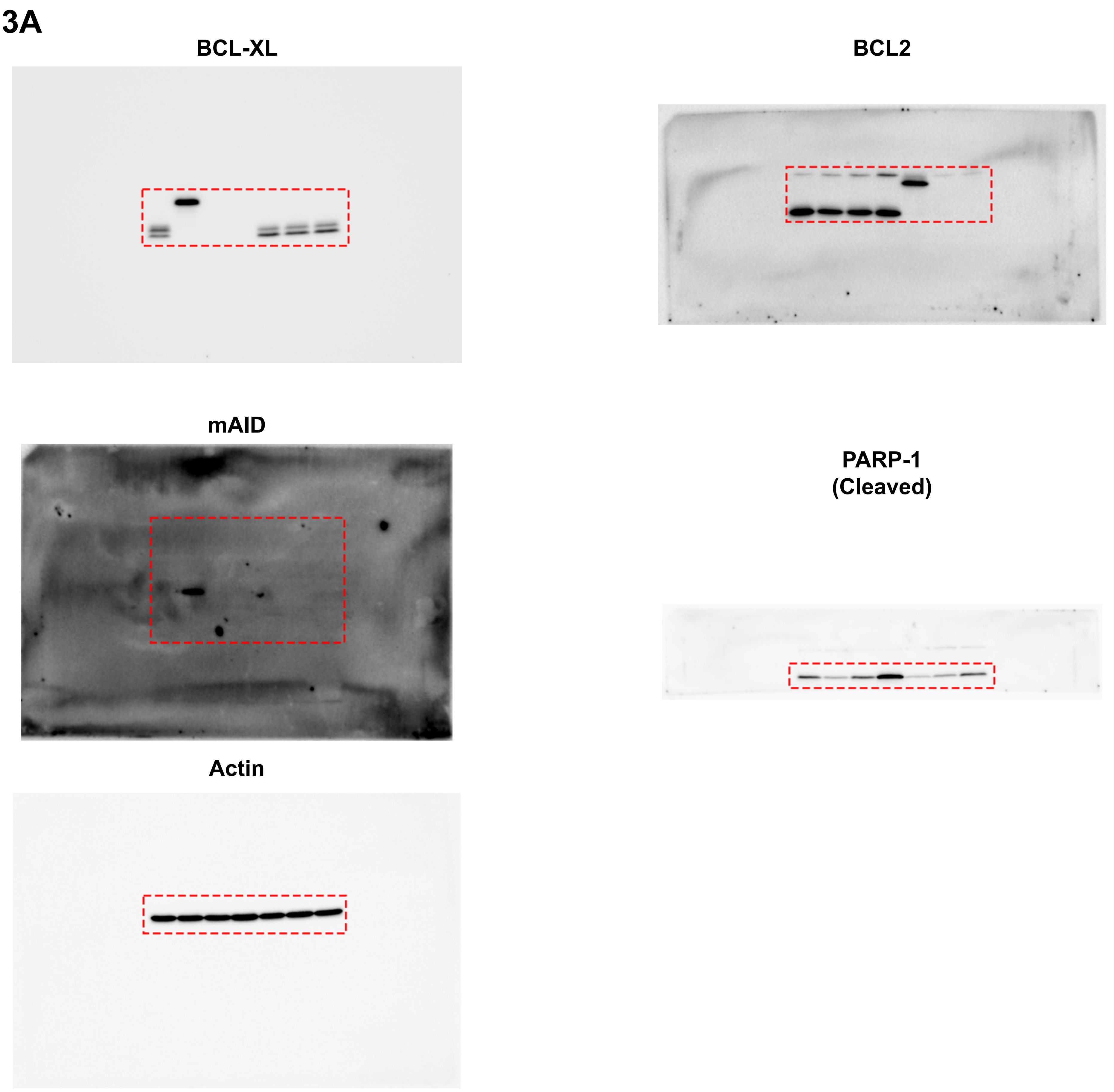

Fig 3B

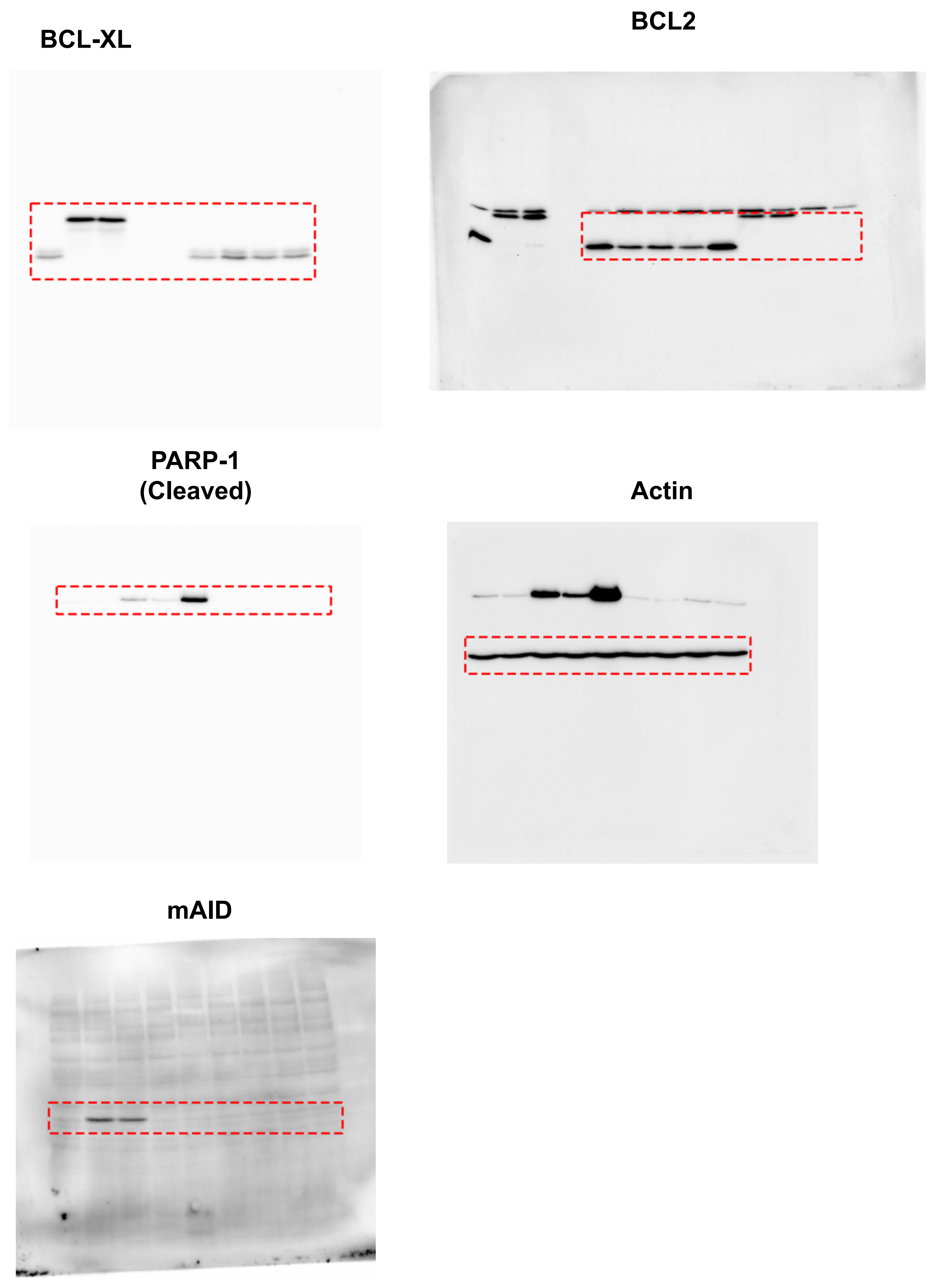

Fig 3C

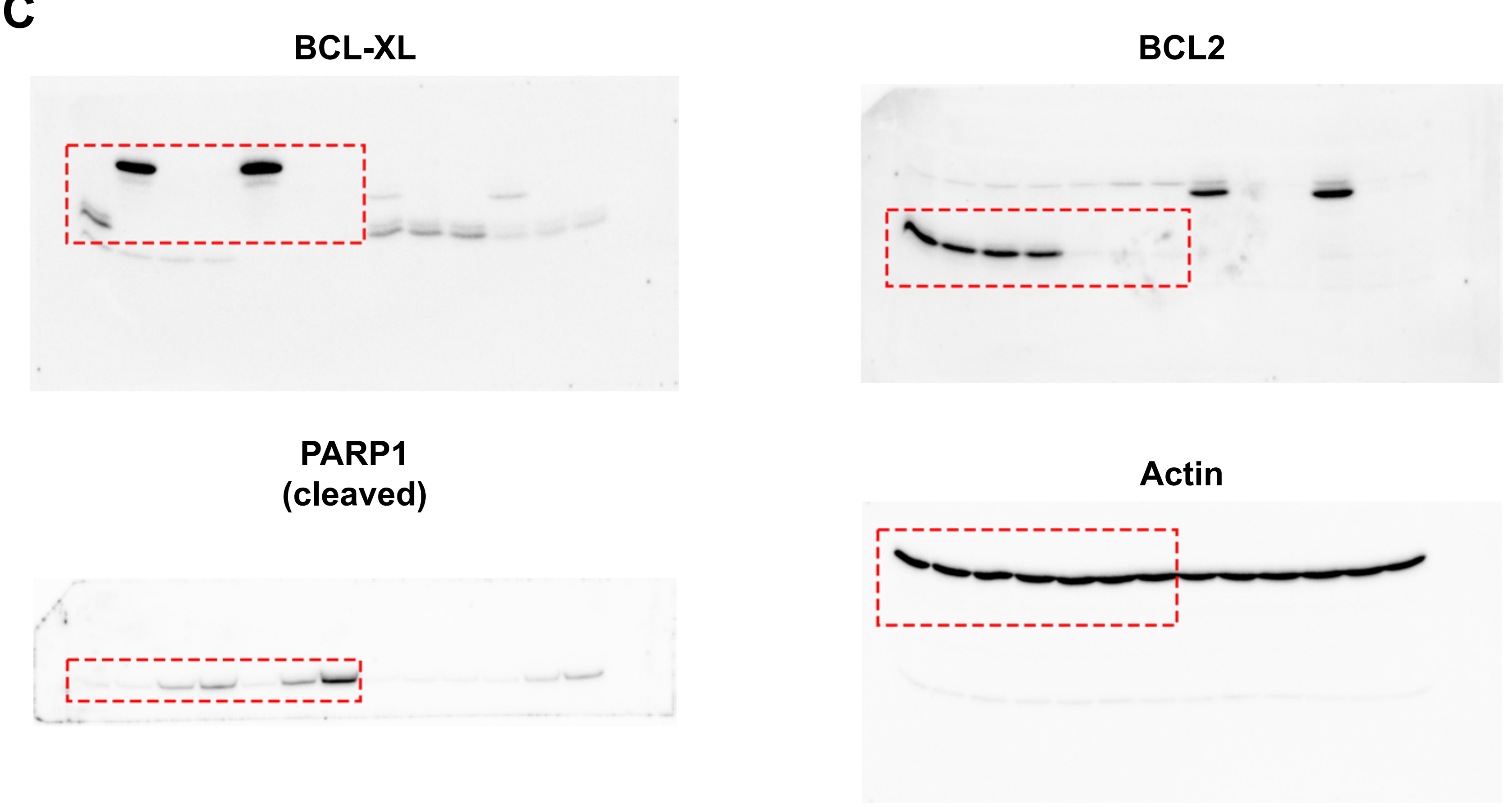

Fig 3D

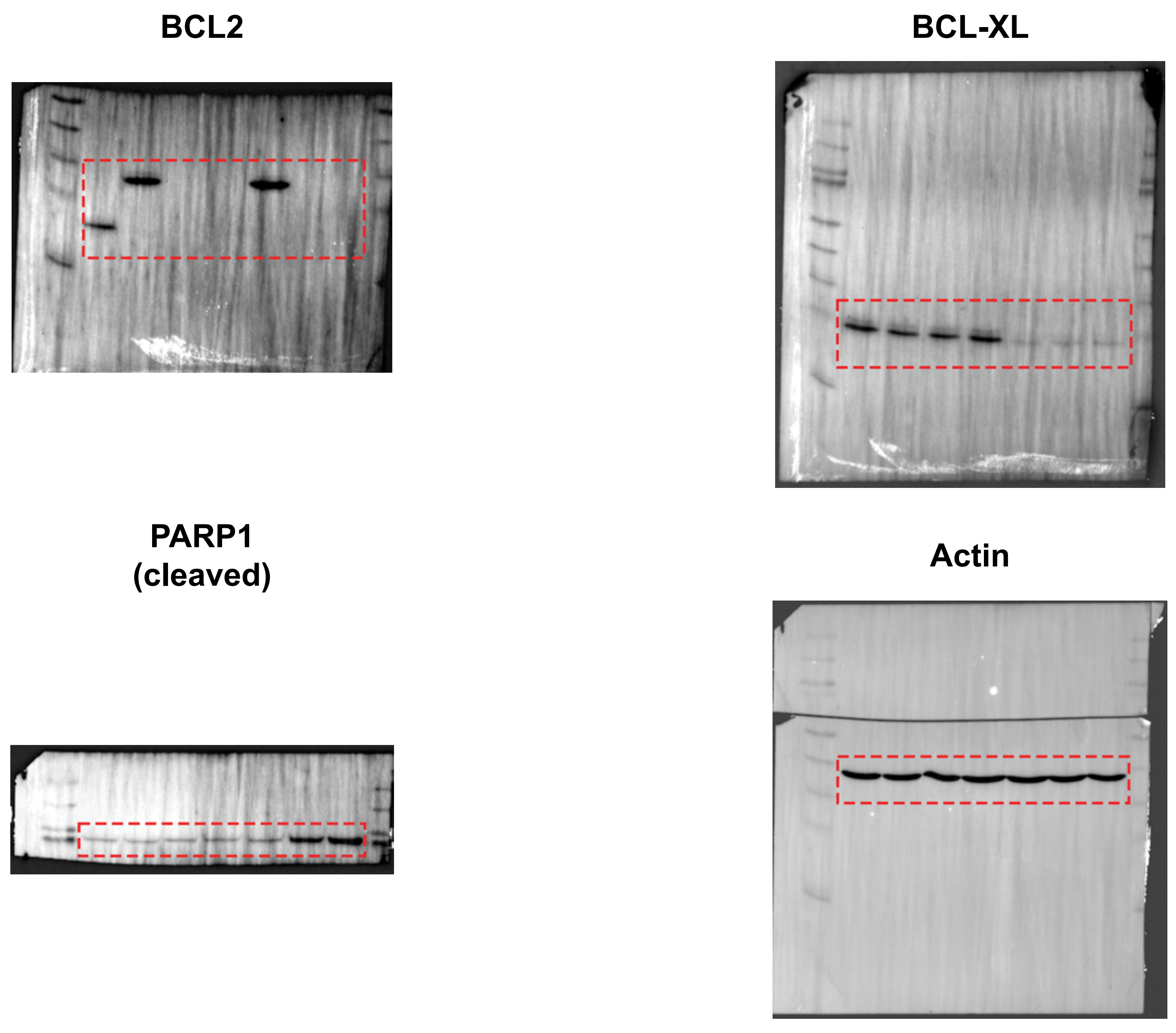

Fig 3E

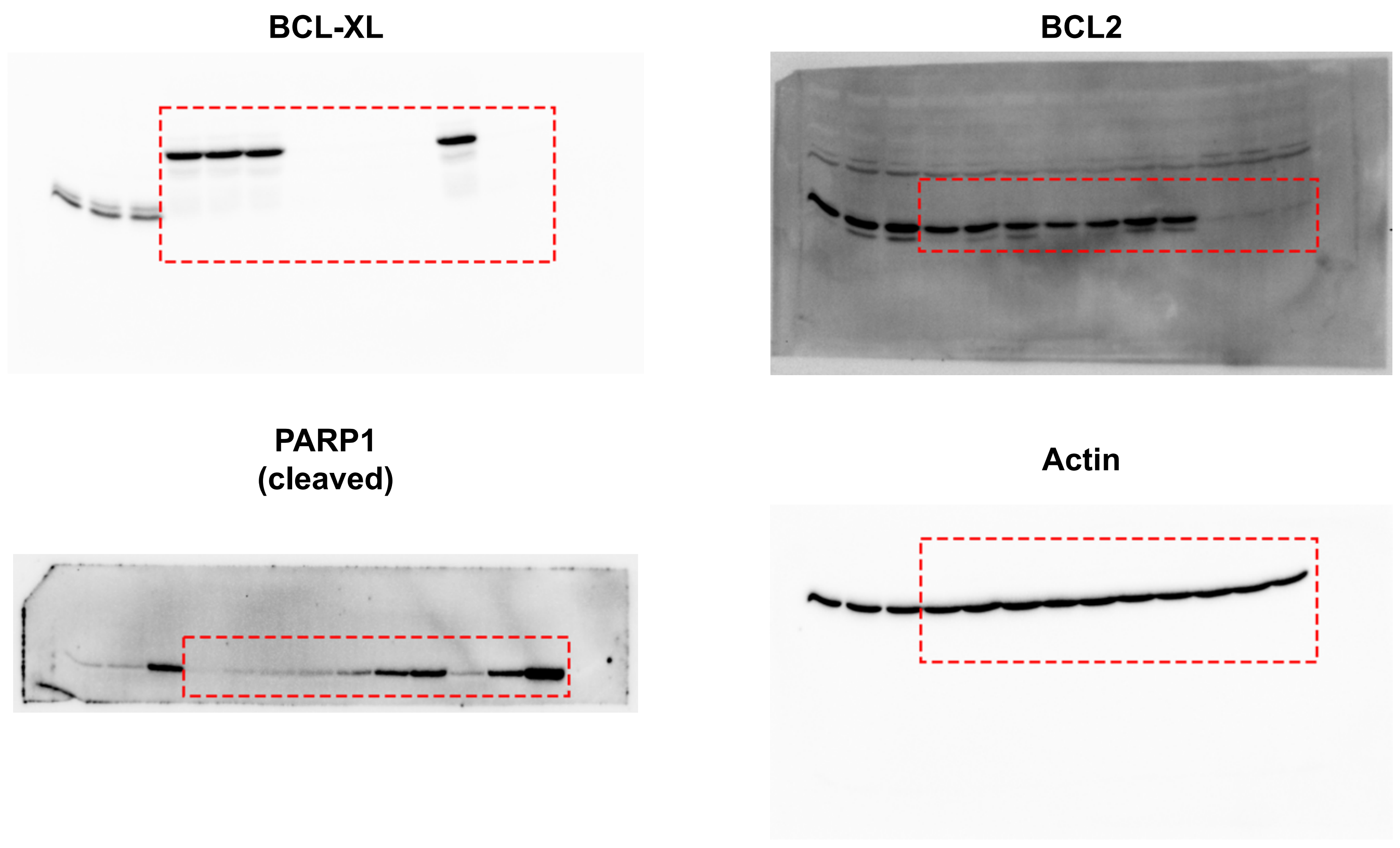

Original Western blots of Figure 3

Fig 4A

BCL2

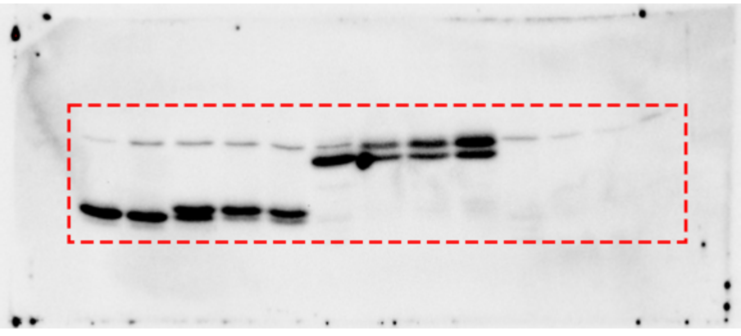

BCL-XL

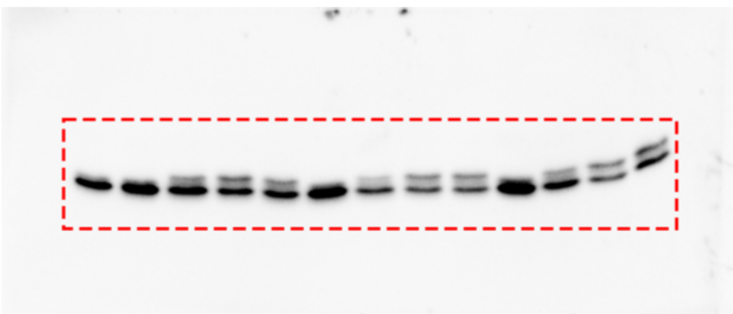

Cyclin A

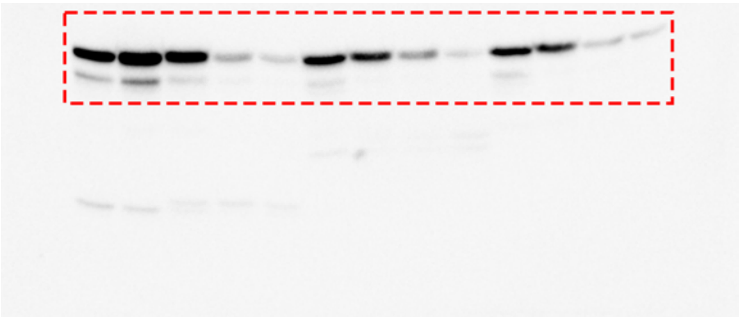

Cyclin B1

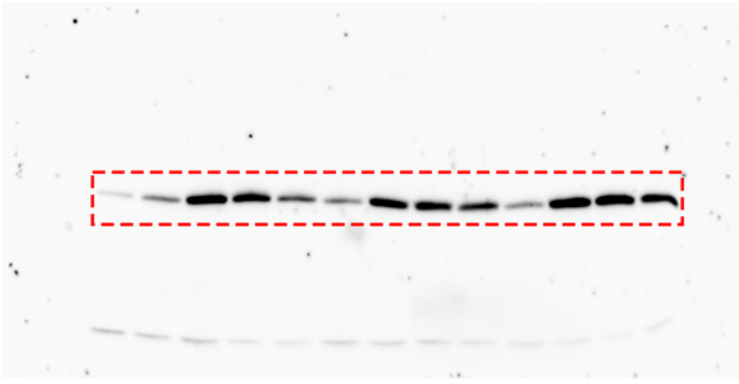

Histone H3
(S10-p)

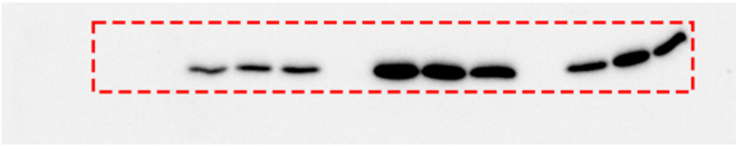

PARP-1
(Cleaved)

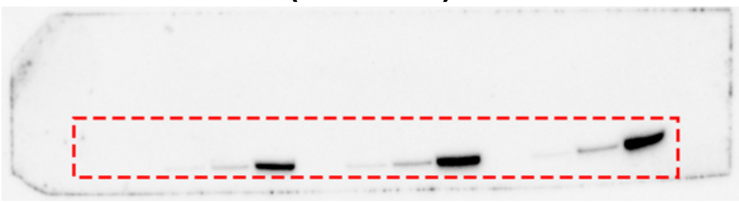

Actin

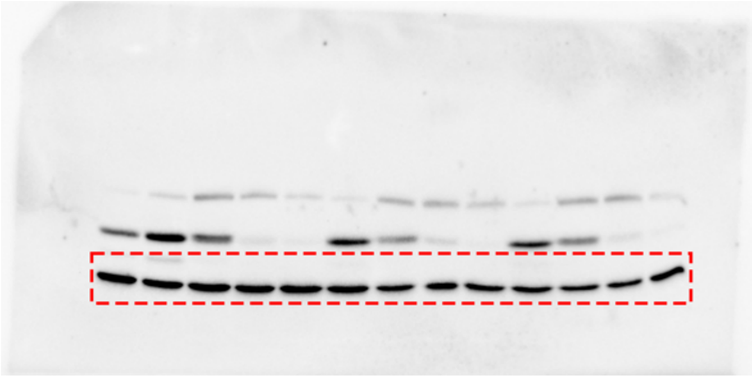

Fig 4B

BCL-XL

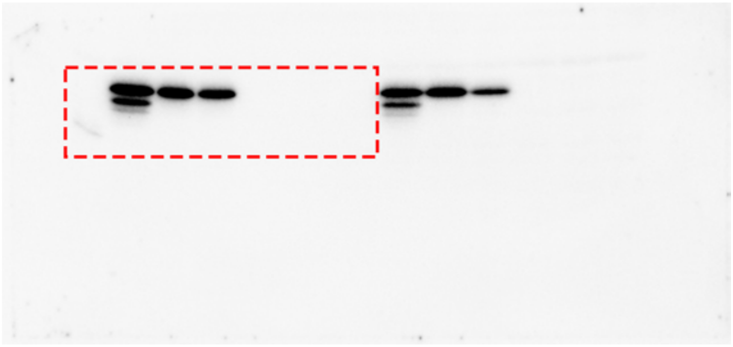

BCL2

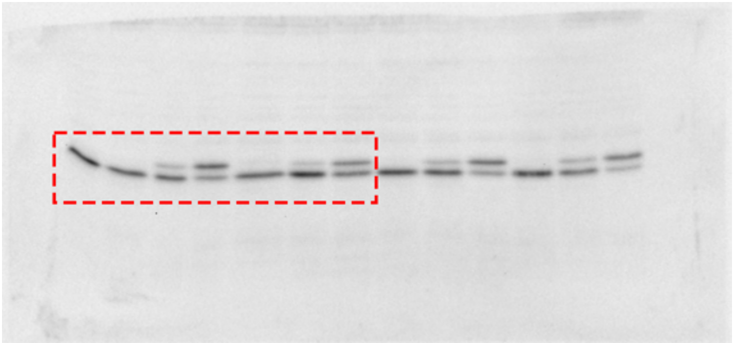

Cyclin B1

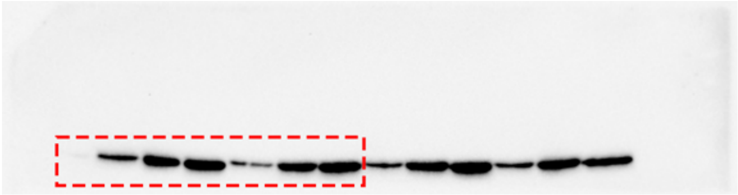

Histone H3
(S10-p)

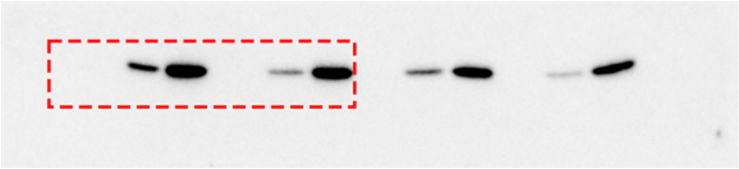

PARP-1
(Cleaved)

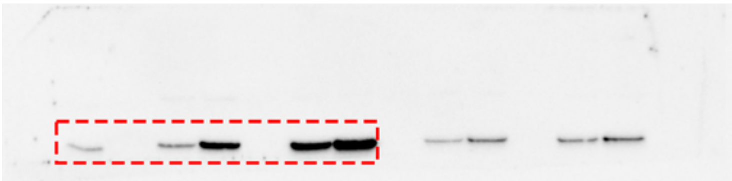

Actin

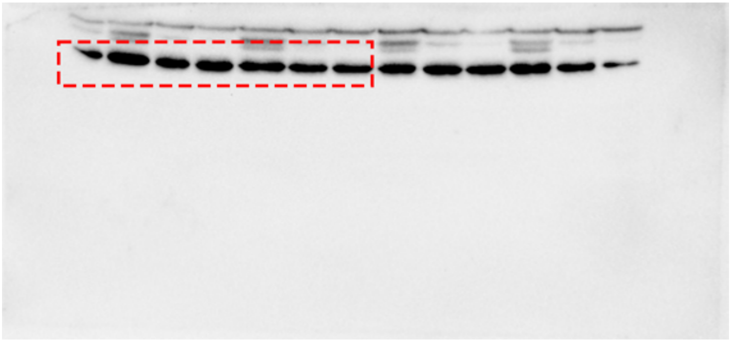

Original Western blots of Figure 4

Fig 5A

BCL-XL

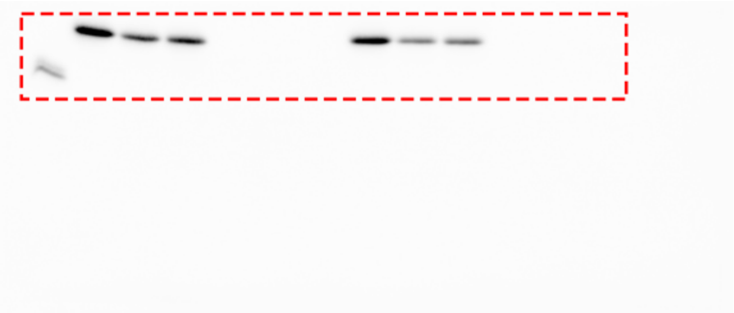

BCL2

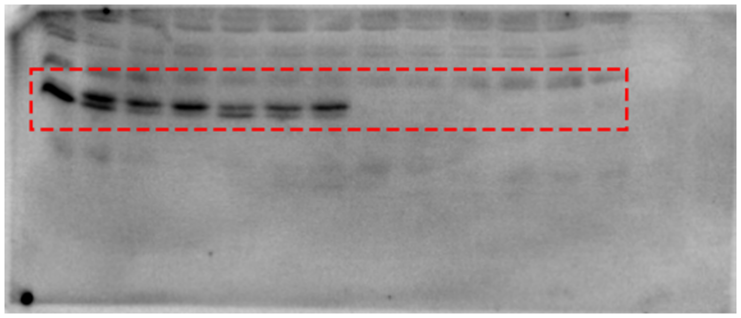

Cyclin A

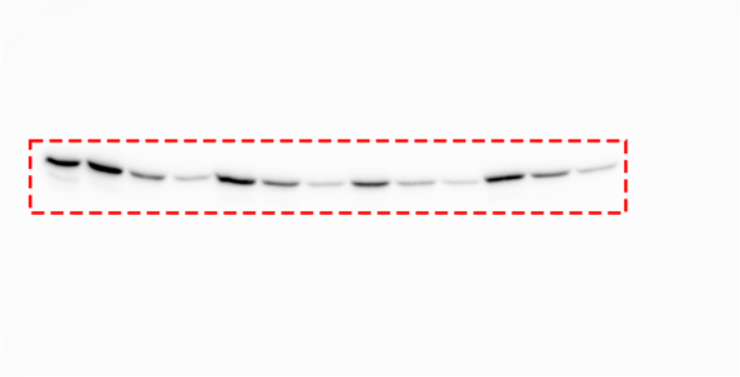

Cyclin B1

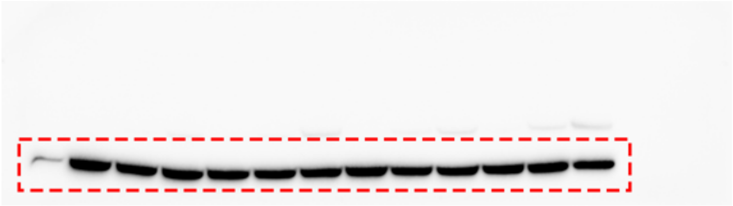

Histone H3 (S10-p)

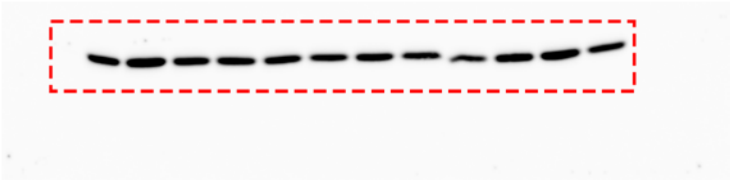

PARP-1 (Cleaved)

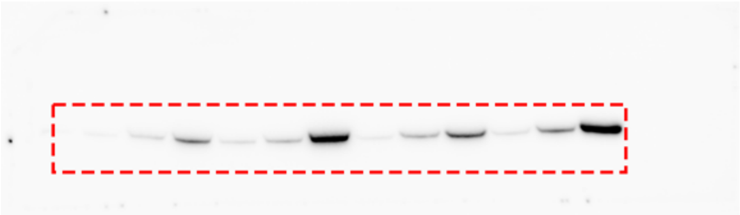

Actin

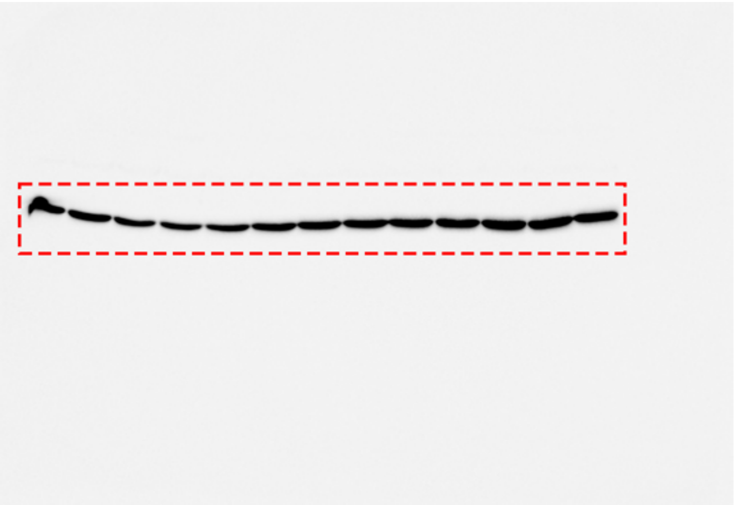

Original Western blots of Figure 5

Fig 6A

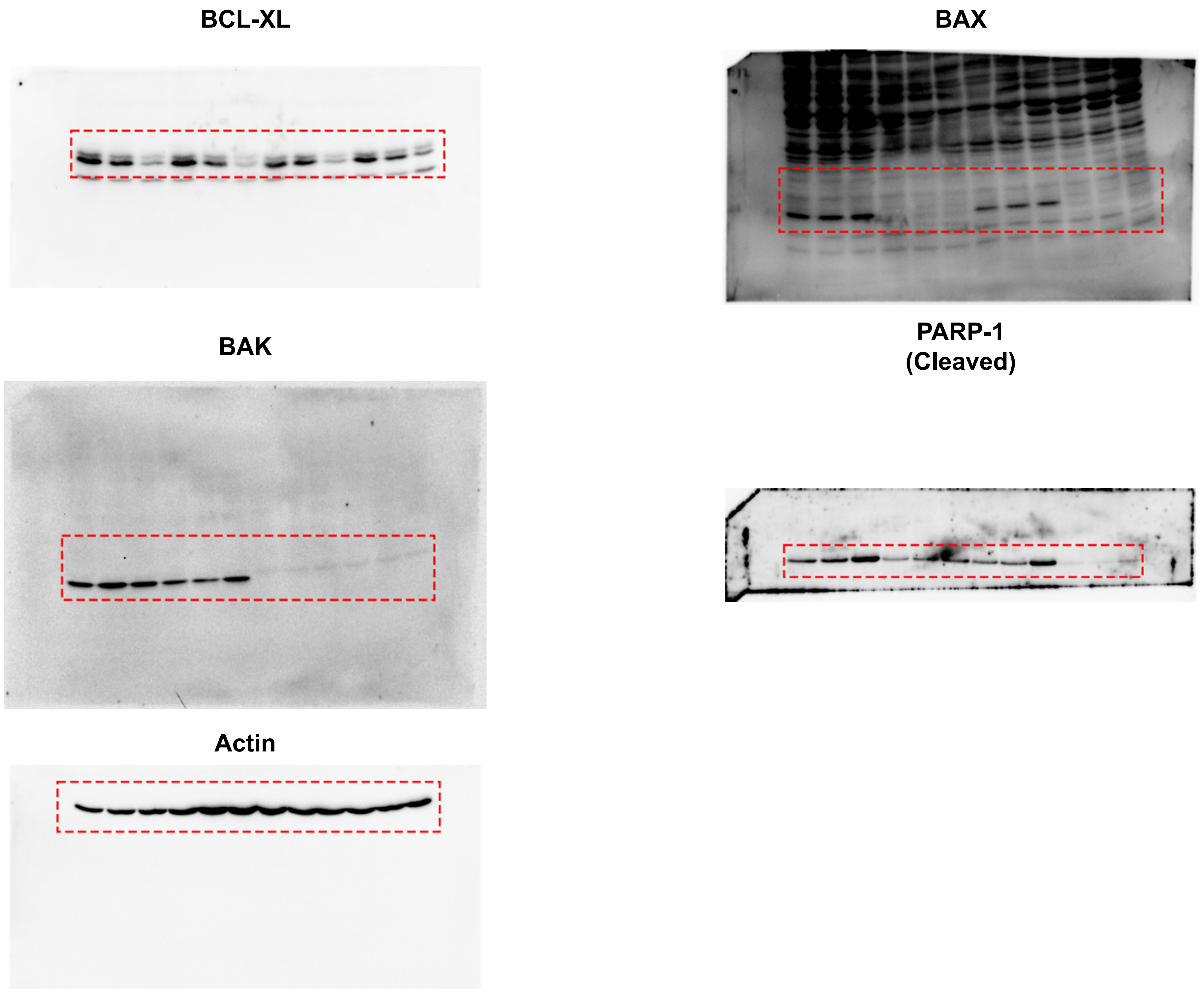

Fig 6B

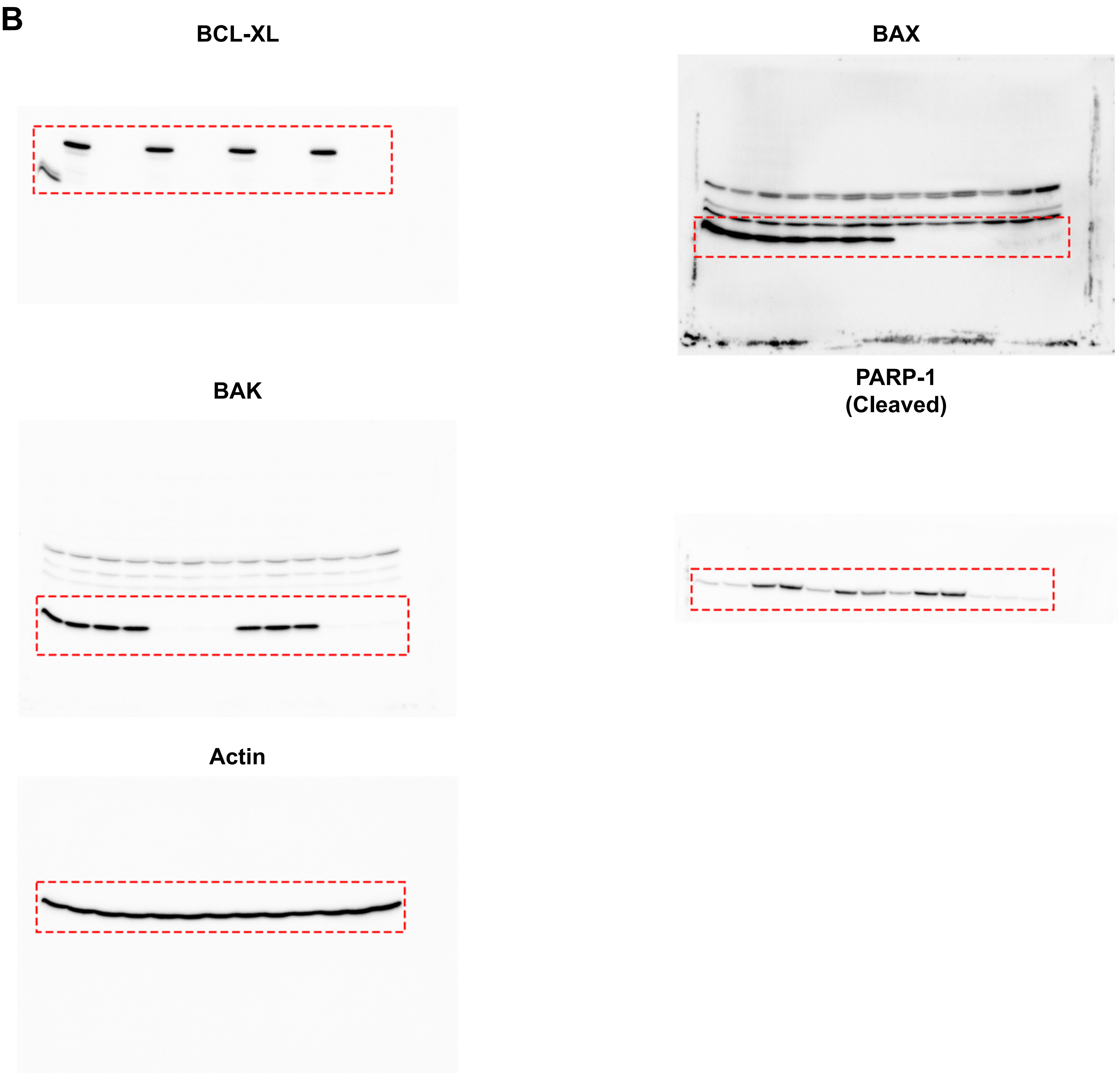

Fig 6C

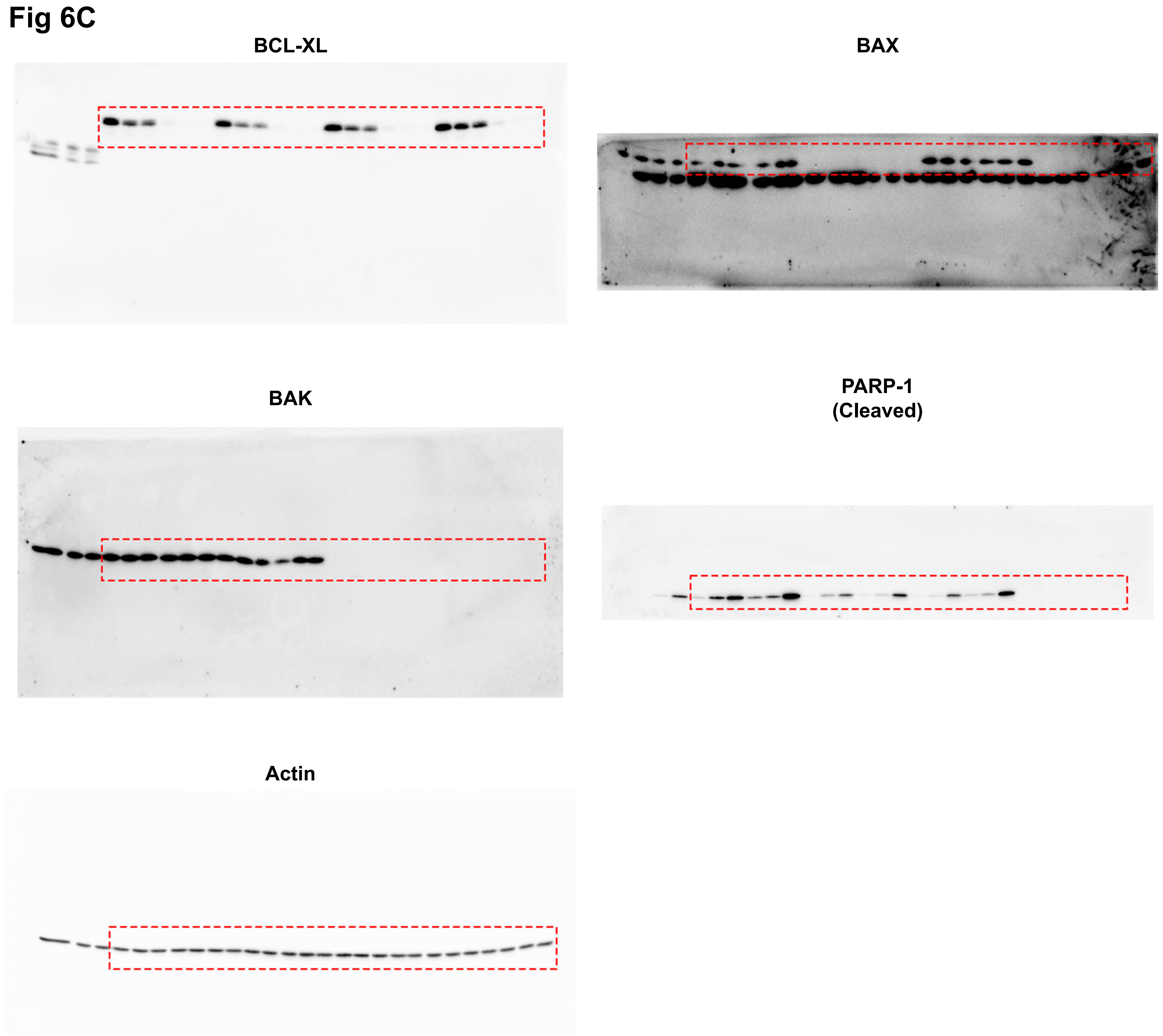

Original Western blots of Figure 6

Fig 7A

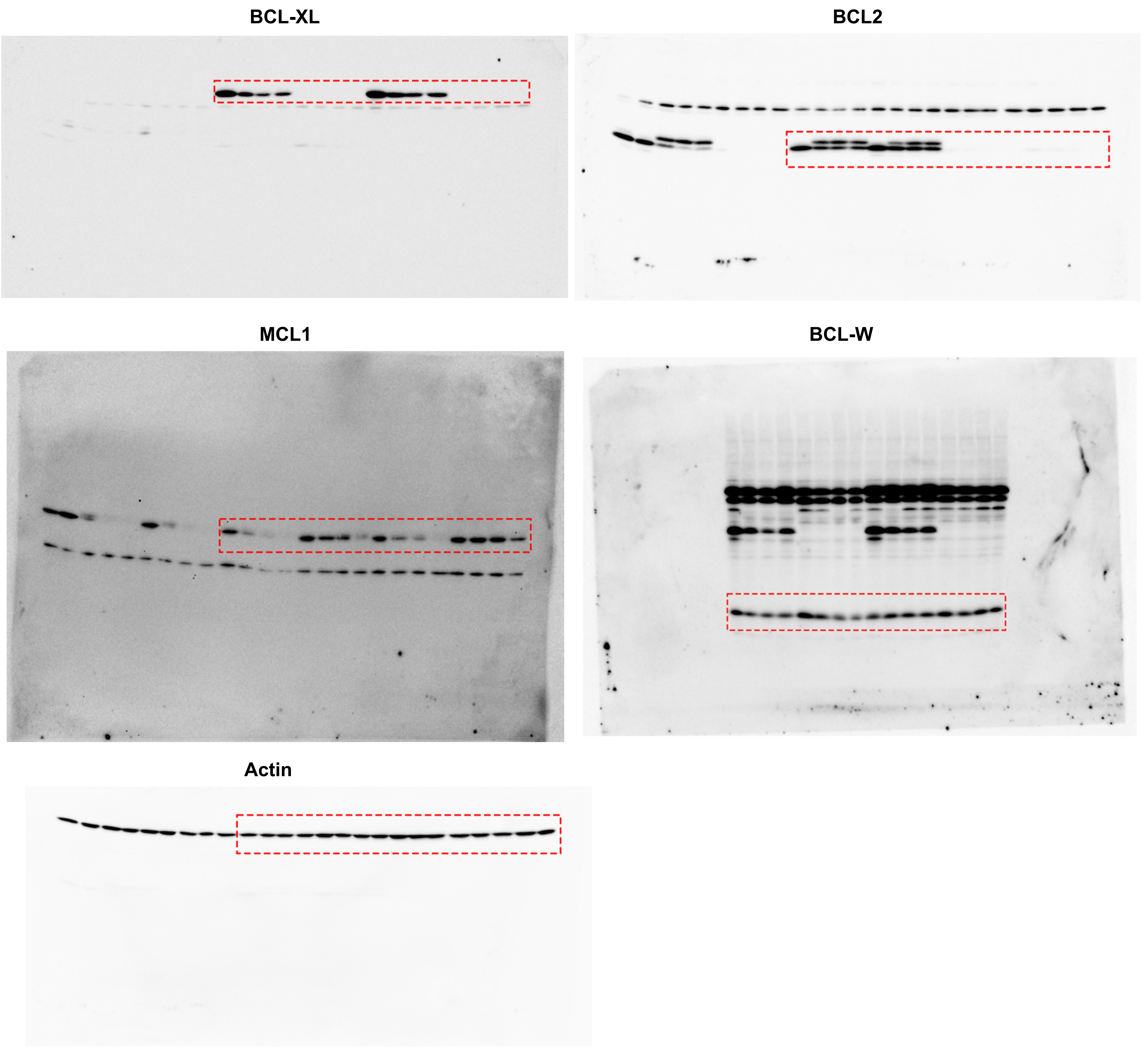

Fig 7B_Left

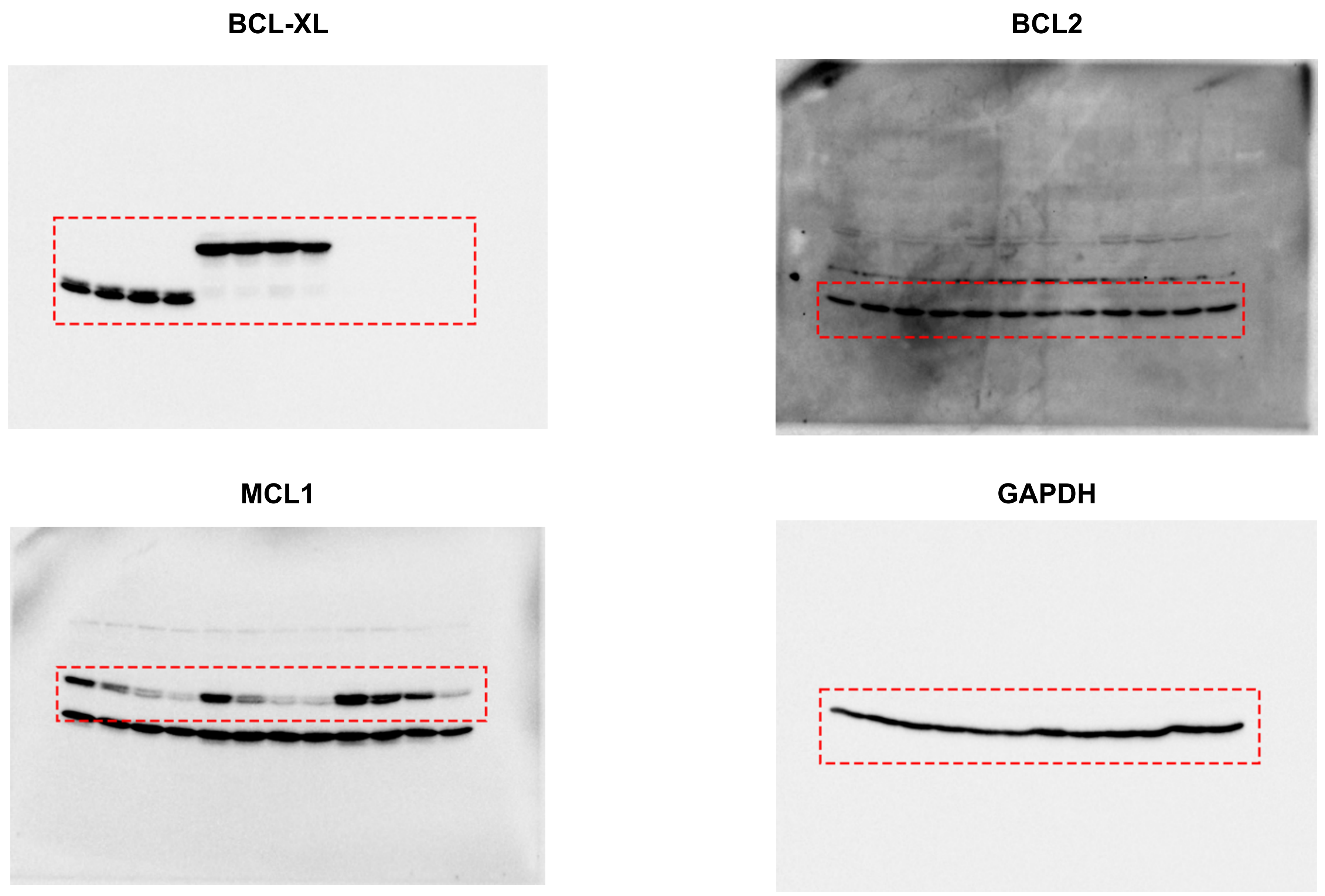

Fig 7B_Right

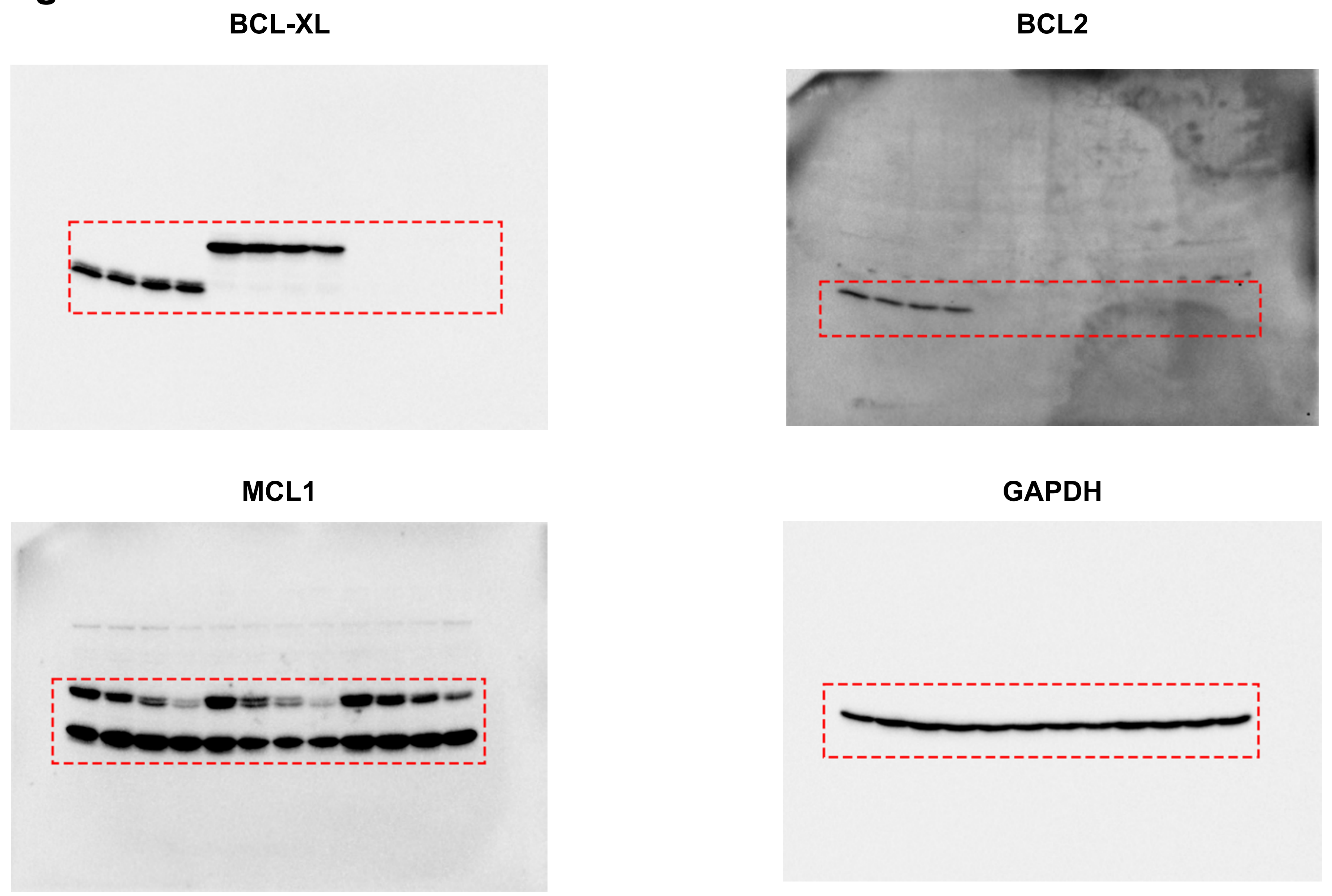

Fig 7C

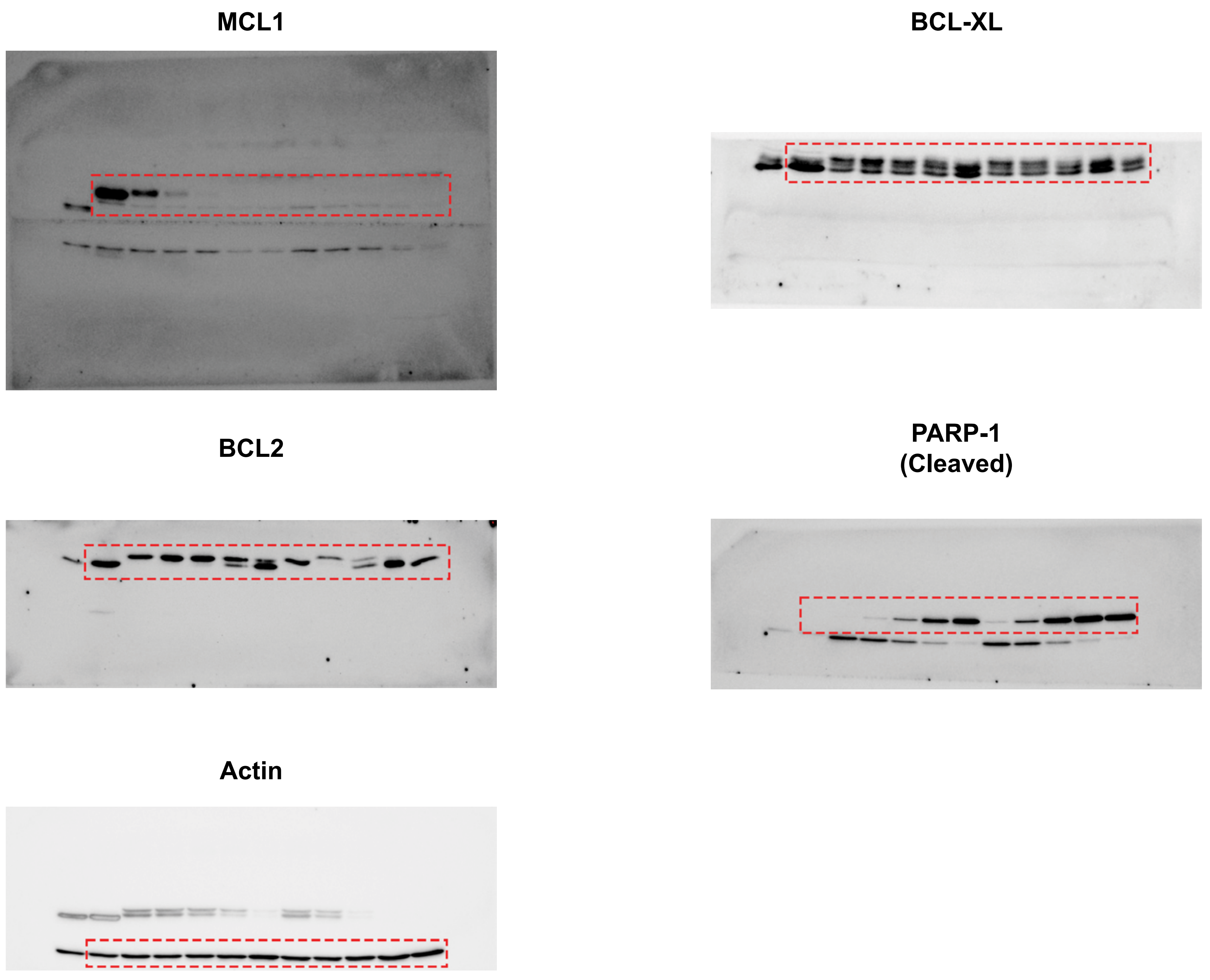

Fig 7D

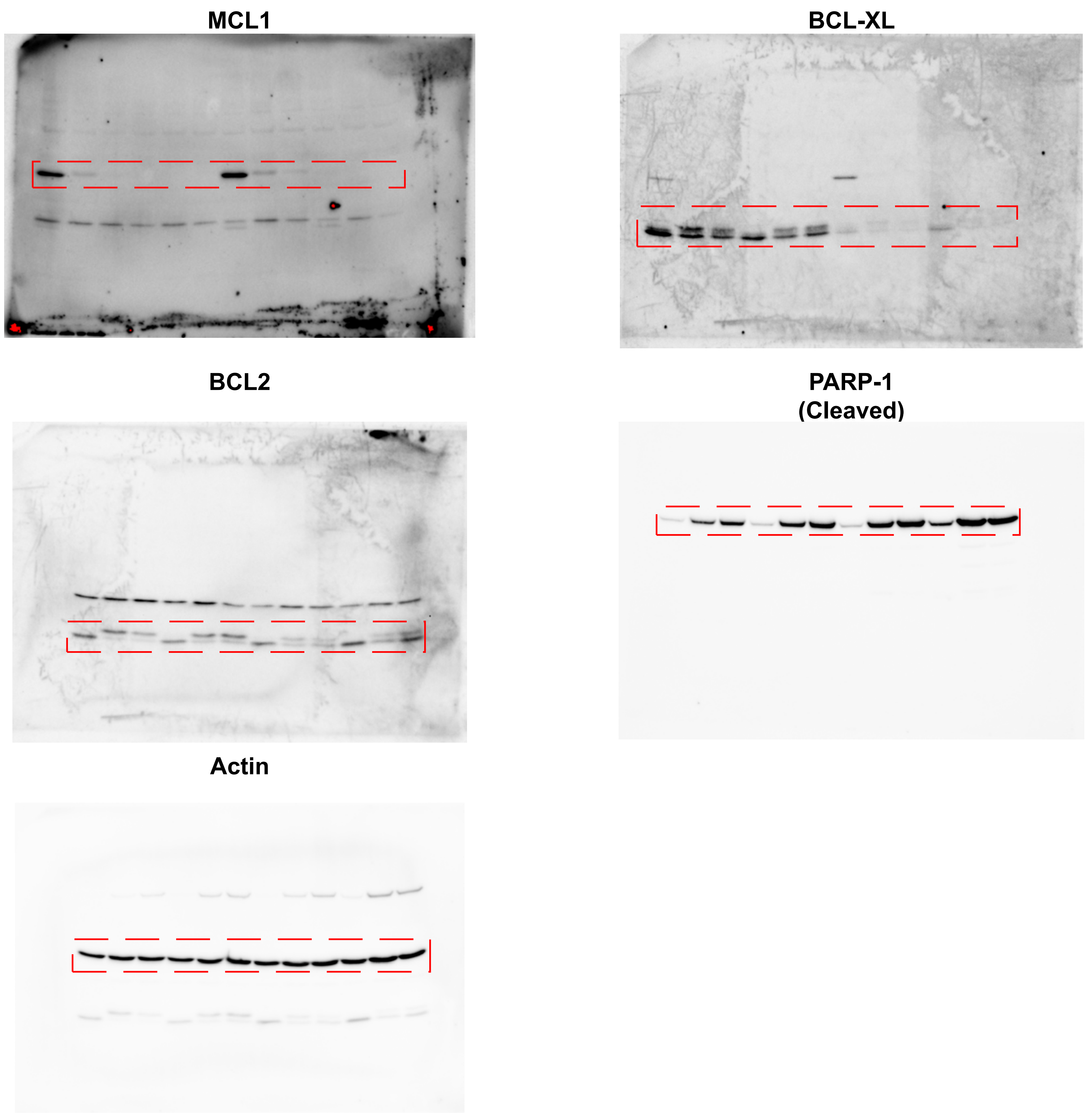

Original Western blots of Figure 7

Fig S1A

BCL-XL

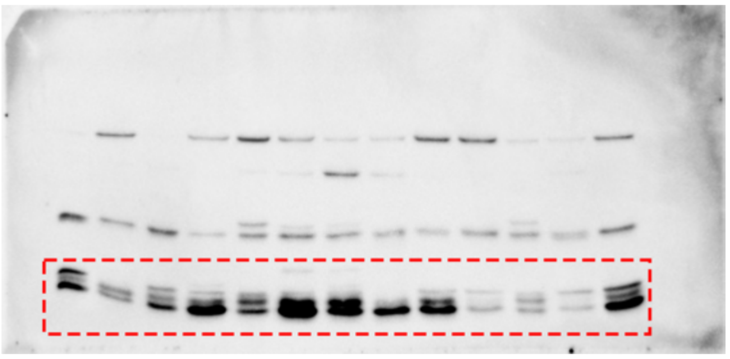

BCL2

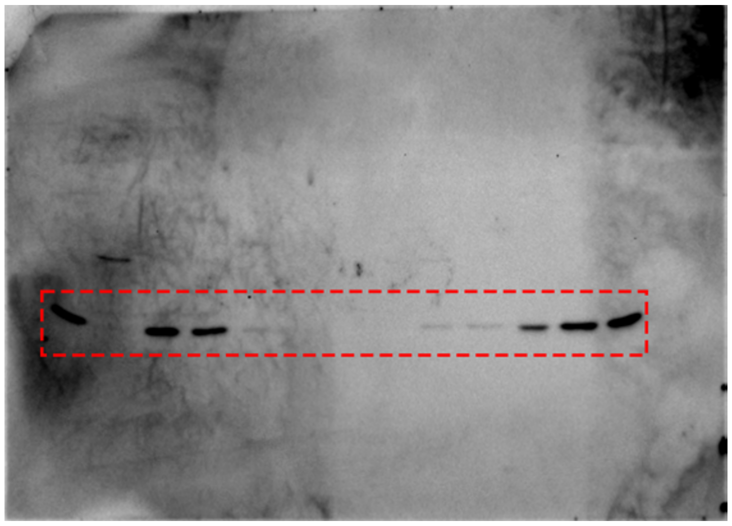

Actin

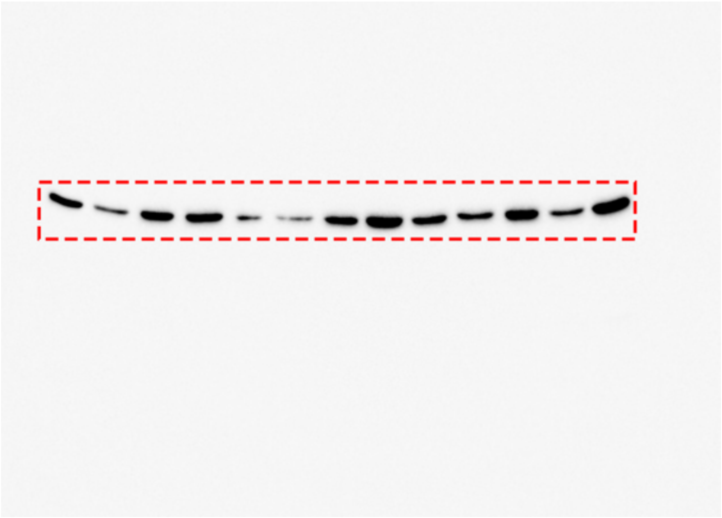

Original Western blots of Figure S1

Fig S2A

BCL-XL

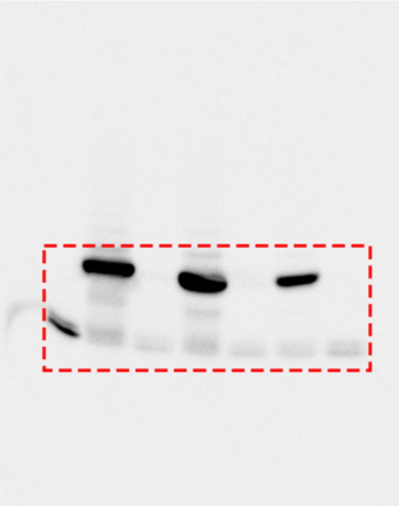

Actin

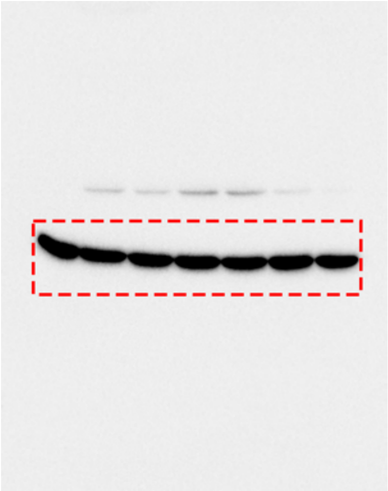

Fig S2B

BCL2

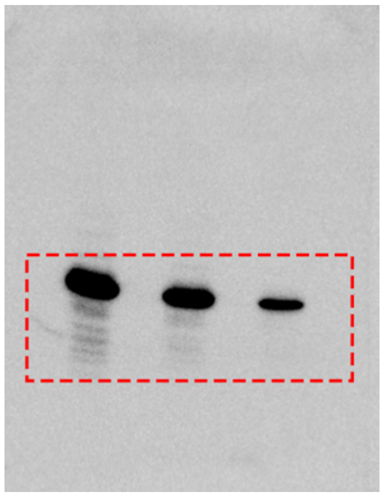

BCL2

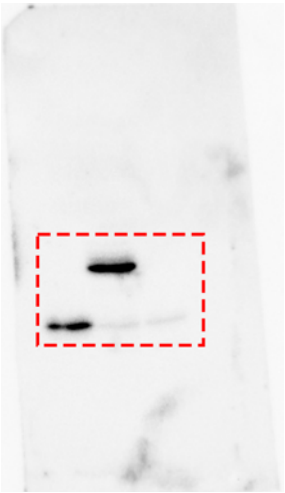

Actin

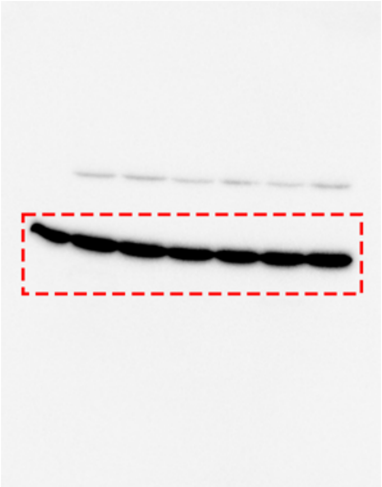

Actin

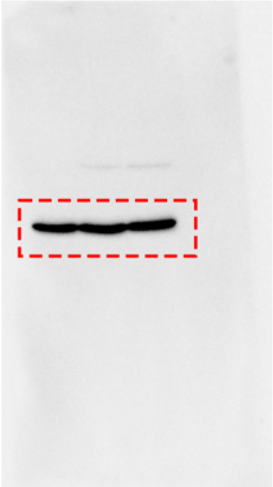

Original Western blots of Figure S2

Fig S3A

BCL-W

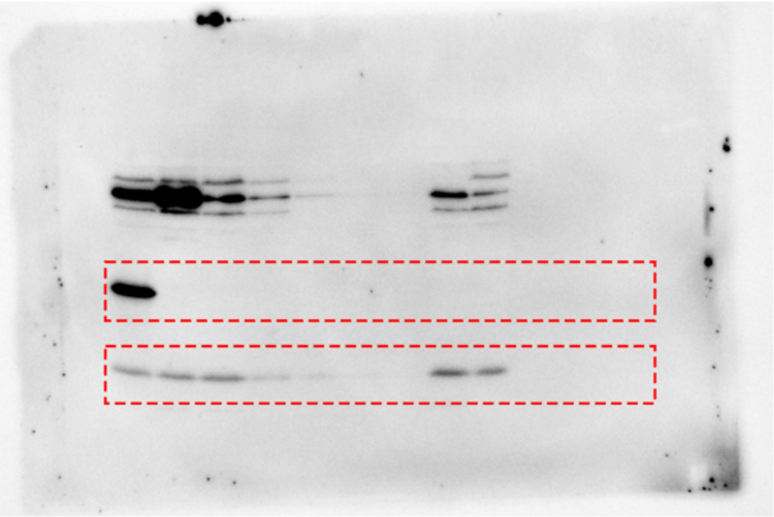

BCL2

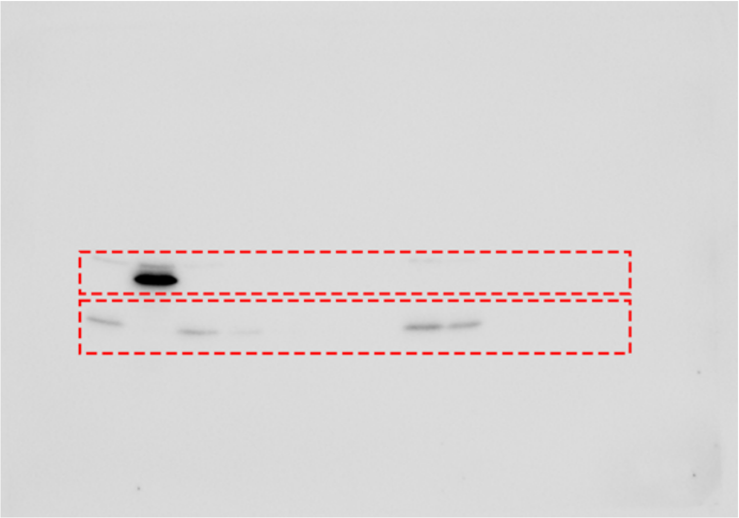

BCL-XL

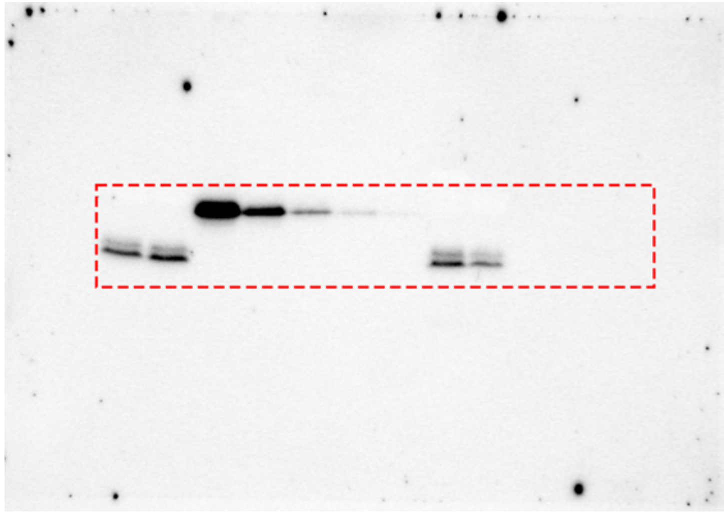

MCL1

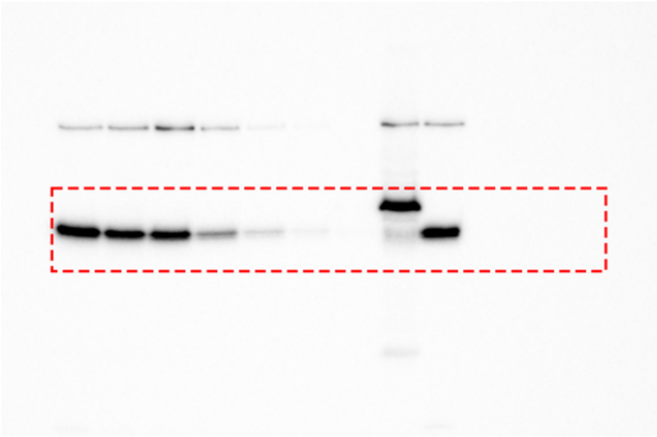

mAID

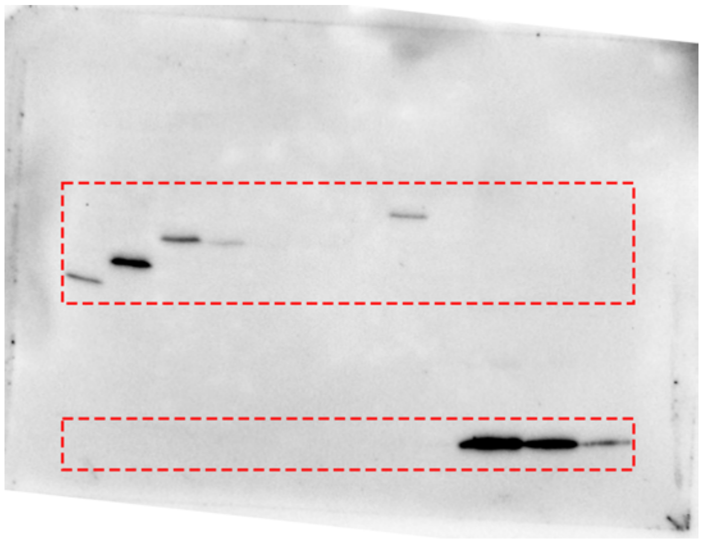

Actin

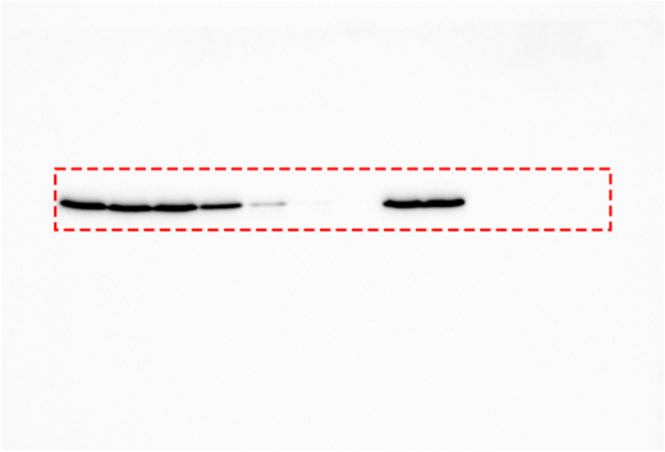

Original Western blots of Figure S3

Fig S5A

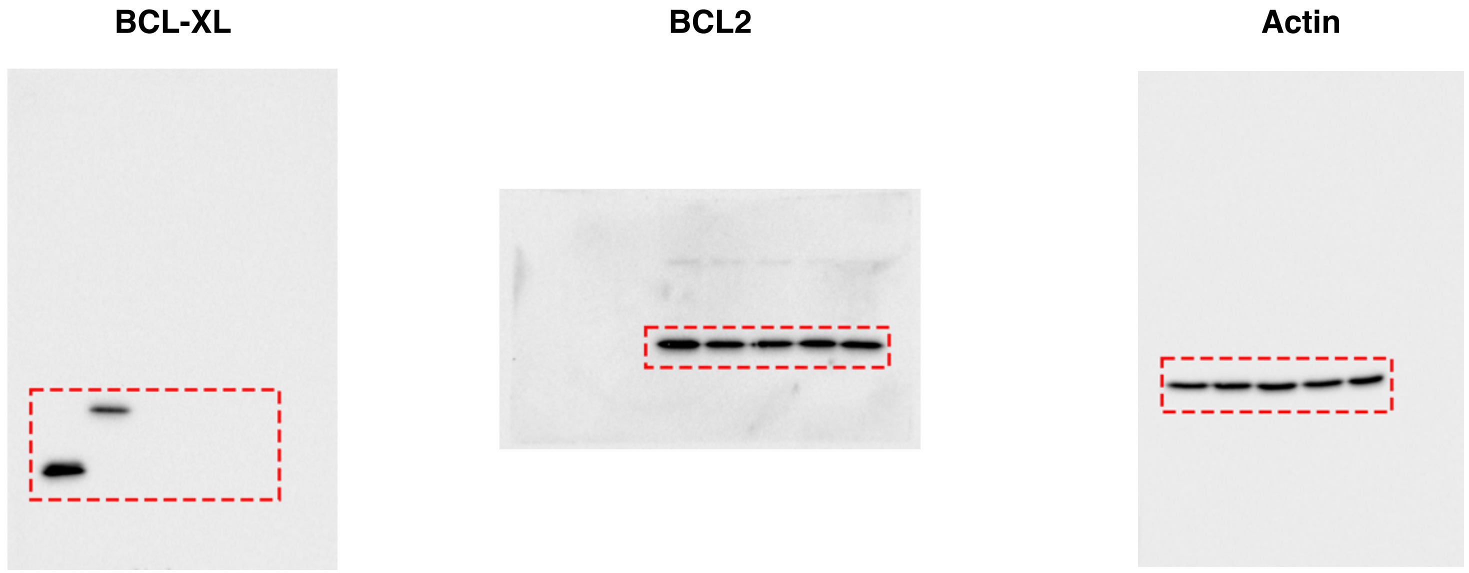

Fig S5B

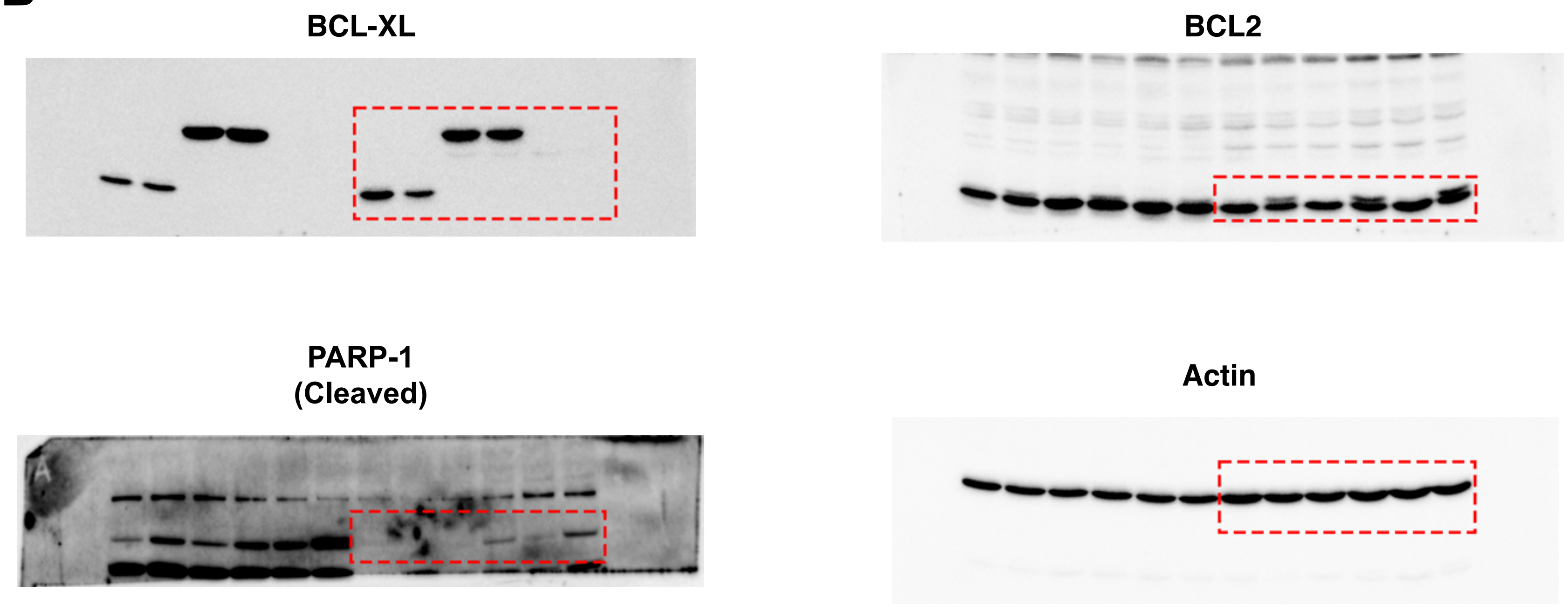

Original Western blots of Figure S5
