# Supplementary material for: BCL-XL regulates the timing of mitotic apoptosis independently of BCL2 and MCL1 compensation
Source: Cell Death Dis. 2024 Jan 3;15(1):2. doi: 10.1038/s41419-023-06404-9 (PMC10764939; doi:10.1038/s41419-023-06404-9)
Supplement: Supplementary file 2 — Supplemental Figures [file 41419_2023_6404_MOESM2_ESM.pdf]

SUPPLEMENTAL FIGURE LEGENDS

Figure S1. Inverse relation between BCL2 and BCL-XL in cell lines.

(A) mRNA expression of BCL2 versus BCL-XL. The levels of mRNA expression in 1,406 cell lines were retrieved from DepMap (depmap.org). The dataset 22Q2 Public was used. Pearson correlation: -0.287; p-value: 4.36×10^{-28}.

(B) Protein expression of BCL2 and BCL-XL in different cell lines. Expression of BCL2 and BCL-XL was detected with immunoblotting lysates from various asynchronously growing normal and cancer cell lines (upper panel). Band intensity was quantified and normalized to actin. Expression of BCL2 and BCL-XL in HeLa was arbitrarily set as 1. Cell origins: HeLa (cervical carcinoma), OVCA420 (ovarian carcinoma), MCF7 (mammary gland adenocarcinoma), MDA-MD-231 (breast adenocarcinoma), HCT116 (colorectal carcinoma), HT29 (colorectal adenocarcinoma), MIHA (immortalized hepatocytes), Hep3B (hepatocellular carcinoma), A549 (lung carcinoma), H1299 (lung carcinoma), U2OS (osteosarcoma), 293 (transformed human embryonic kidney), and RPE1 (hTERT-immortalized retinal pigment epithelial).

Figure S2. Different expression of mAID-tagged proteins can be achieved by varying the number of AU elements.

(A) Generation of ^{mAID}BCL-XL^{KO} cells with different number of AU elements. HeLa cells expressing ^{mAID}BCL-XL, tTA, and TIR1 were established using ^{mAID}BCL-XL constructs containing the indicated number of AU elements. Endogenous BCL-XL was concurrently disrupted with CRISPR-Cas9. After selection, a mixed population of cells was cultured in the presence or absence of DI. After 24 h, the cells were harvested and analyzed with immunoblotting.

(B) Generation of ^{mAID}BCL2^{KO} cells with different number of AU elements. HeLa cells stably expressing ^{mAID}BCL2, tTA, and TIR1 were established using ^{mAID}BCL2 containing the indicated number of AU elements. The endogenous BCL2 was at the same time disrupted with CRISPR-Cas9. After selection, a mixed population of the cells were cultured in either the presence or absence of DI. After 24 h, the cells were harvested and analyzed with immunoblotting.

Figure S3. BCL-XL is expressed at a lower level than BCL2 and MCL1.

(A) Relative expression of mAID-tagged BCL2, BCL-XL, BCL-W, and MCL1. Lysates from HeLa, ^{mAID}BCL-W (transienly transfect HeLa cells with ^{mAID}BCL-W in pUHD-SB-mAID/Hyg), ^{mAID}BCL2^{KO} (transienly transfect ^{mAID}BCL2^{KO} cells with mAID-BCL2 in pUHD-SB-mAID/Hyg), ^{mAID}BCL-XL^{KO} (3-fold serial dilution), and ^{mAID}MCL1^{KO} were analyzed using immunoblotting. Arrows and asterisks indicate the positions of the mAID-tagged and endogenous proteins, respectively.

(B) Recombinant mAID standards. mAID-H6 was induced in bacteria with IPTG. Lysates from untreated or IPTG-treated bacteria (two-fold dilution series) and BSA (two-fold dilution from 0.625 μg) were loaded onto SDS-PAGE and transferred to a membrane. Proteins on the membrane were visualized with Ponceau S staining, followed by blotting with antibodies against mAID.

(C) Relative expression of BCL-W, BCL2, BCL-XL, and MCL1 in HeLa cells. The concentrations of endogenous BCL-W, BCL2, BCL-XL, and MCL1 in HeLa lysates were estimated by comparing their expression to ^{mAID}BCL-W, ^{mAID}BCL2, ^{mAID}BCL-XL, and ^{mAID}MCL1 using a mAID-H6 standard curve (in ng of the corresponding protein per μg of HeLa cell extracts. Mean of two independent experiments.

Figure S4. BCL-XL is a critical regulator of mitotic apoptosis.

(A) Marginal acceleration of mitotic apoptosis in BCL2-deficient cells. Parental HeLa and ^{mAID}BCL2^{KO} cells were synchronized using a double thymidine procedure. Cells were trapped in mitosis using NOC and isolated by shake-off (t=0) before further incubated in NOC-containing medium. The cells were left untreated or treated with DI at the time of second thymidine release. Individual cells were tracked using live-cell imaging for 24 h (starting at 2 h after thymidine release) (*n*=50). Key: interphase (grey); mitosis (red); apoptosis (truncated bars).

(B) Acceleration of mitotic apoptosis in BCL-XL-deficient cells. HeLa and ^{mAID}BCL-XL^{KO} cells were synchronized and arrested in mitosis as described in panel A. The cells were left untreated or treated with DI at the time of second thymidine release. Individual cells were tracked using live-cell imaging for 24 h, starting at 2 h after thymidine release (*n*=50). Key: interphase (grey); mitosis (red); apoptosis (truncated bars).

Figure S5. BCL-XL inhibits mitotic apoptosis in RPE1 cells.

(A) Conditional depletion of BCL-XL. RPE1 cells expressing ^{mAID}BCL-XL, tTA, and TIR1 were generated. Endogenous BCL-XL was at the same time disrupted using CRISPR-Cas9. The cells were cultured in the presence or absence of DI and harvested at the indicated time points. Lysates were prepared and analyzed with immunoblotting. Lysates from parental RPE1 were loaded to show the expression of endogenous BCL-XL. Equal loading of lysates was confirmed by immunoblotting for actin.

(B) BCL-XL is an inhibitor of mitotic apoptosis in RPE1. ^{mAID}BCL-XL^{KO} RPE1 cells were incubated with DI and/or NOC as indicated. After 10 h, the cells were harvested and analyzed with immunoblotting.

(C) Acceleration of mitotic apoptosis in BCL-XL-deficient RPE1. ^{mAID}BCL-XL^{KO} RPE1 cells were incubated with NOC in the presence or absence of DI. Individual cells were tracked using live-cell imaging. Dot plots represent the elapsed time between mitotic entry and apoptosis (*n*=48; mean-/+SEM). The duration of mitotic arrest is plotted using Kaplan-Meier estimator. Mann-Whitney test: ***$p<0.001$. Raw data for individual cells are shown in the lower panel.

Figure S6. Predominance of BAX in mitotic apoptosis triggered by silencing BCL-XL.

Parental ^{mAID}BCL-XL^{KO} and ^{mAID}BCL-XL^{KO} cells lacking BCL2, BAX, or BAK were synchronized and arrested in mitosis as described in Fig 4A. The cells were either left untreated or incubated with DI at the time of the second thymidine release. Individual cells were tracked using live-cell imaging for 24 h, starting at 2 h after thymidine release (*n*=50). Key: interphase (grey); mitosis (red); apoptosis (truncated bars).

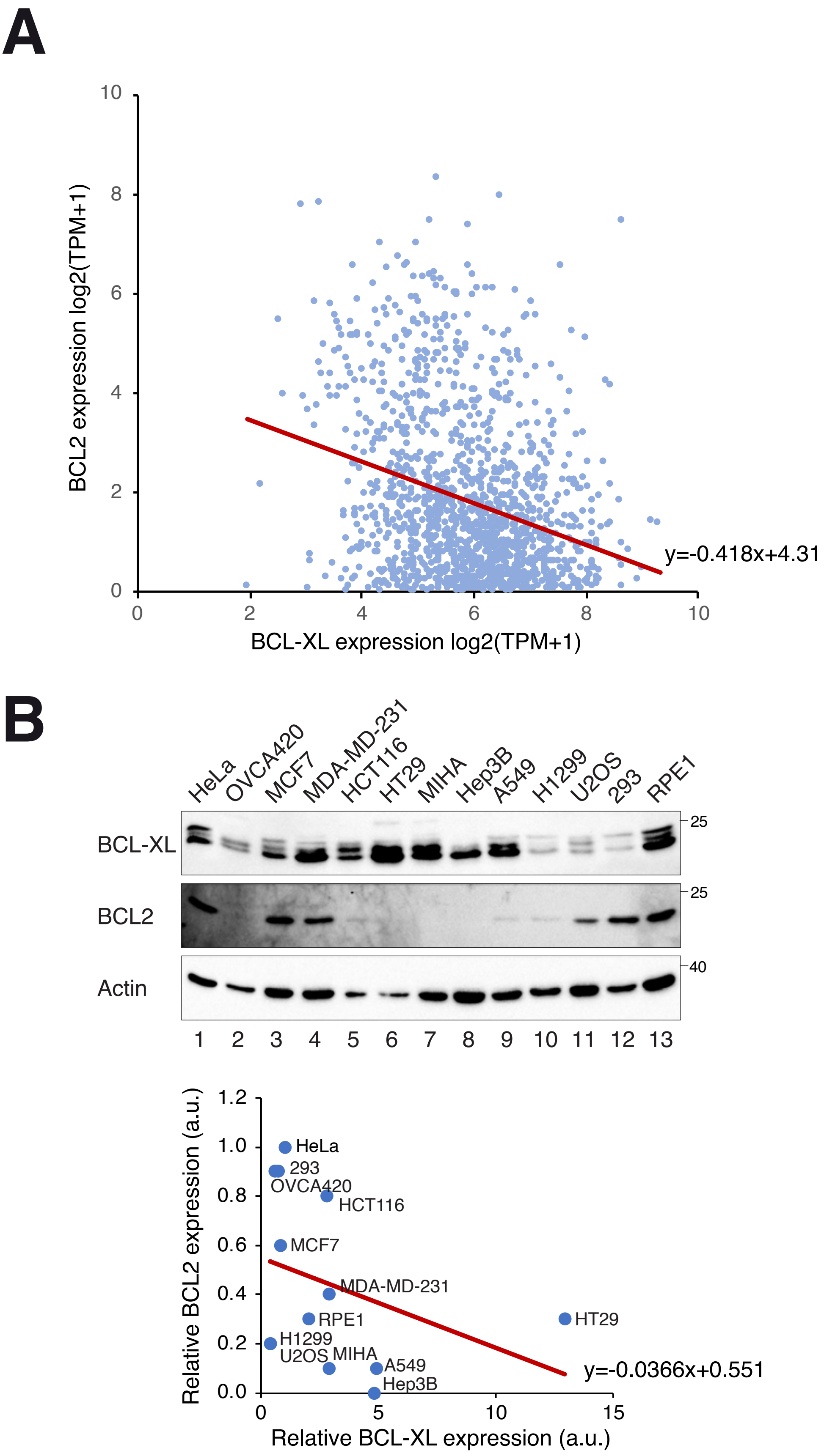

Supplemental Figure S1

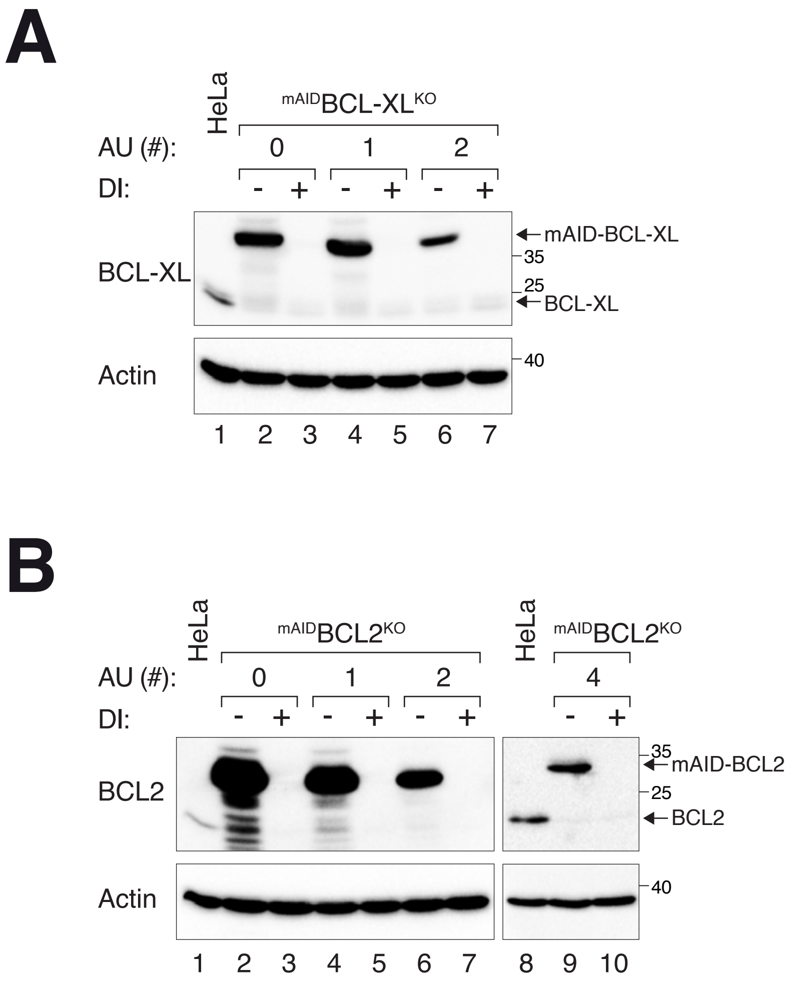

Supplemental Figure S2

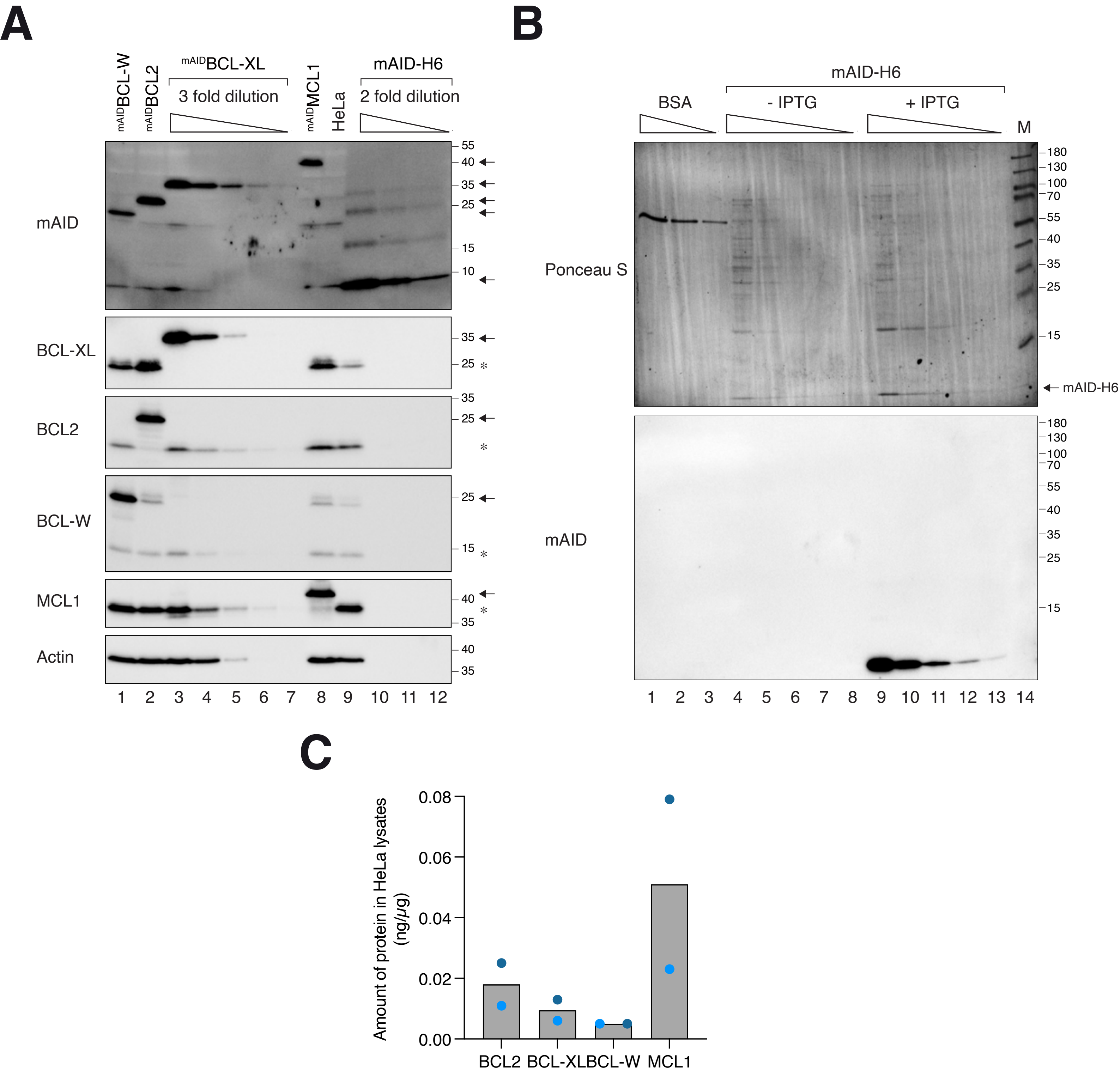

Supplemental Figure S3

A

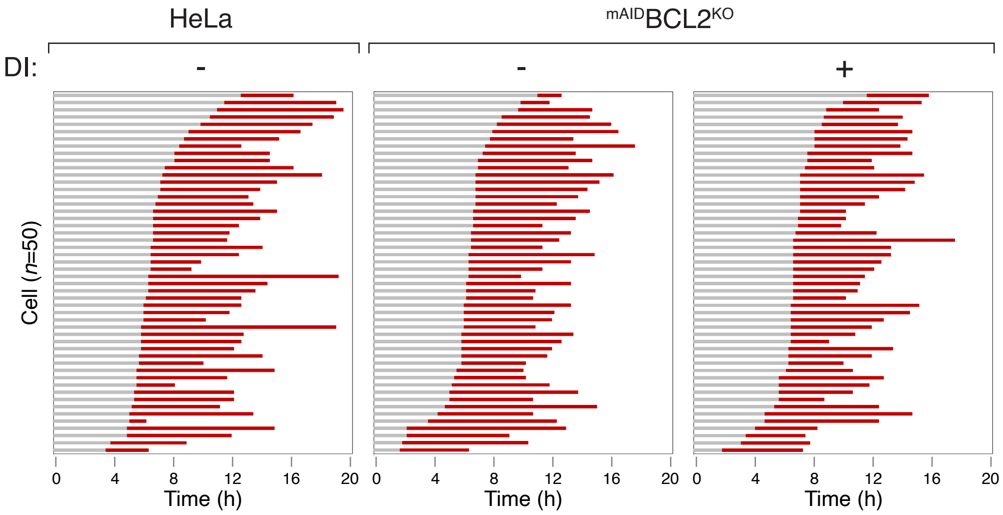
HeLa
mAIDBCL2KO
DI:
-
-
+
Cell (n=50)
0 4 8 12 16 20
Time (h)

B

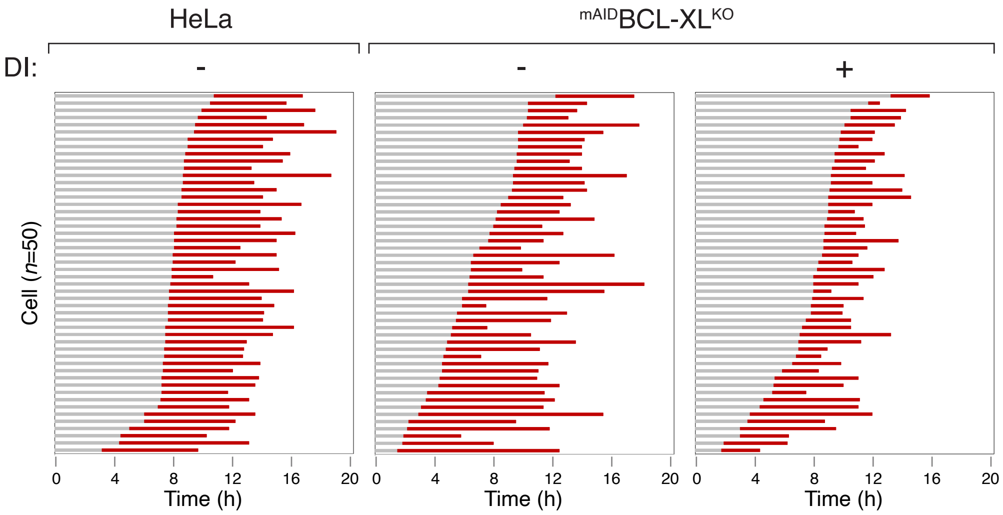
HeLa
mAIDBCL-XLKO
DI:
-
-
+
Cell (n=50)
0 4 8 12 16 20
Time (h)

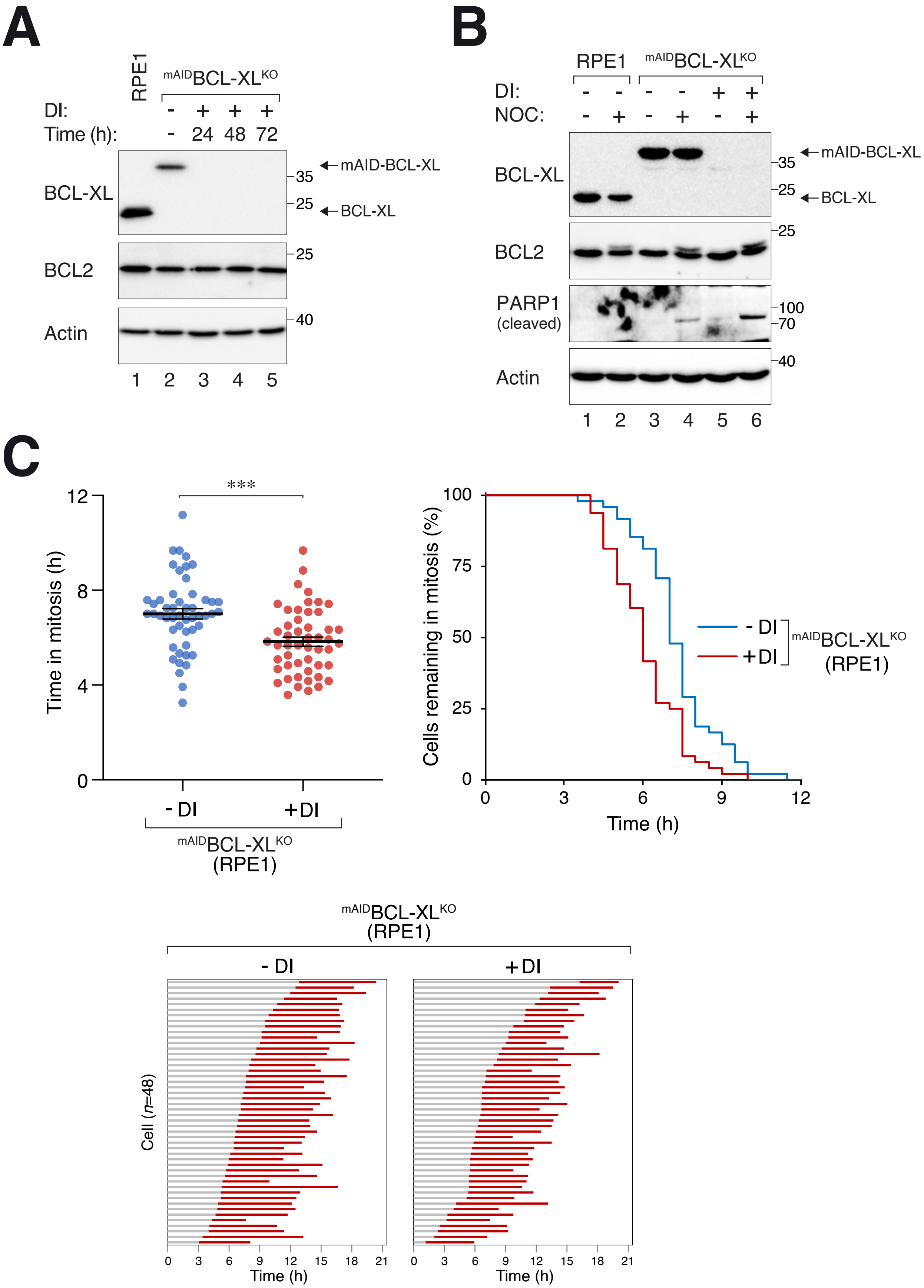

Supplemental Figure S5

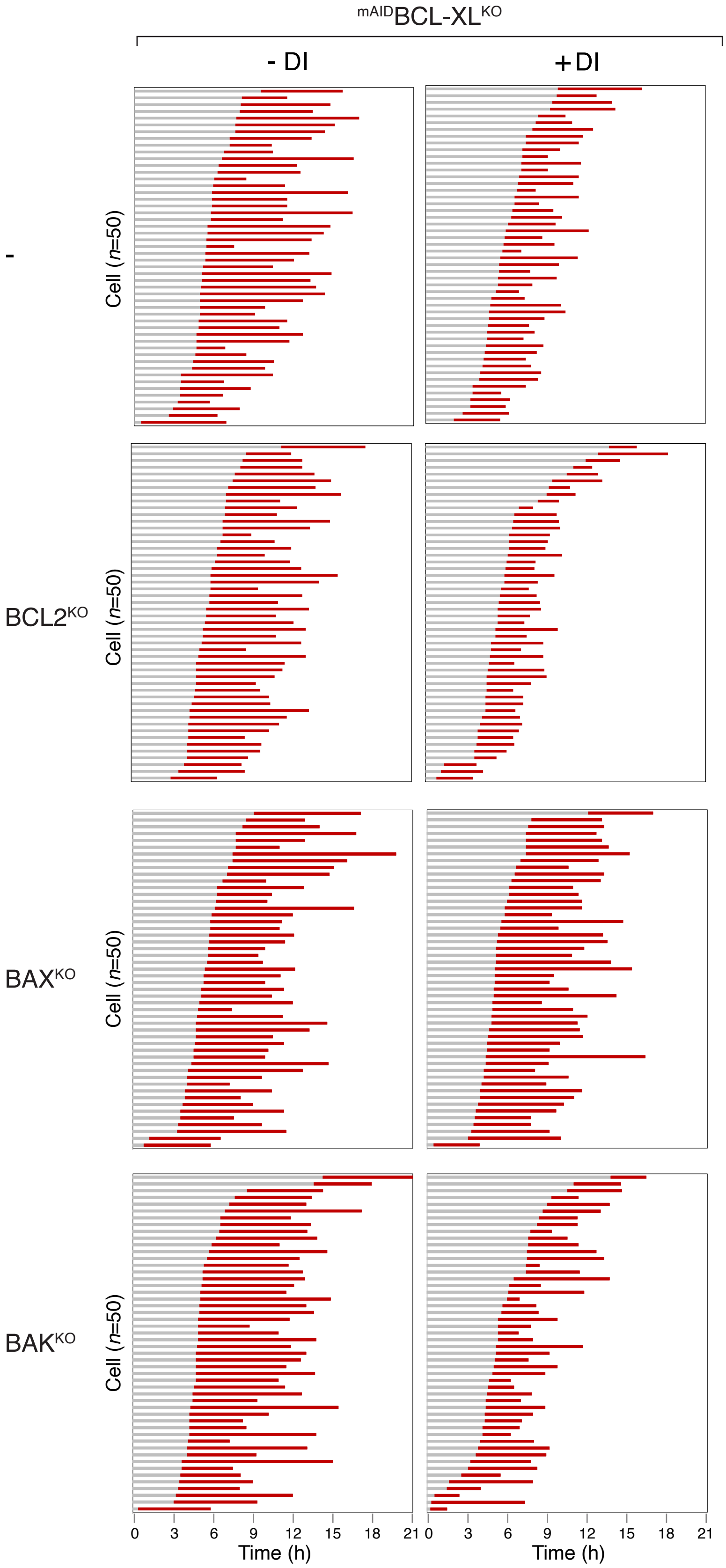

Supplemental Figure S6

Figure S7: Supplemental materials: uncropped Western blots

Boxes indicate the lanes and bands used in the Figures and Supplemental Figures.

Fig 1A

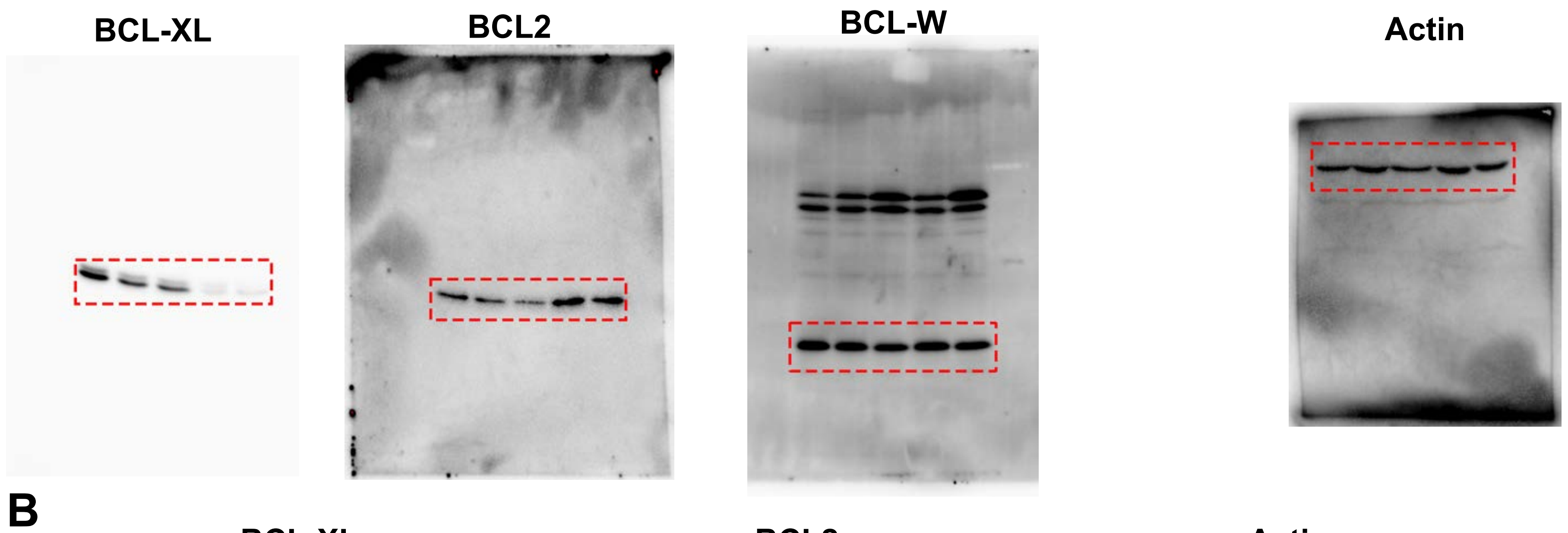

Fig 1B

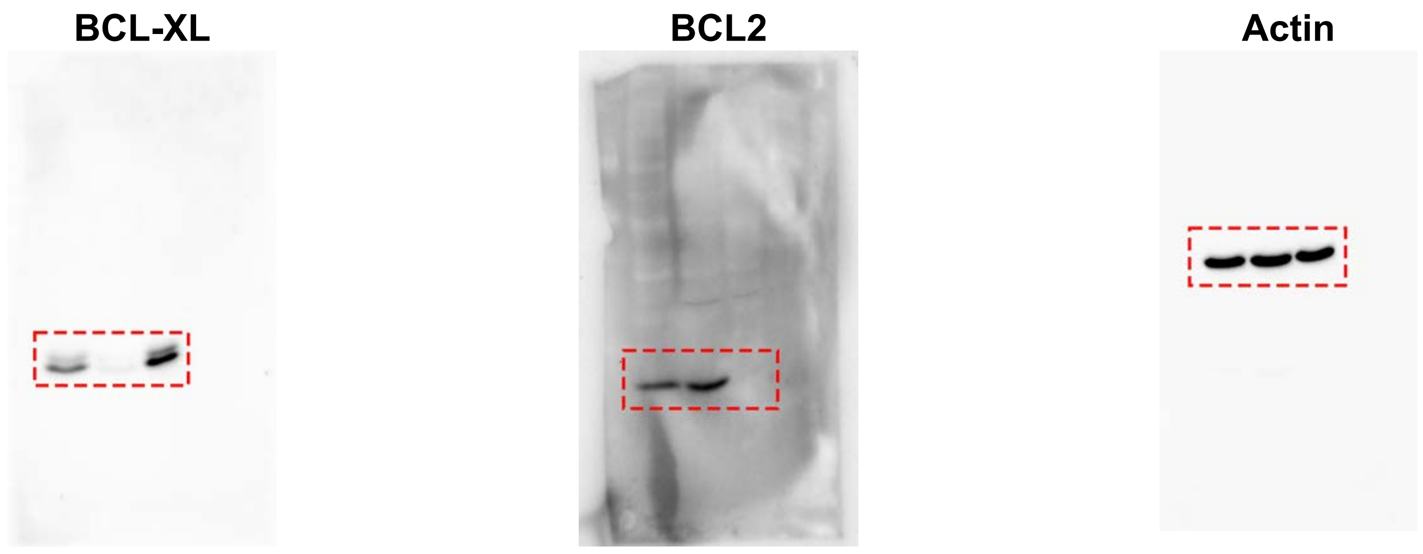

Fig 1C

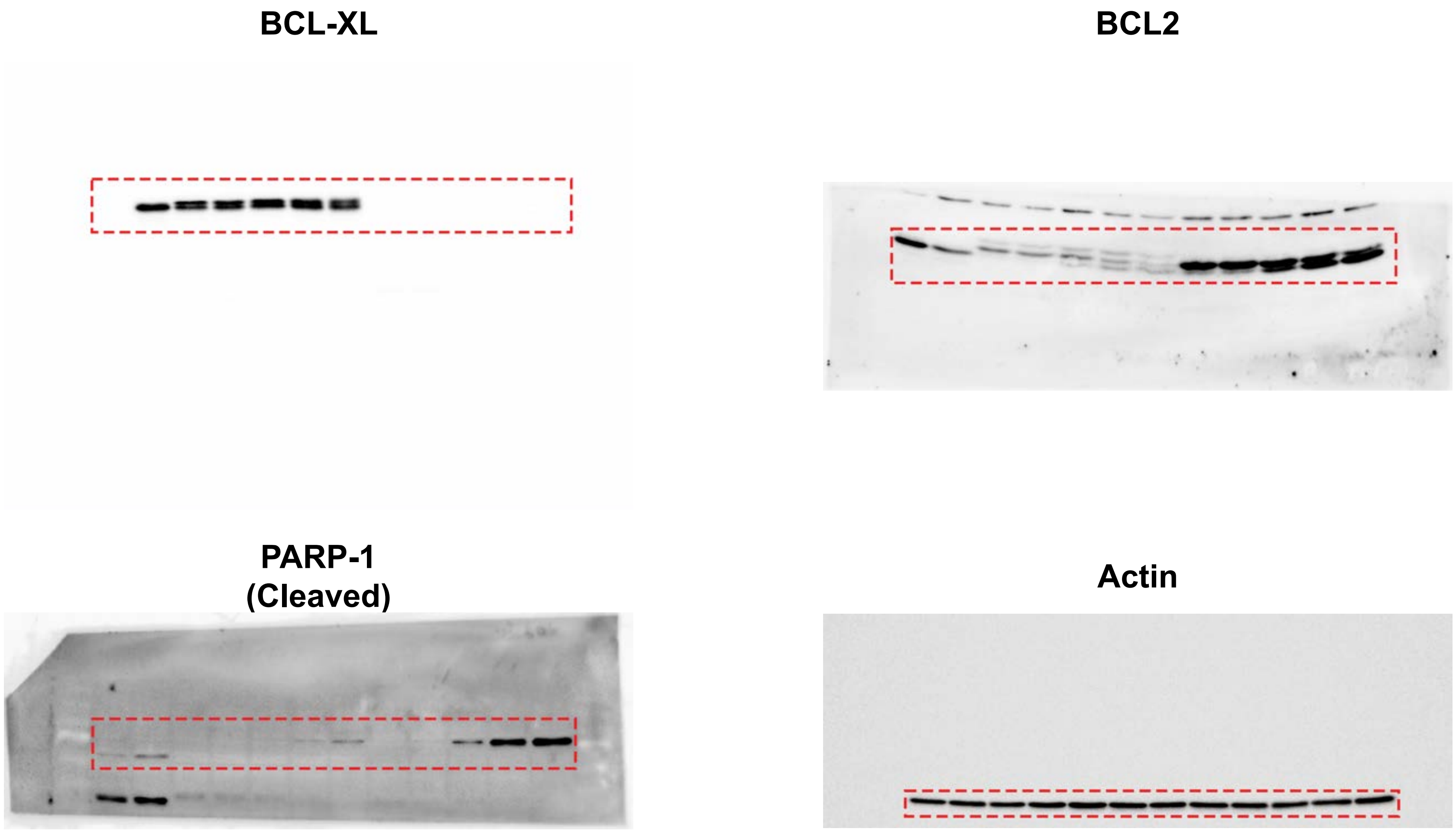

Fig 1D

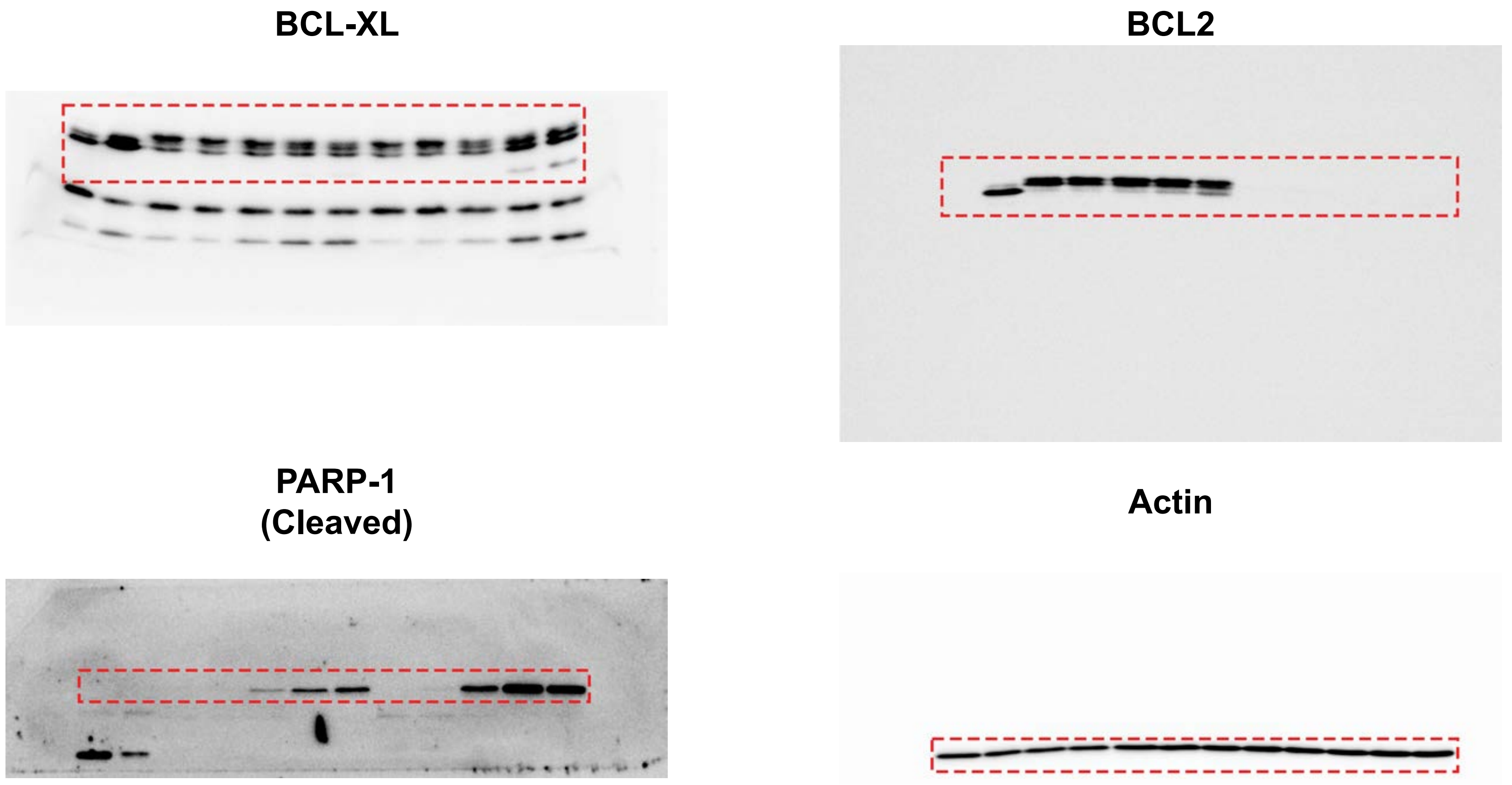

Original Western blots of Figure 1

Fig 2B

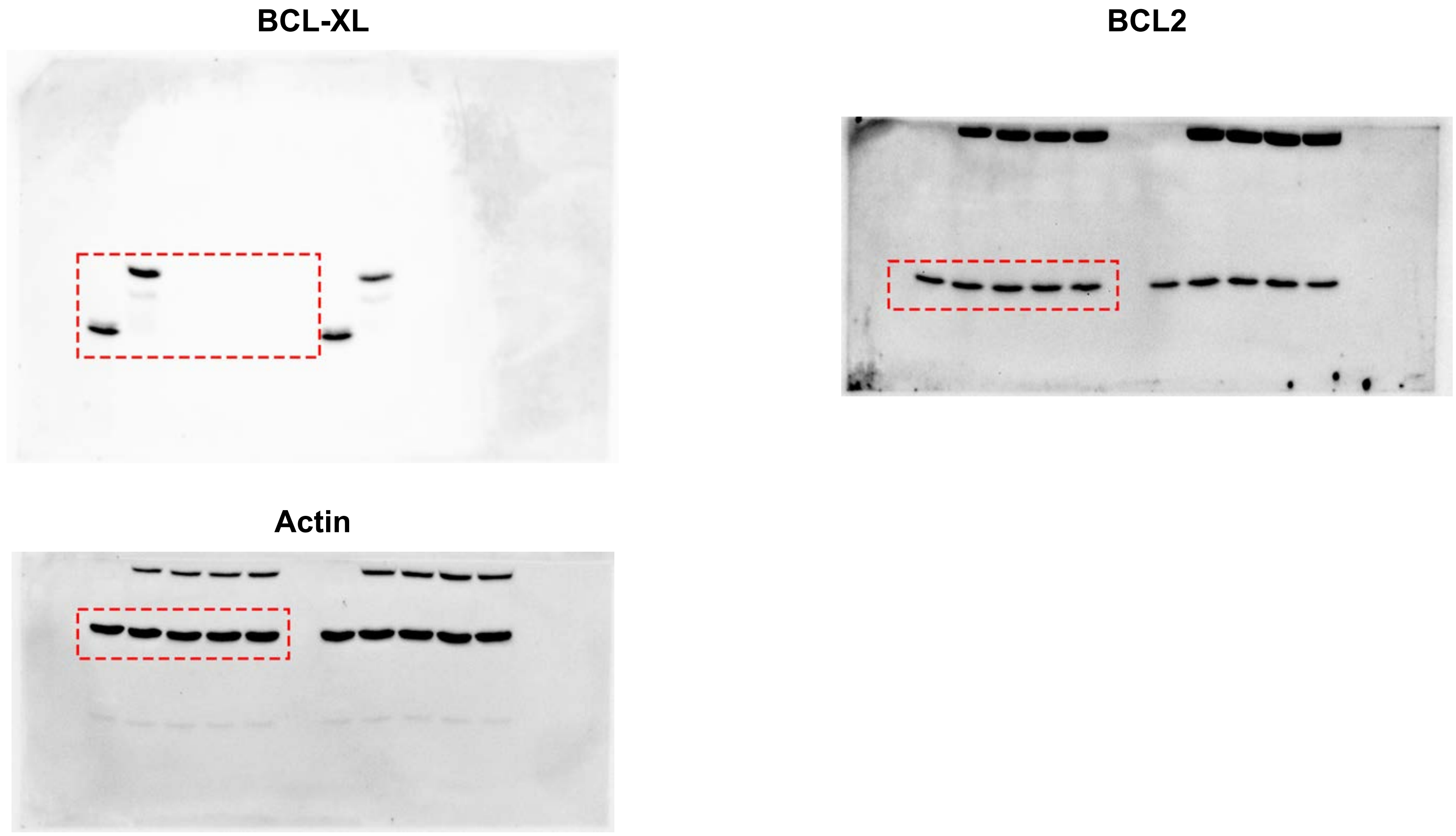

Fig 2C

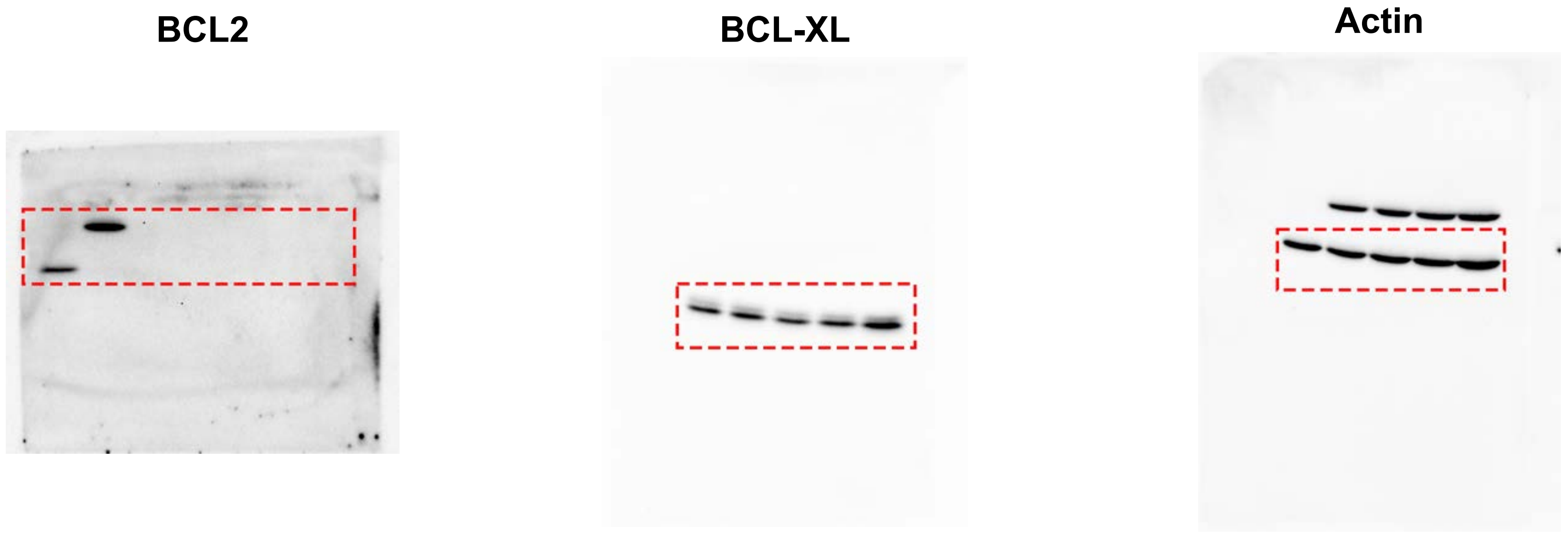

Original Western blots of Figure 2

Fig 3A

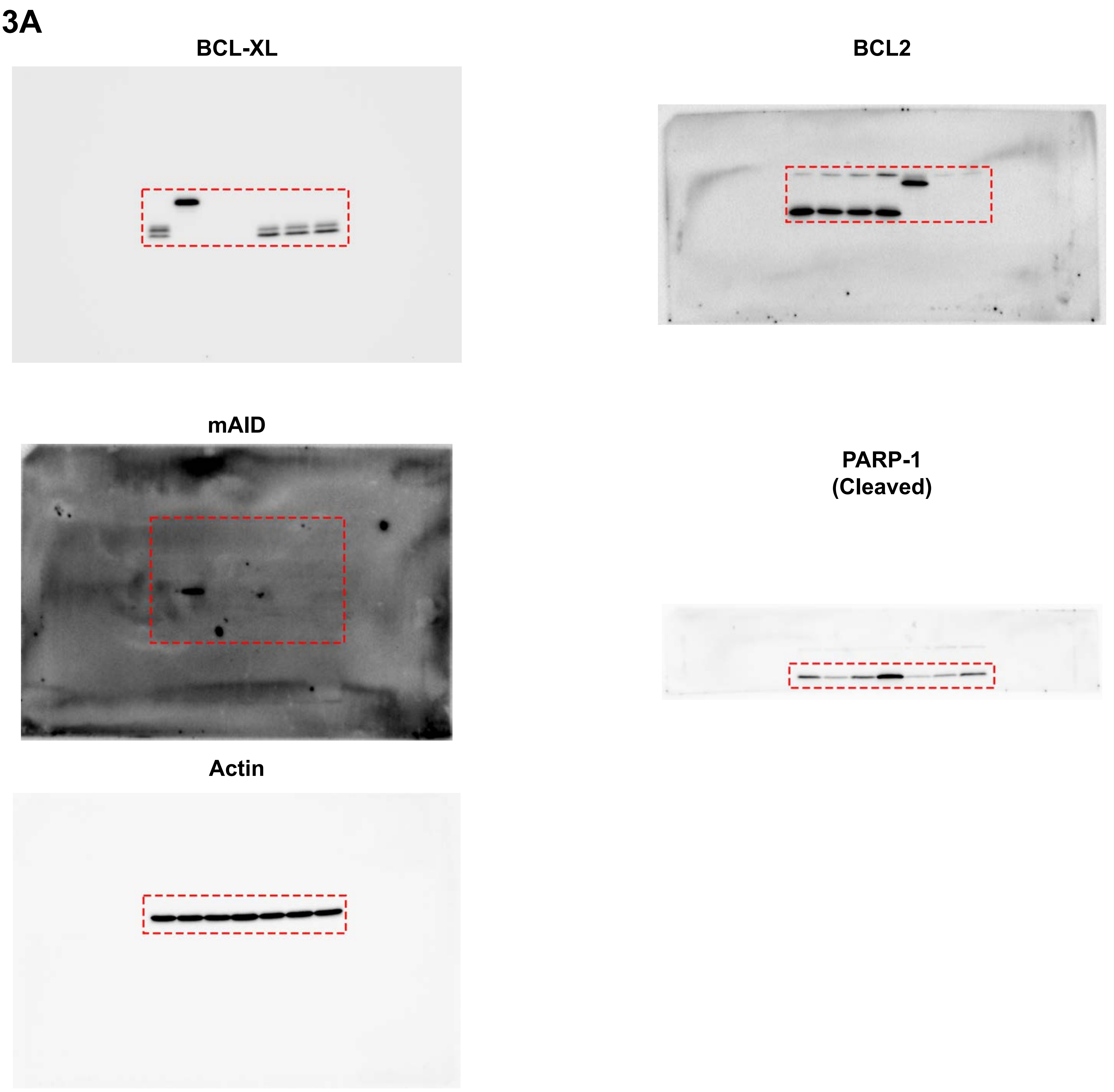

Fig 3B

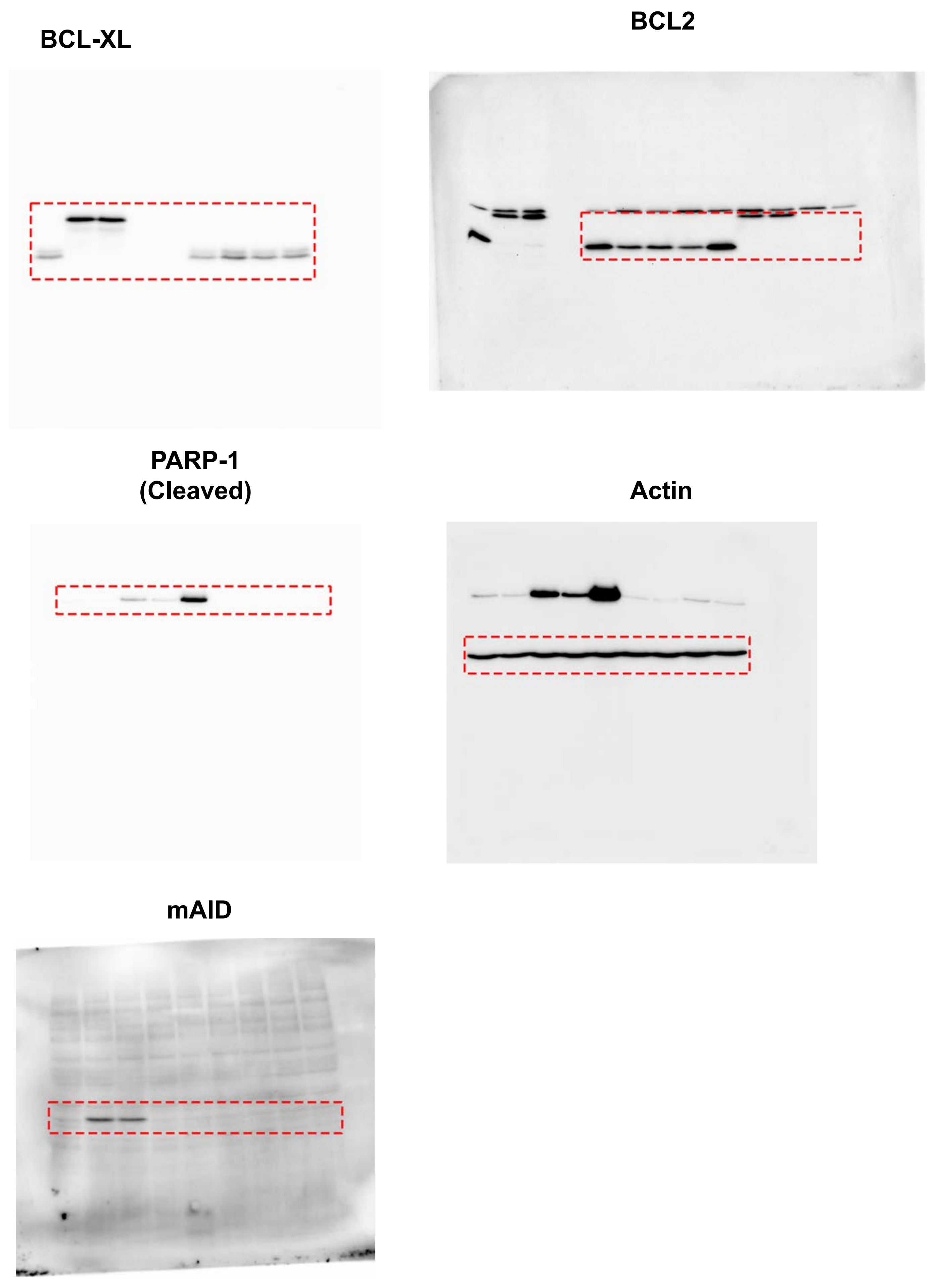

Fig 3C

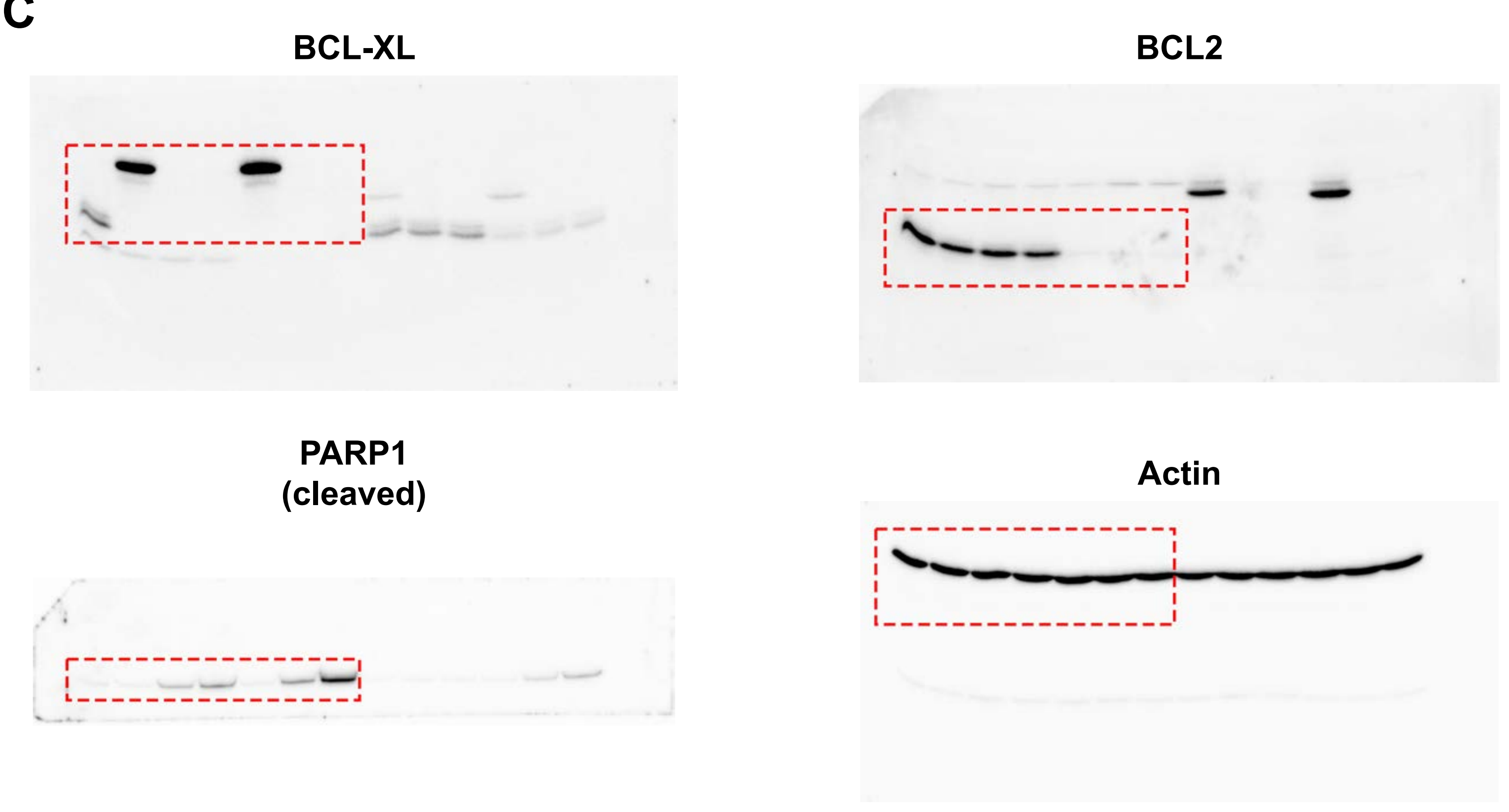

Fig 3D

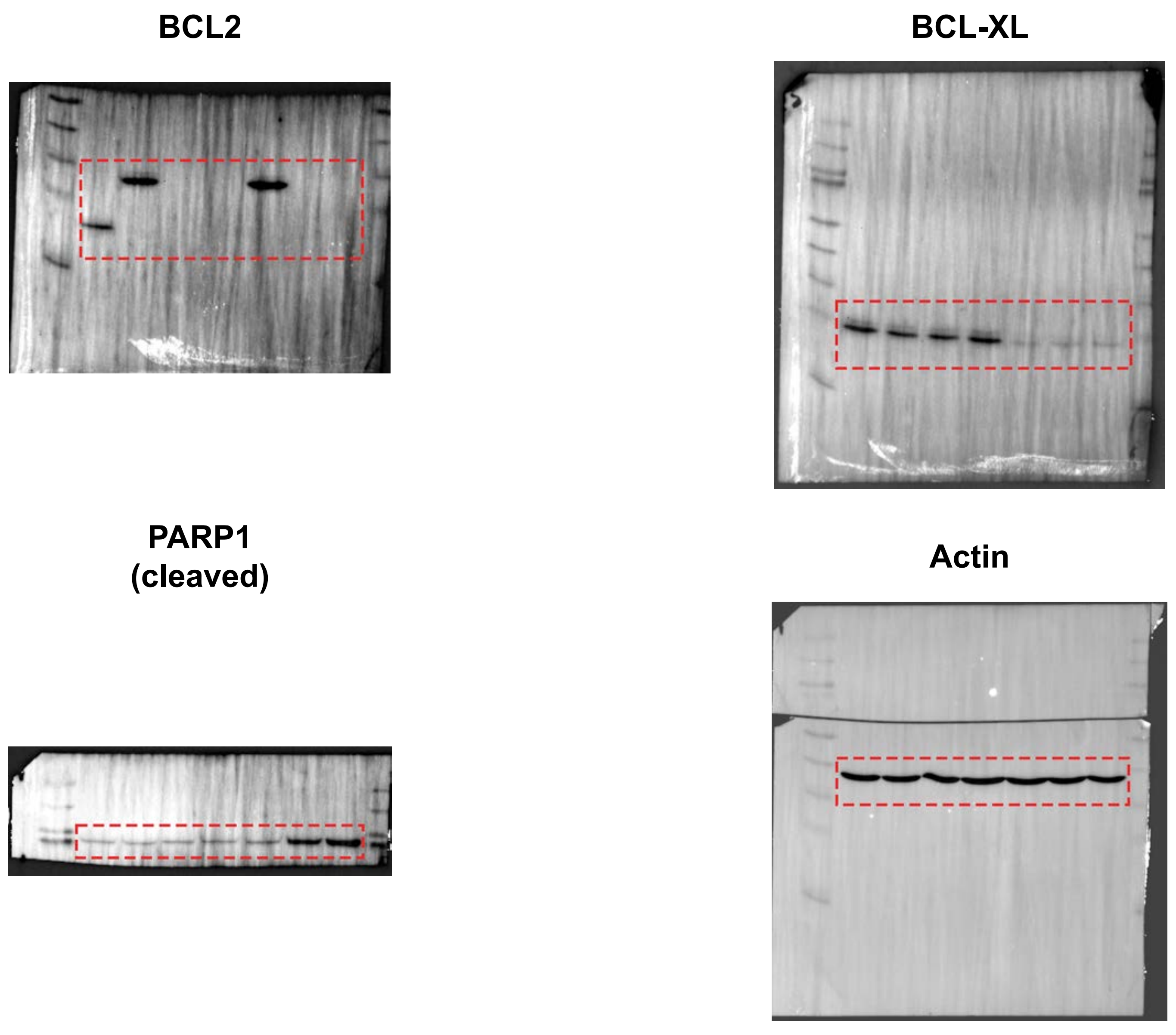

Fig 3E

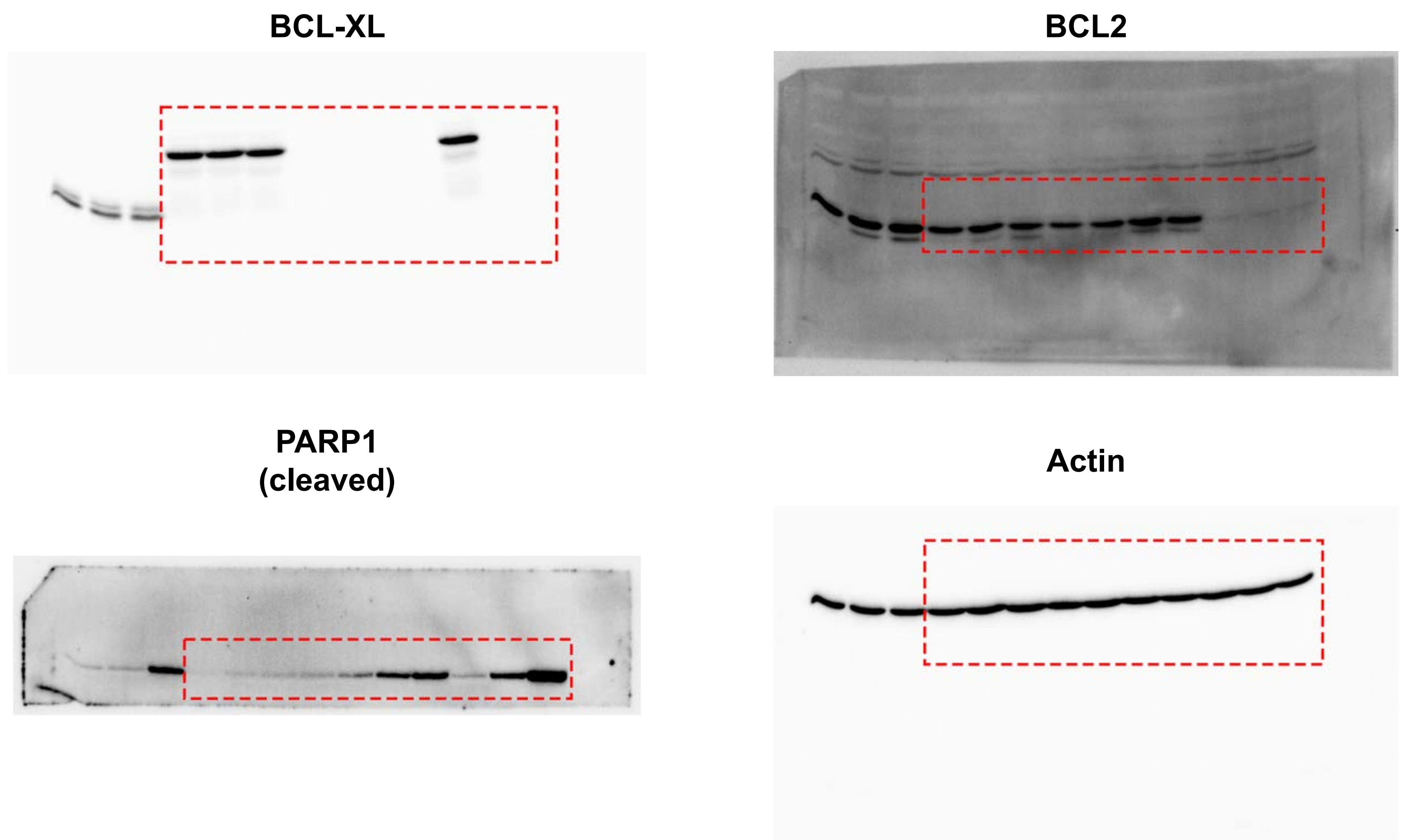

Original Western blots of Figure 3

Fig 4A

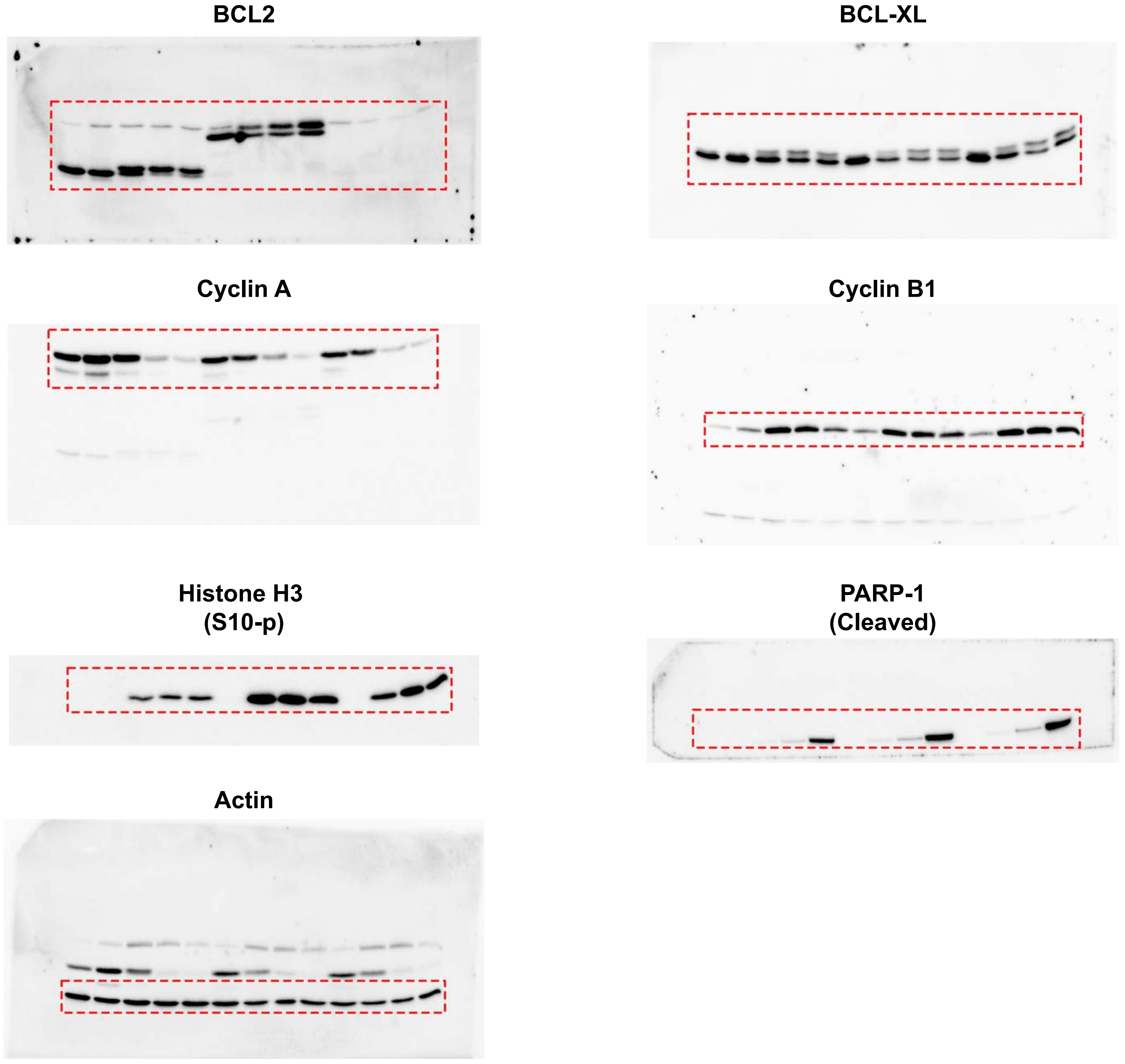

Fig 4B

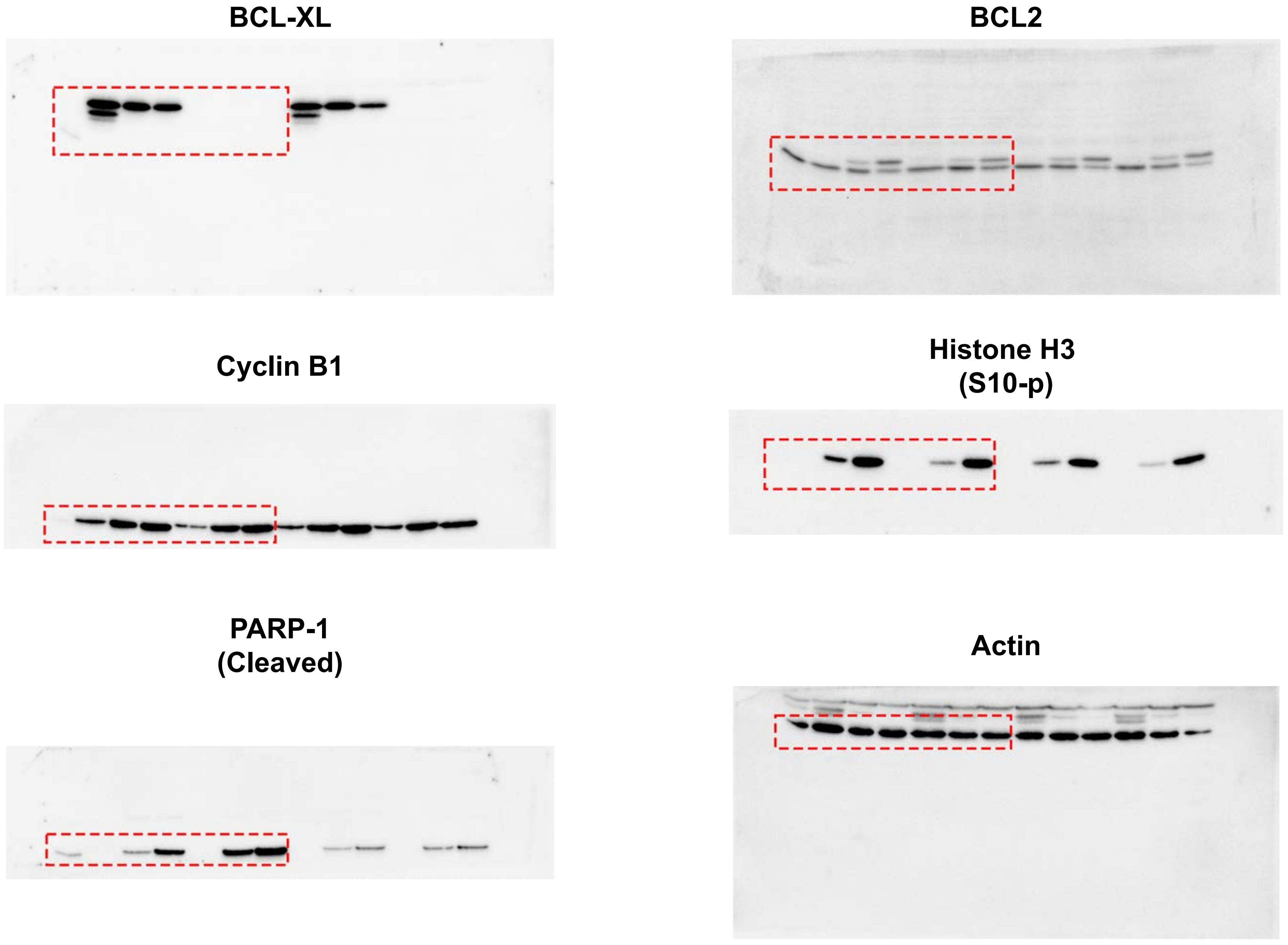

Original Western blots of Figure 4

Fig 5A

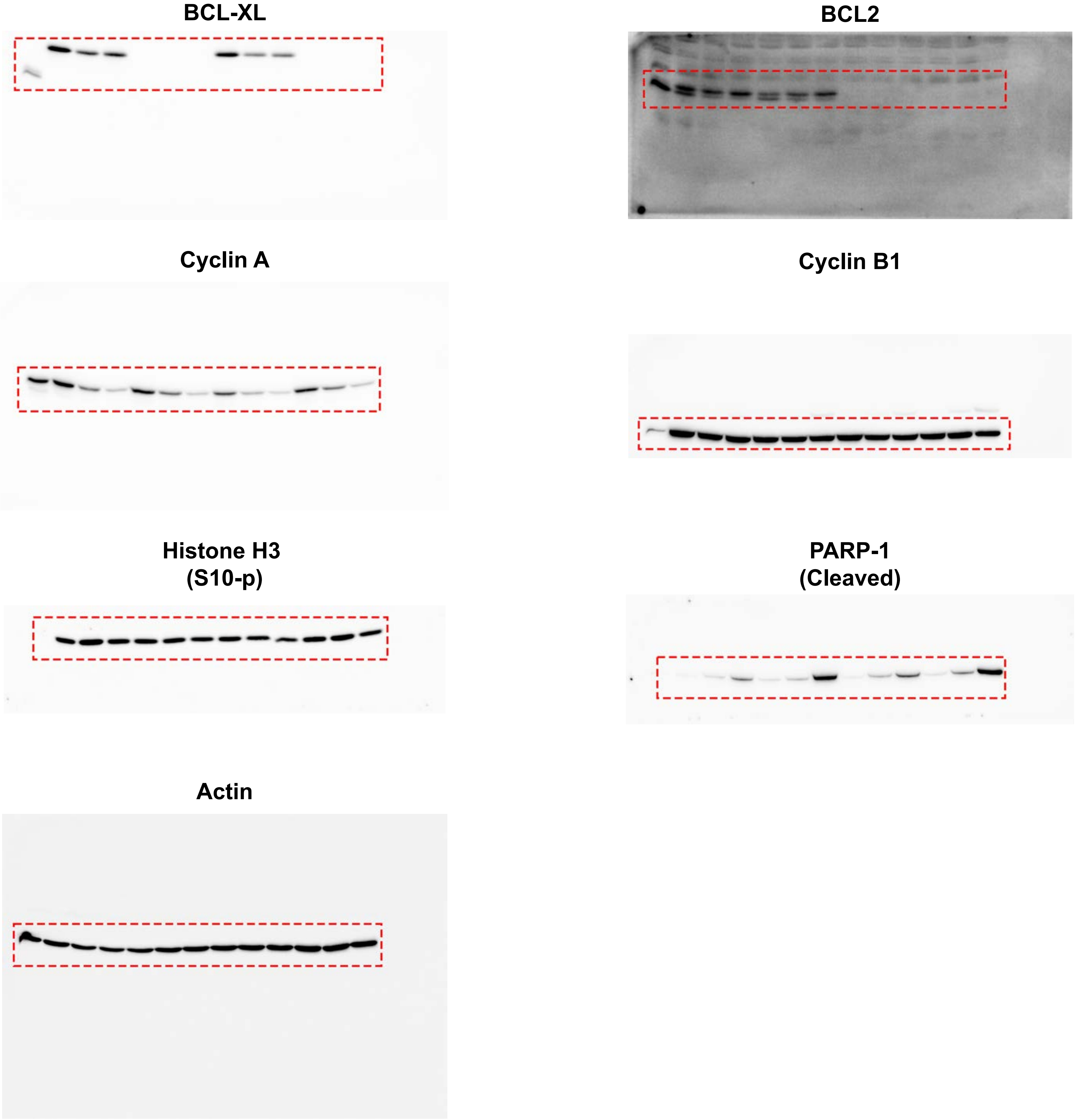

Original Western blots of Figure 5

Fig 6A

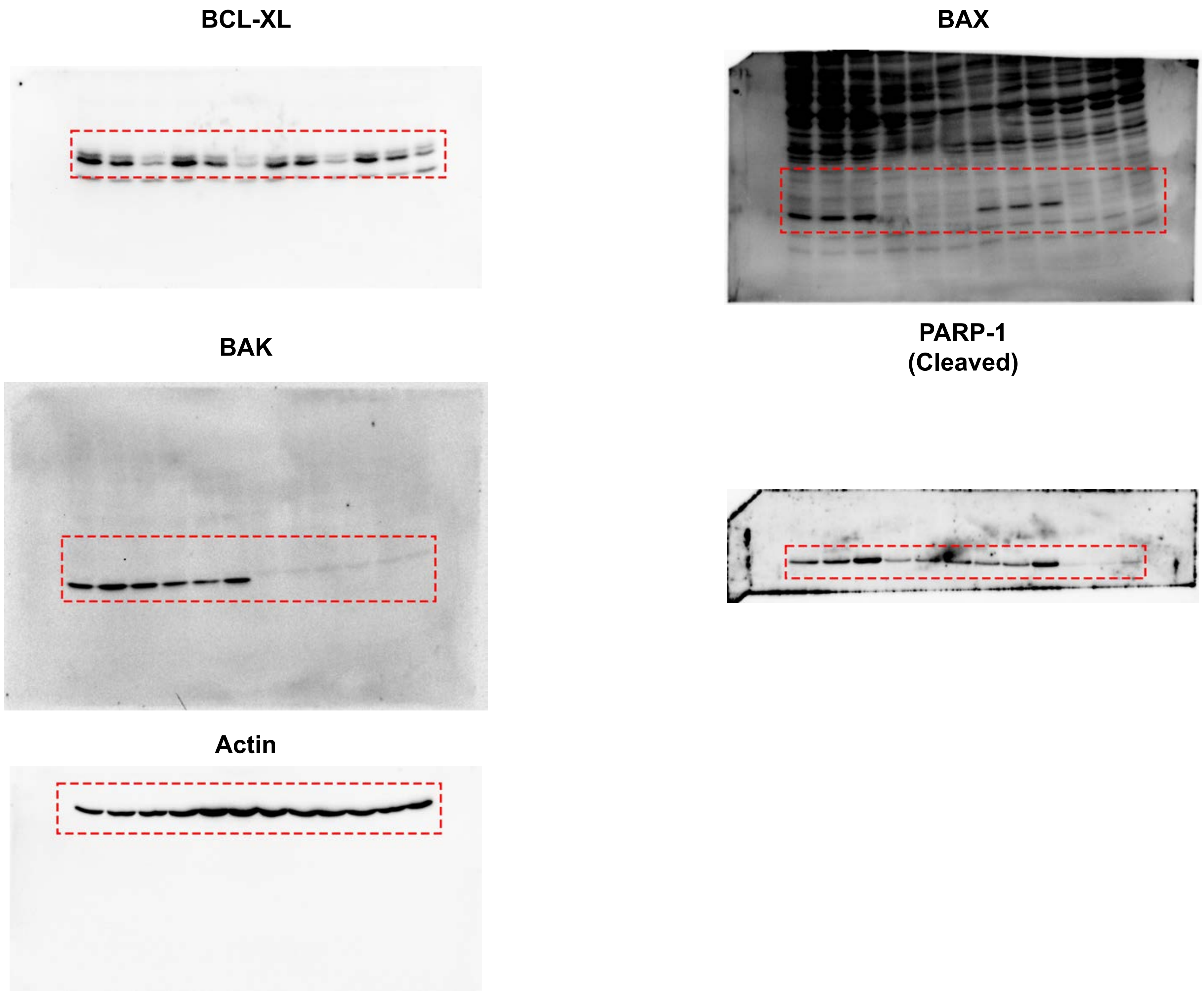

Fig 6B

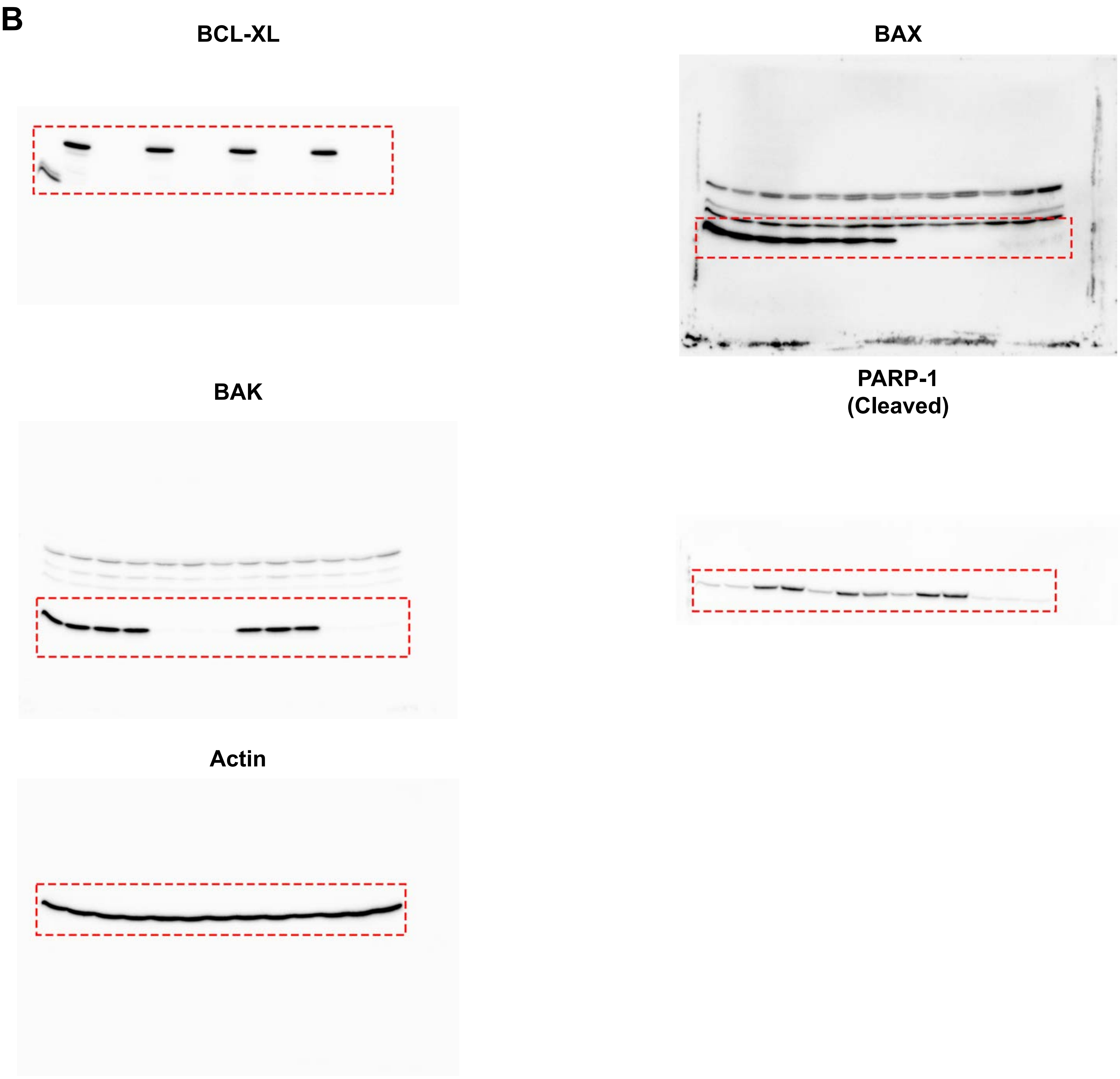

Fig 6C

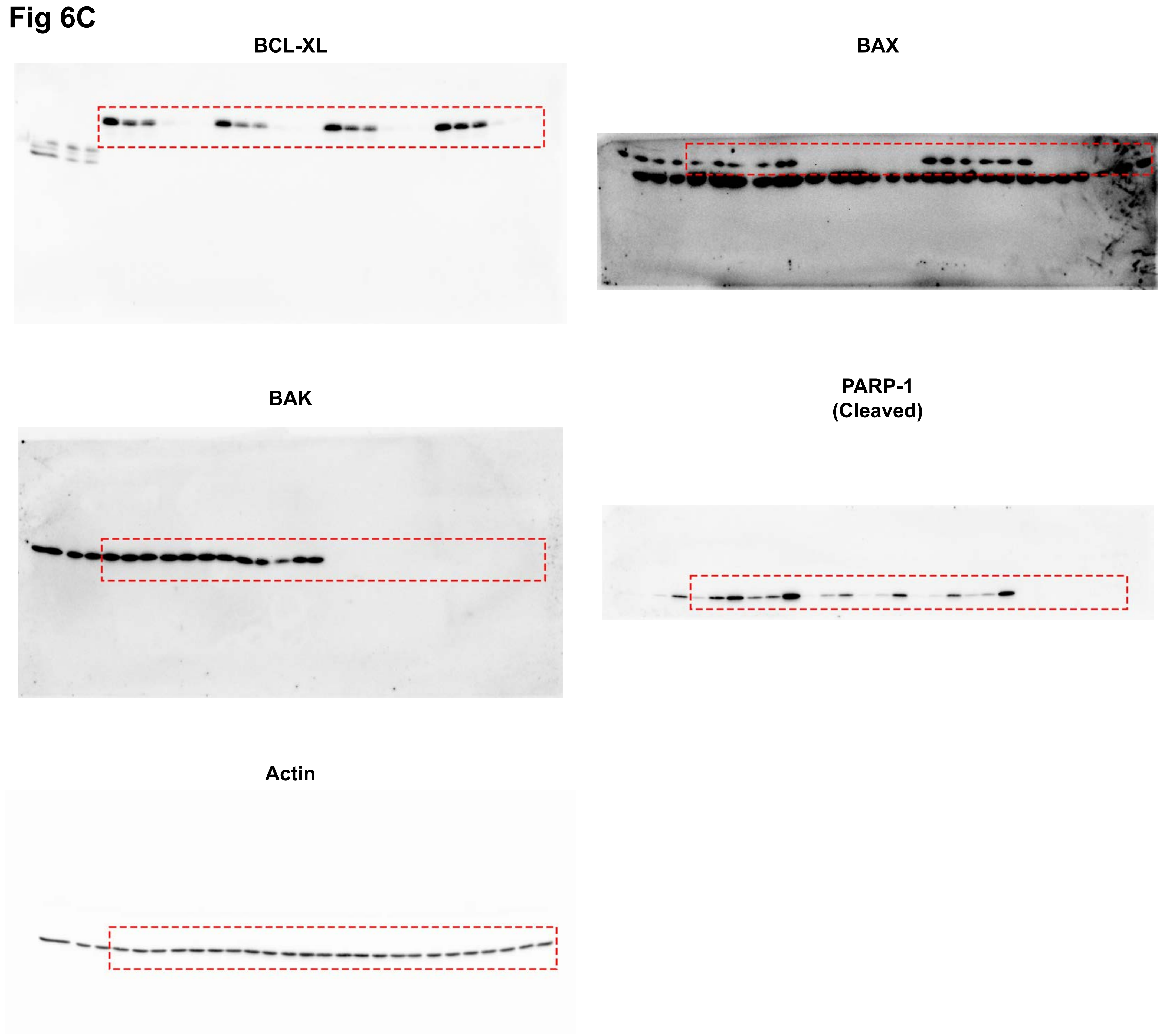

Original Western blots of Figure 6

Fig 7A

BCL-XL

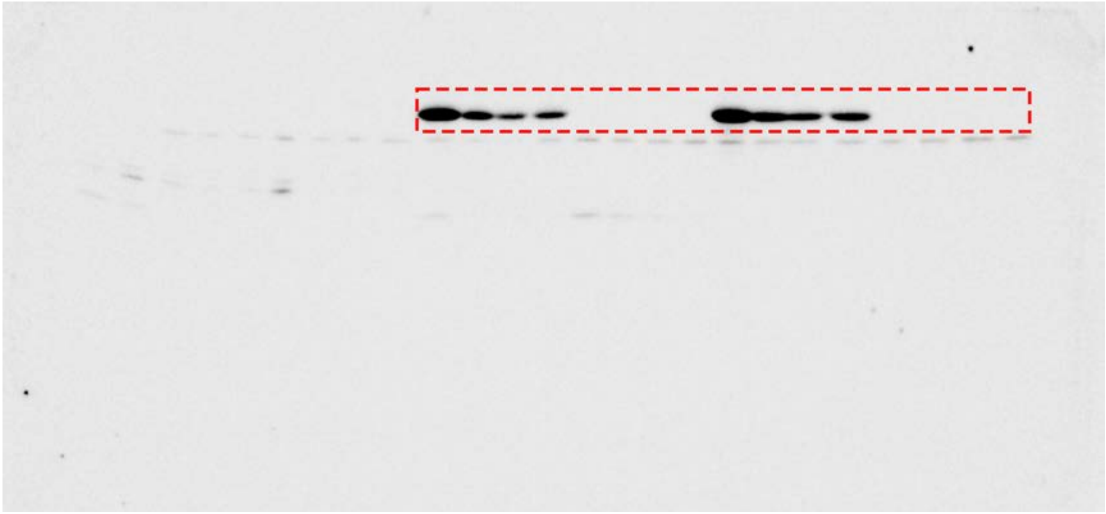

BCL2

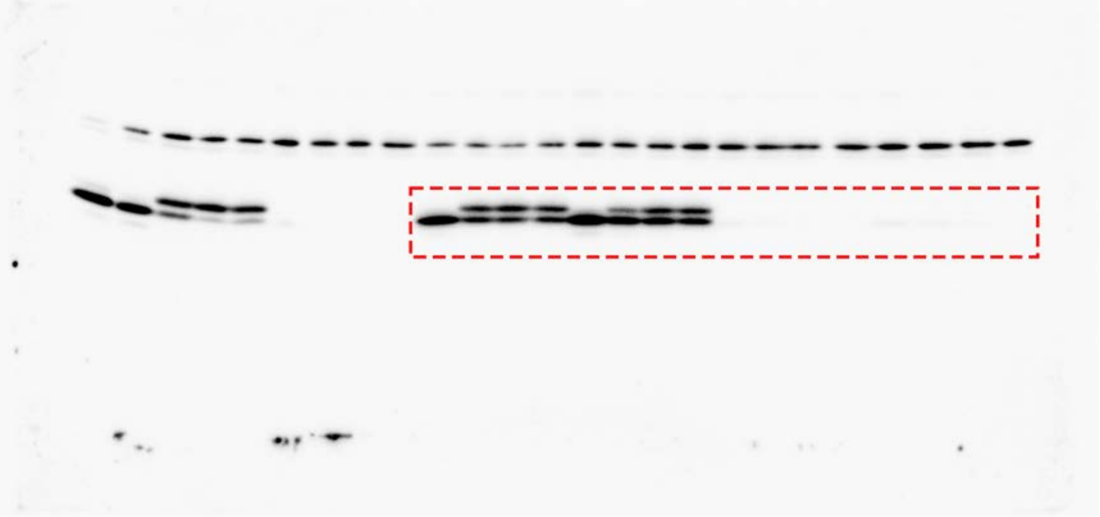

MCL1

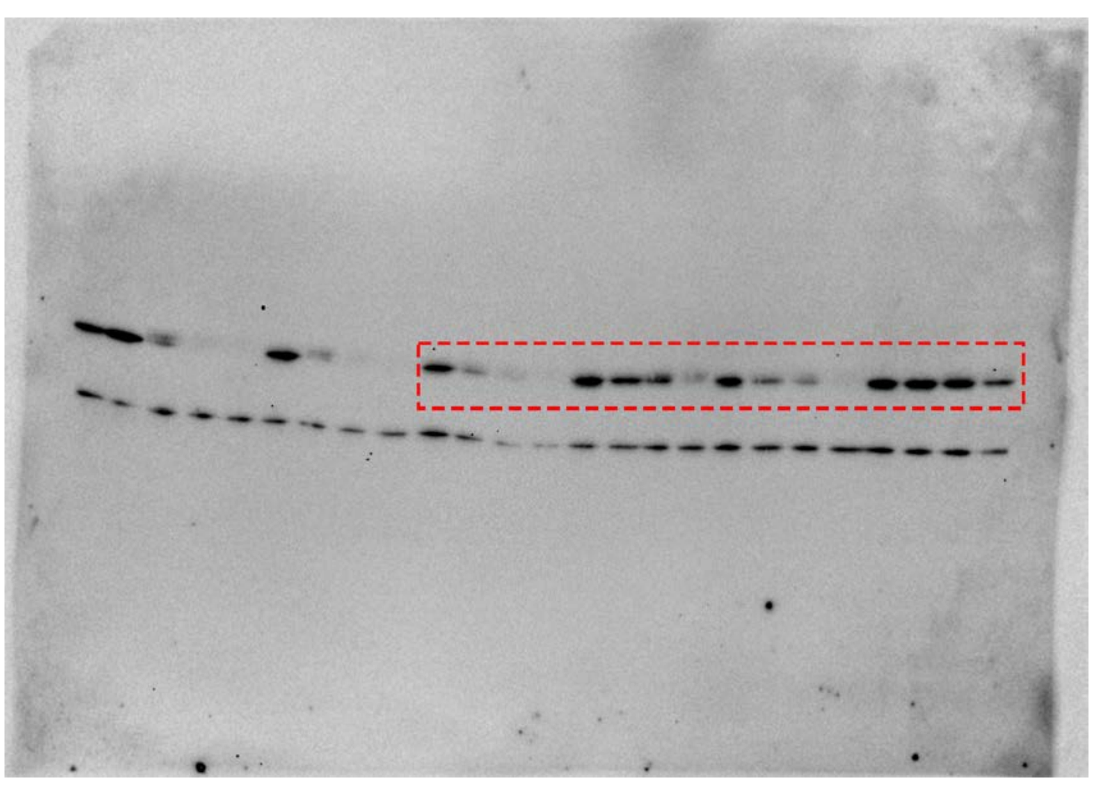

BCL-W

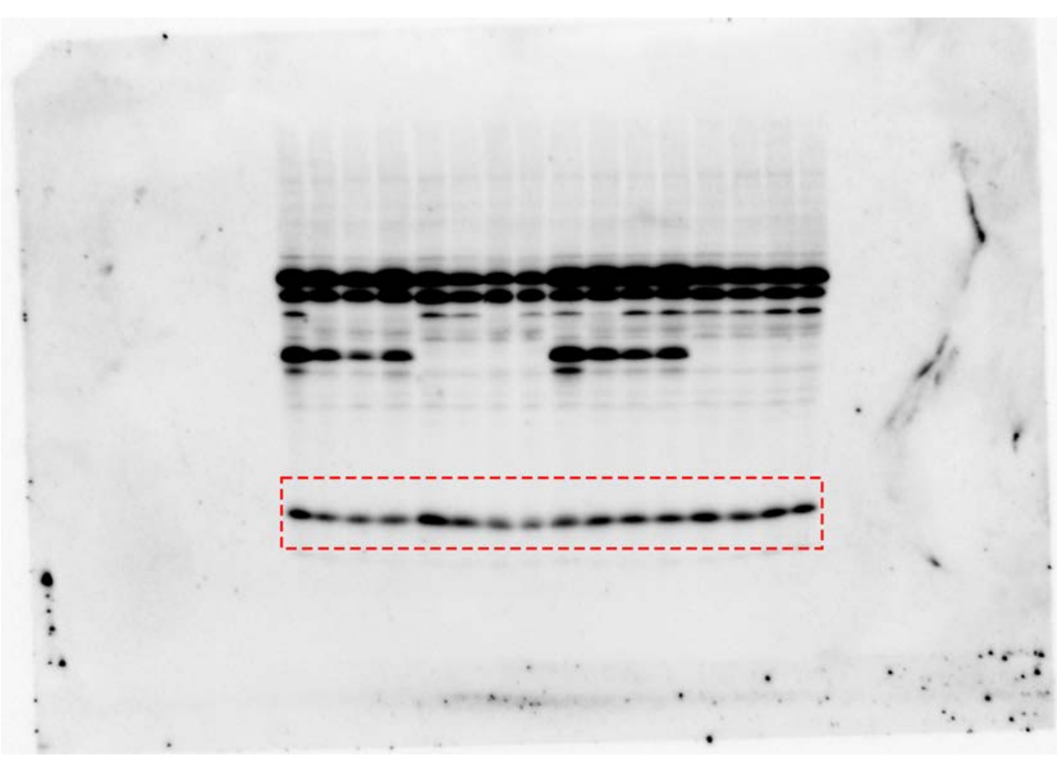

Actin

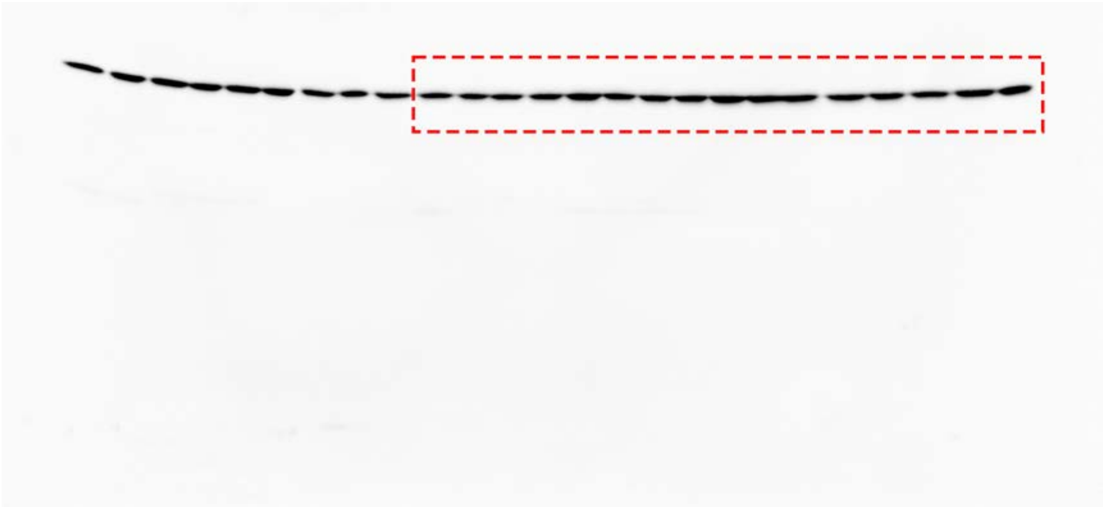

Fig 7B_Left

BCL-XL

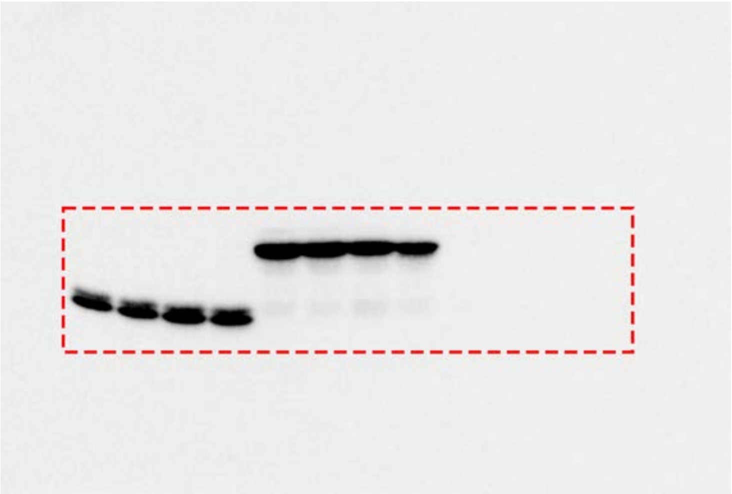

BCL2

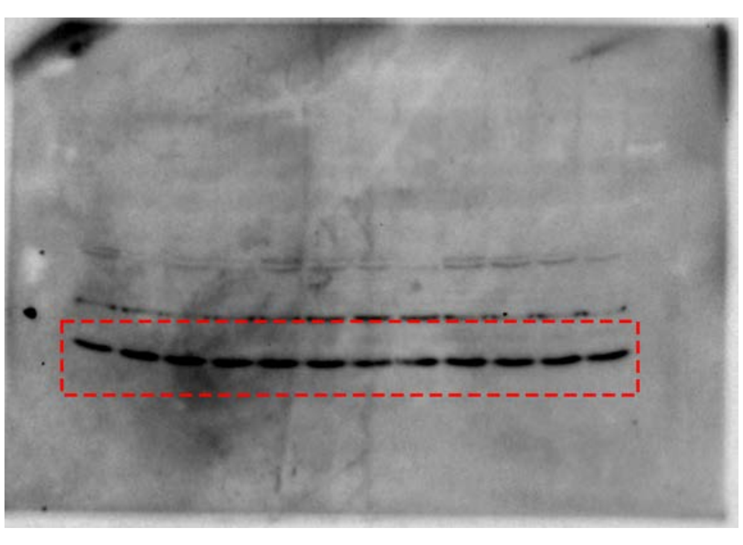

MCL1

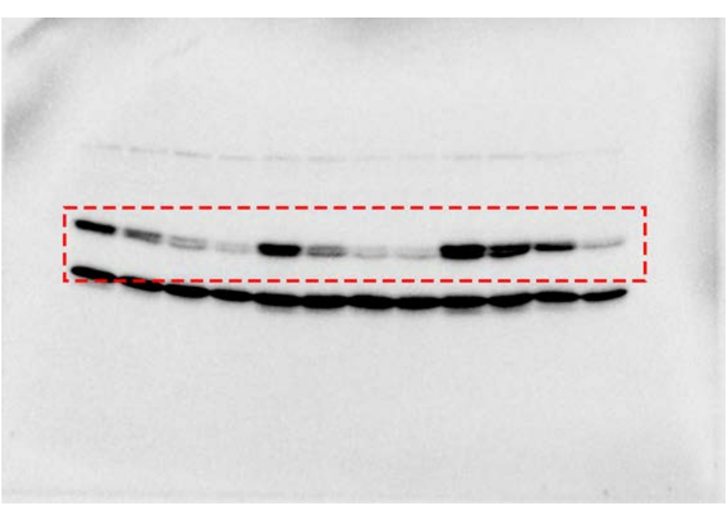

GAPDH

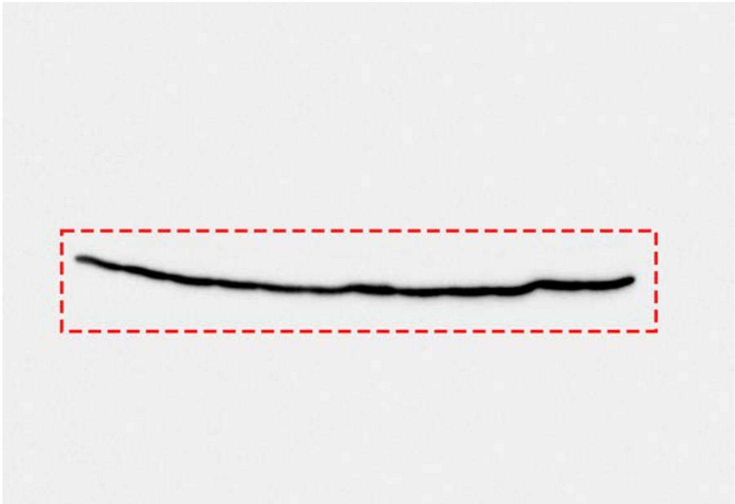

Fig 7B_Right

BCL-XL

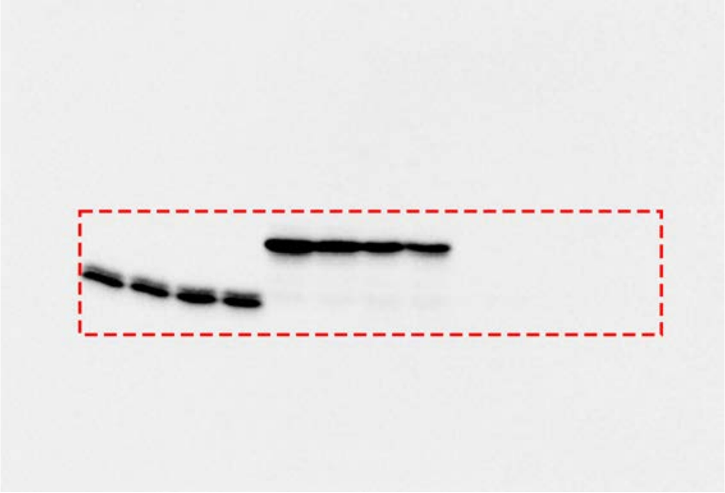

BCL2

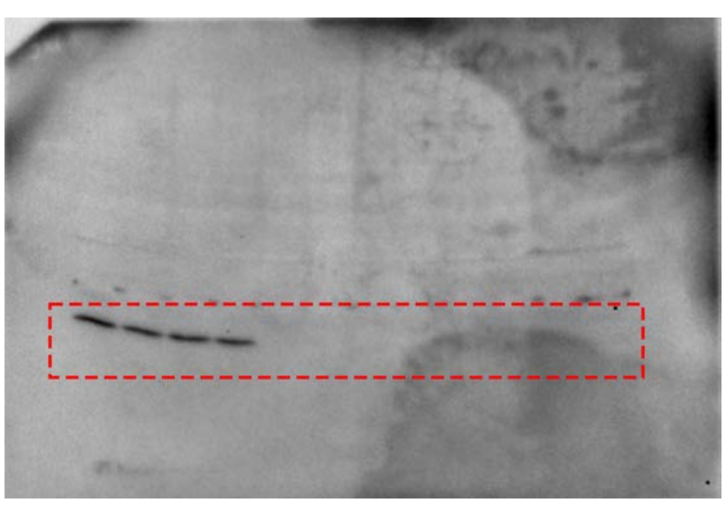

MCL1

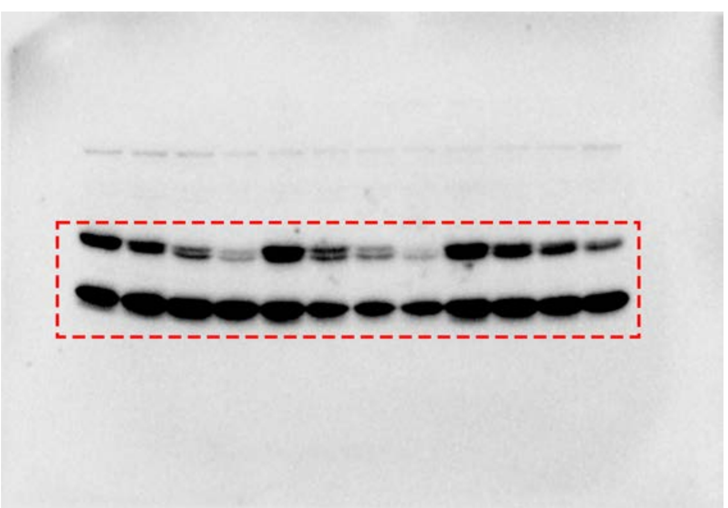

GAPDH

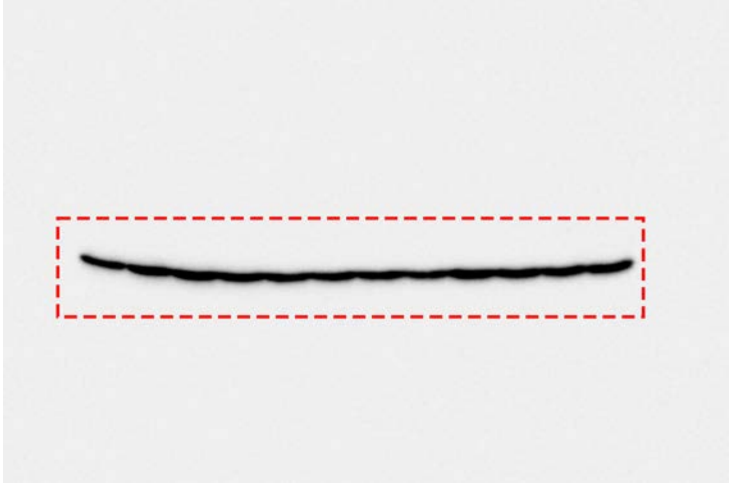

Fig 7C

MCL1

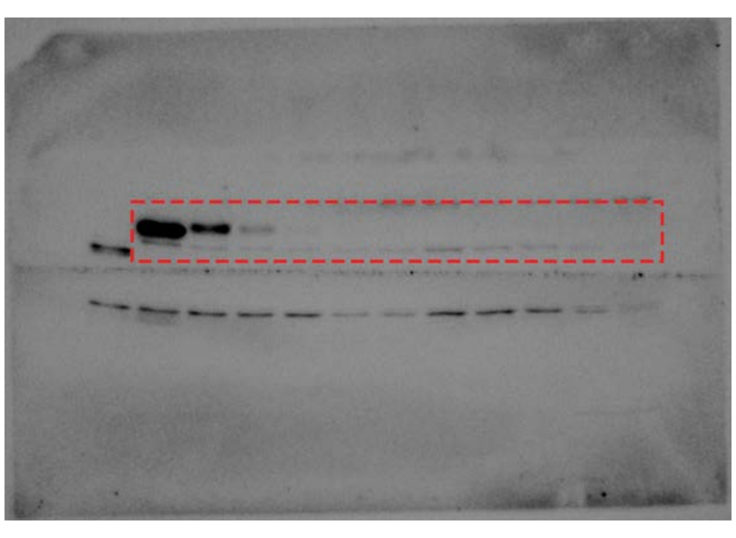

BCL-XL

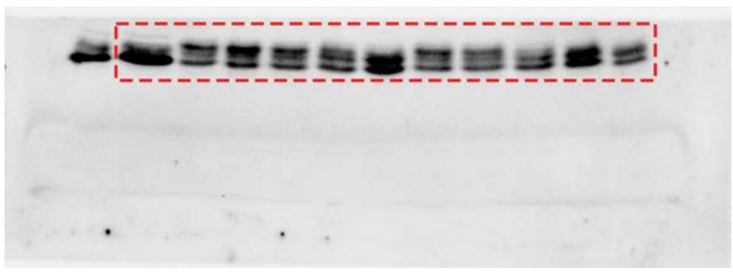

BCL2

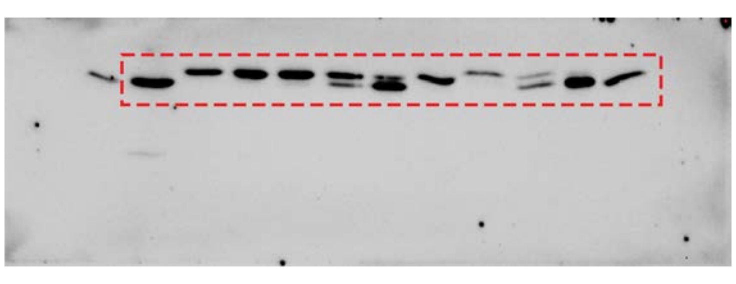

PARP-1
(Cleaved)

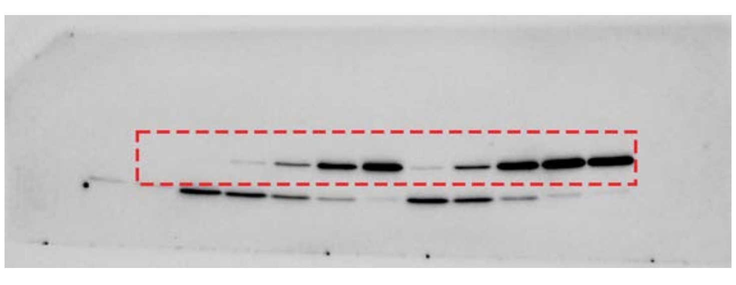

Actin

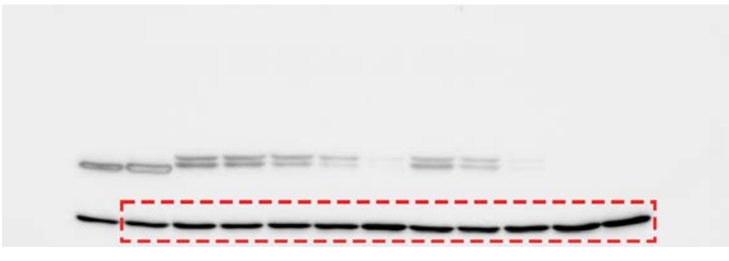

Fig 7D

MCL1

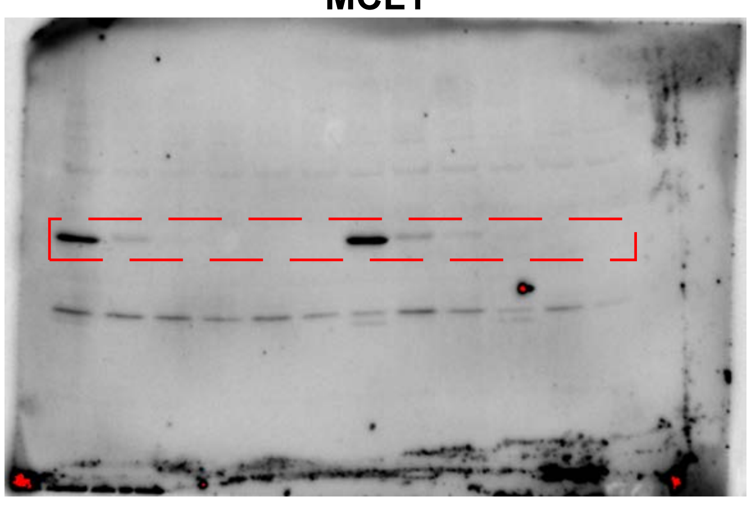

BCL-XL

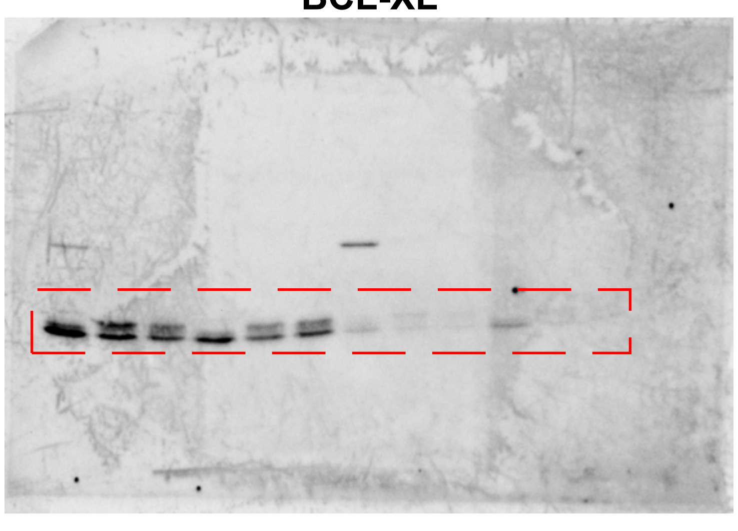

BCL2

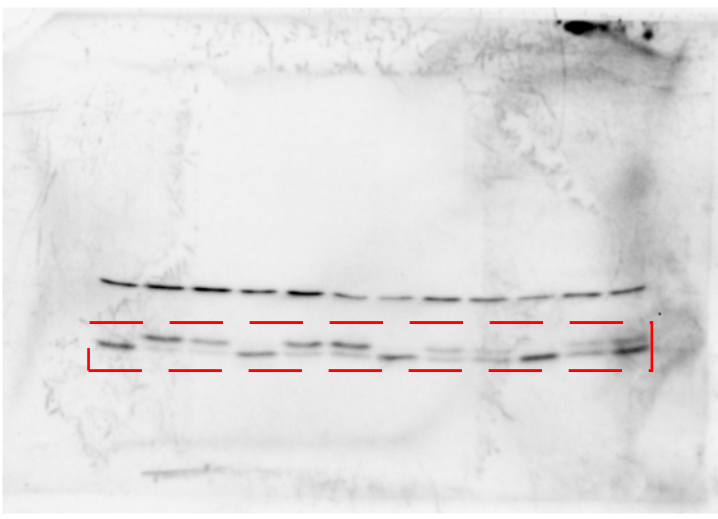

PARP-1
(Cleaved)

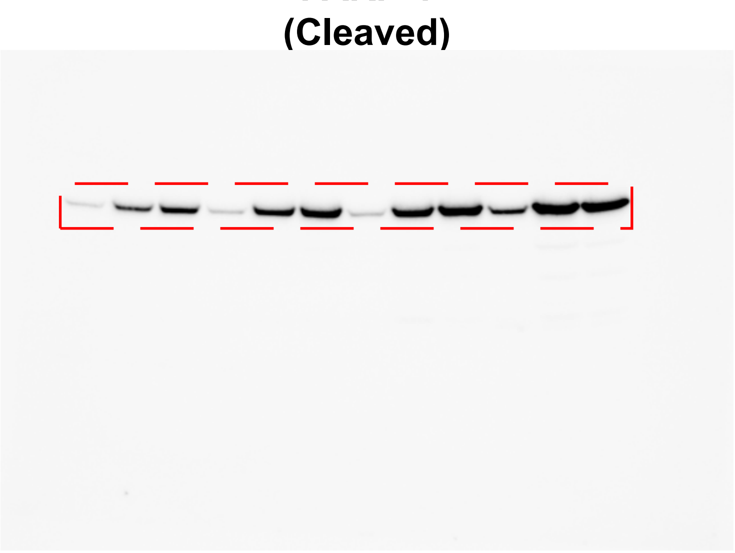

Actin

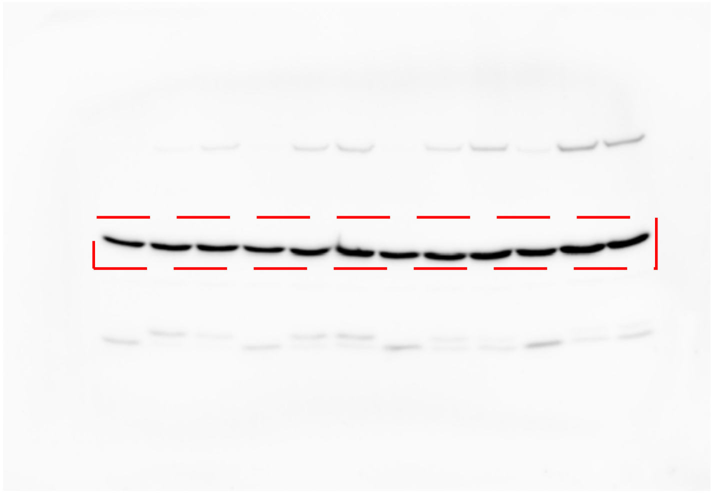

Original Western blots of Figure 7

Fig S1A

BCL-XL

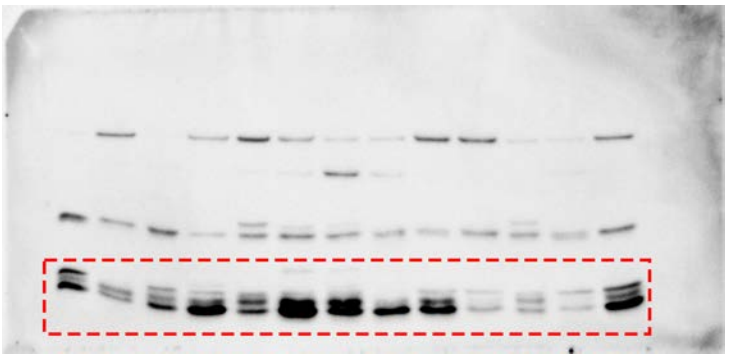

BCL2

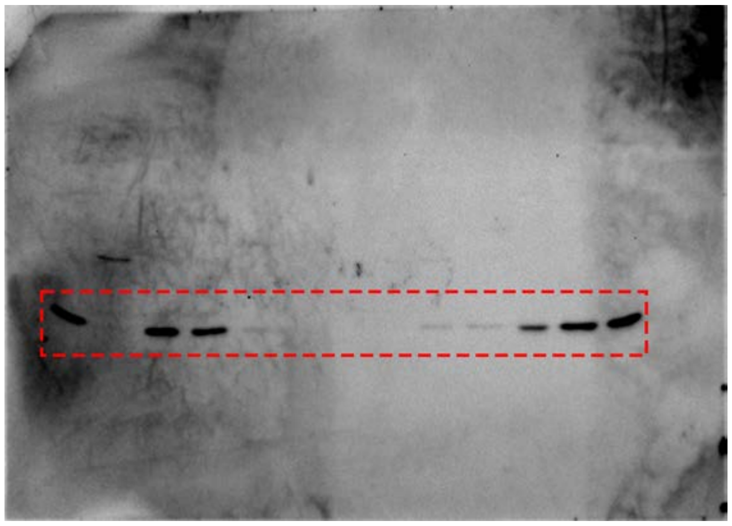

Actin

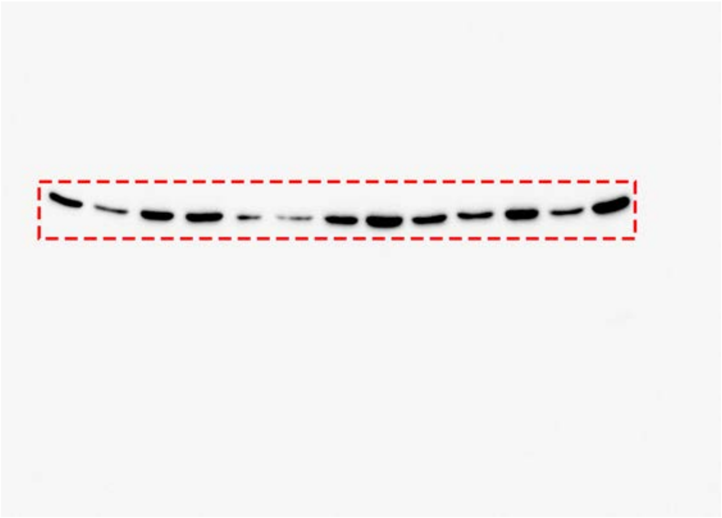

Original Western blots of Figure S1

Fig S2A

BCL-XL

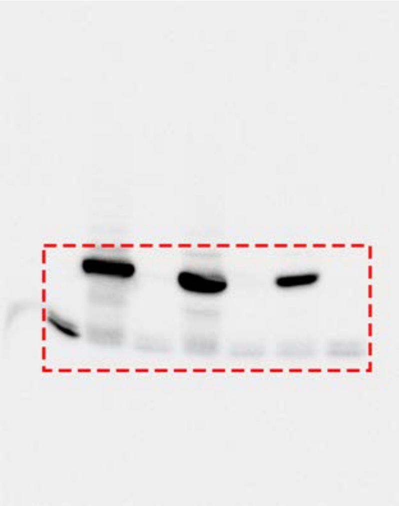

Actin

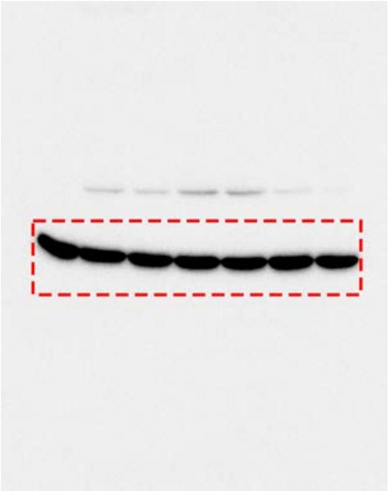

Fig S2B

BCL2

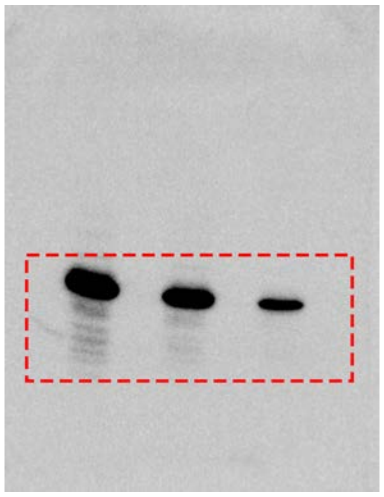

BCL2

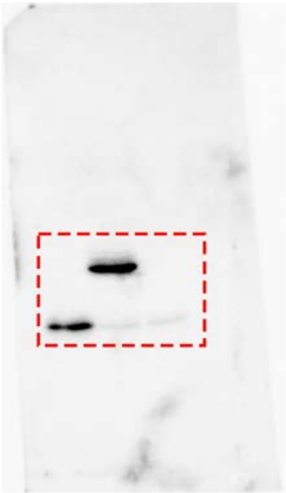

Actin

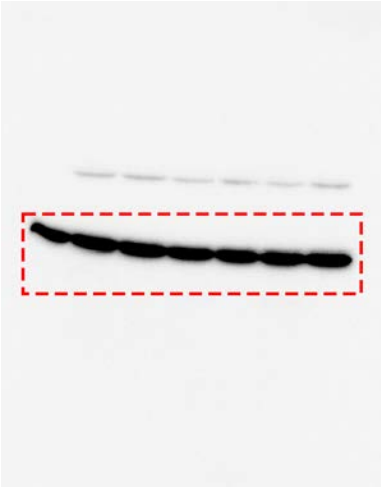

Actin

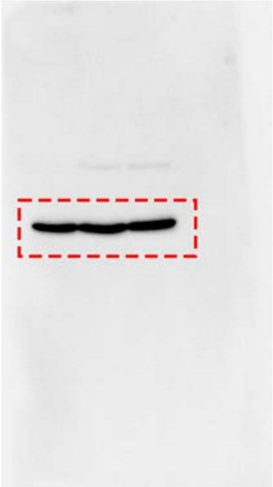

Original Western blots of Figure S2

Fig S3A

BCL-W

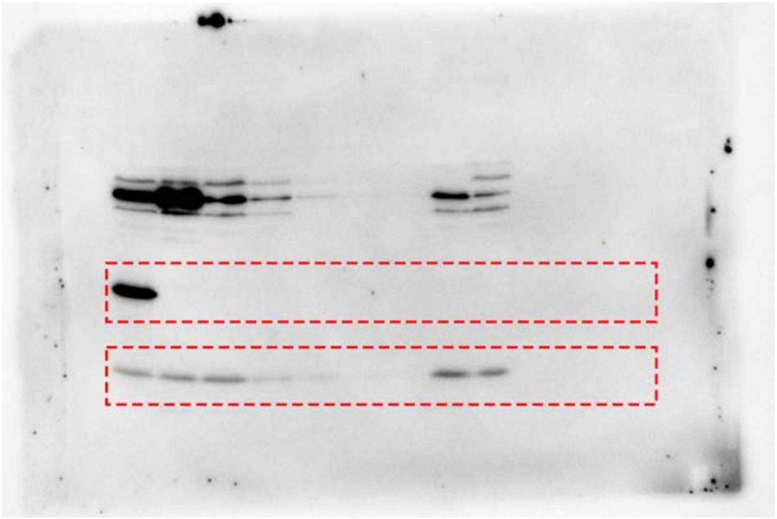

BCL2

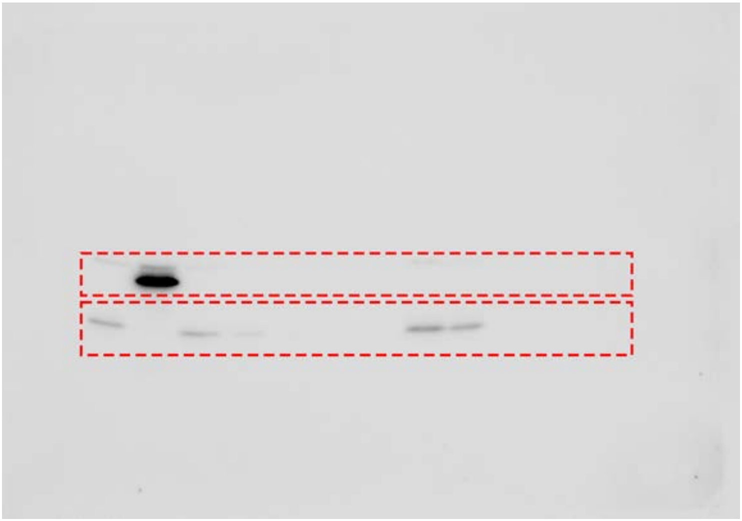

BCL-XL

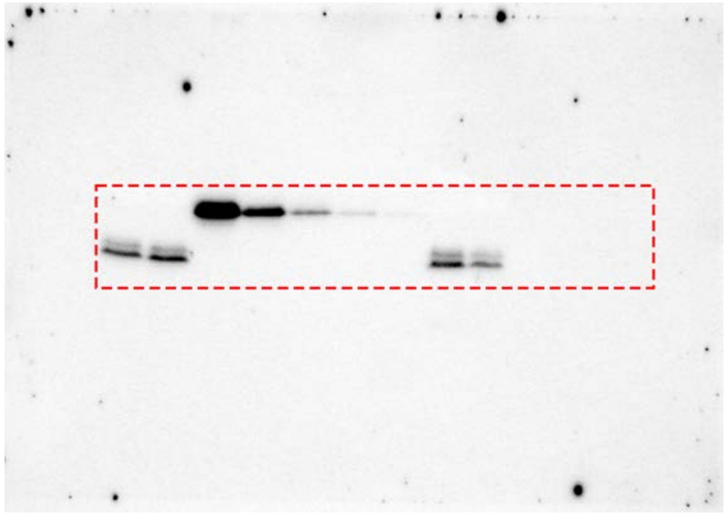

MCL1

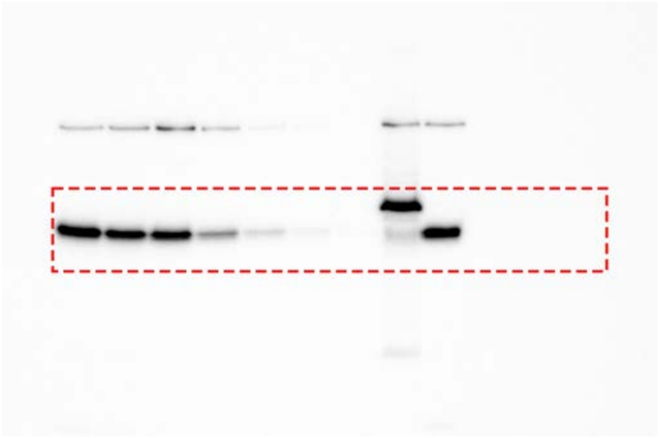

mAID

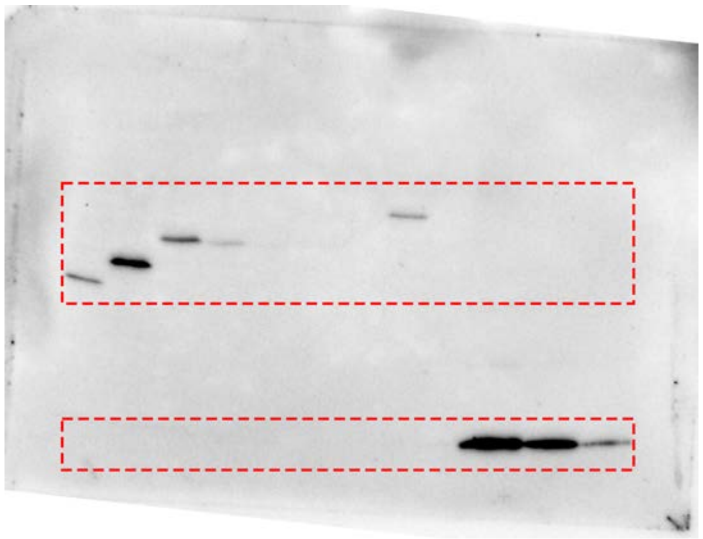

Actin

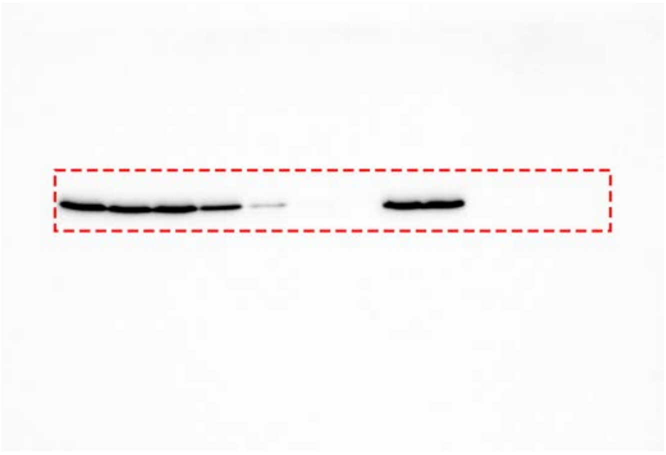

Original Western blots of Figure S3

Fig S5A

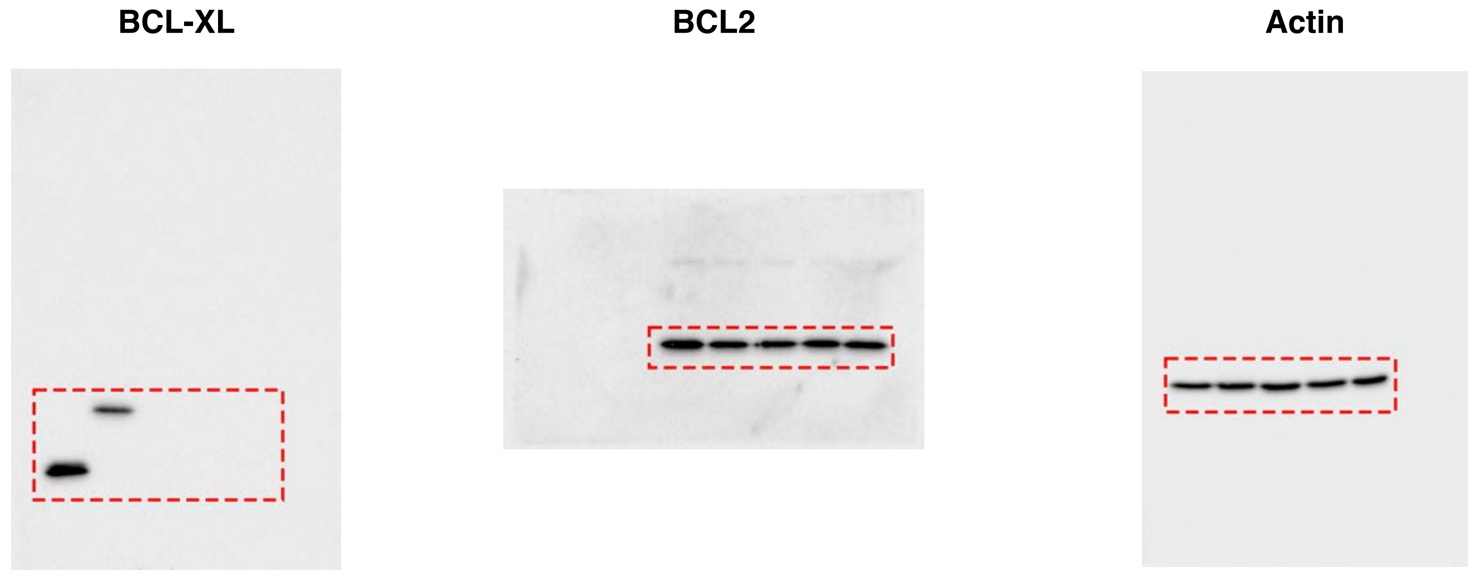

Fig S5B

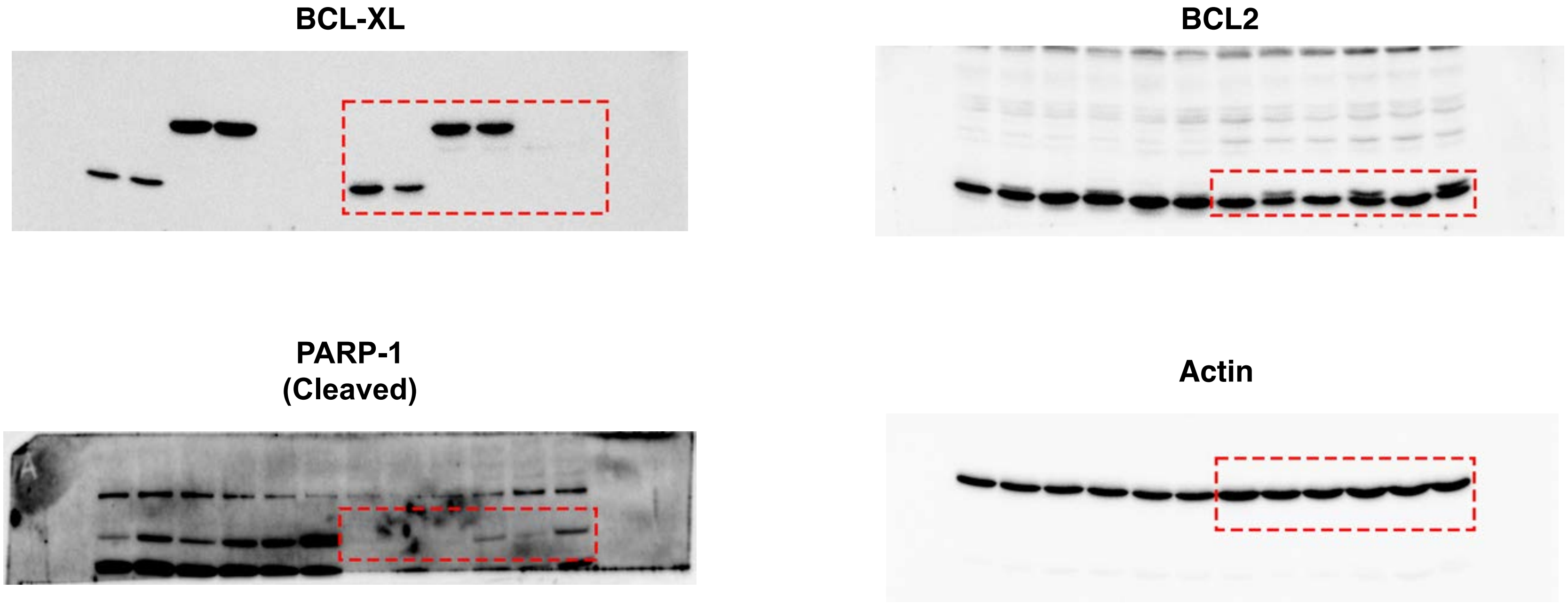

Original Western blots of Figure S5
